# Design, Synthesis, Fungicidal and Insecticidal Activities of Novel Diamide Compounds Combining Pyrazolyl and Polyfluoro-Substituted Phenyl into Alanine or 2-Aminobutyric Acid Skeletons

**DOI:** 10.3390/molecules28020561

**Published:** 2023-01-05

**Authors:** Zhi-Yuan Xu, Tong Feng, Qing Liu, Hui-Ting Li, Wei Wei, Rong-Chuan Shi, Yi-Ming Cao, Shang-Zhong Liu

**Affiliations:** 1 Innovation Center of Pesticide Research, Department of Applied Chemistry, College of Science, China Agricultural University, Beijing 100193, China; 2 Key Laboratory of National Forestry and Grassland Administration on Pest Chemical Control, China Agricultural University, Beijing 100193, China

**Keywords:** biological assay, diamide, enzymatic activity, pyflubumide, synthesis

## Abstract

Thirty novel diamide compounds combining pyrazolyl and polyfluoro-substituted phenyl groups into alanine or 2-aminobutyric acid skeletons were designed and synthesized with pyflubumide as the lead compound to develop potent and environmentally friendly pesticides. The preliminary bioassay results indicated that the new compounds containing the para-hexa/heptafluoroisopropylphenyl moiety exhibit fungicidal, insecticidal, and acaricidal activities. This is the first time that the para-hexa/heptafluoroisopropylphenyl group is a key fragment of the fungicidal activity of new N-phenyl amide compounds. Most of the target compounds exhibited moderate to good insecticidal activity against *Aphis craccivora* at a concentration of 400 μg/mL, and some showed moderate activity at a concentration of 200 μg/mL; in particular, compounds I-4, II-a-10, and III-26 displayed higher than 78% lethal rates at 200 μg/mL. Compound II-a-14 exhibited a 61.1% inhibition at 200 μg/mL for *Tetranychus cinnabarinus*. In addition, some of the target compounds exhibited good insecticidal activities against *Plutella xylostella* at a concentration of 200 μg/mL; the mortalities of compounds I-1, and II-a-15 were 76.7% and 70.0%, respectively. Preliminary analysis of the structure–activity relationship (SAR) indicated that the insecticidal and acaricidal activities varied significantly depending on the type of substituent and substitution pattern. The fungicidal activity results showed that compounds I-1, II-a-10, II-a-17, and III-26 exhibited good antifungal effects. Enzymatic activity experiments and in vivo efficacy of compound II-a-10 were conducted and discussed.

## 1. Introduction

Global agricultural production needs to increase by 60–110% in the mid-21st century to meet the demands of population growth and increased consumption in 2020 [1], which means that a 2.4% annual agricultural crop production growth rate is required. However, the study in 2013 estimated that the yields of four important crops (i.e., rice, wheat, corn, and soybean) grow at 1.0%, 0.9%, 1.6%, and 1.3% per year, respectively, and actual annual increase in 2018–2021 for 4 crops were 0.9%, 1.3%, 1.9%, and 2.0%, respectively, which is lower than the required rate of 2.4% [2,3]. Plants carry out photosynthesis and provide food for many organisms on Earth. Humanity protected cultivated plants from being consumed by other species and against harmful pests, weeds, and diseases to minimize their influence on crop production since agriculture began. Pesticides are key substances for controlling pests in agriculture, and they have evolved into many highly active organic compounds from inorganic sulfur and other natural products. However, nowadays, pesticides cannot completely control the influence of pests on global crop production, even though advanced application technologies [4].

Furthermore, agricultural pests evolved to weaken the influence of pesticides on their growth. For instance, they gradually generate resistance through protein mutations and the metabolic capability to neutralize lethal chemicals [5]. Thus, new active ingredients with novel chemistry and action modes or advanced application technologies such as nanoparticles are needed to fight pests and to ensure the healthy growth of crops to a maximum extent so that agricultural cultivation can provide enough food for the increasing global population [6].

In agricultural activities, the economic loss caused by fungi exceeds that of other microorganisms, and the annual loss is estimated to exceed 200 billion dollars [7]. More than 19,000 different species of fungi can cause plant diseases; a range of epidemic diseases caused by them has deeply affected agricultural production [8,9]. Fungicides play a major role in plant disease control and crop quality improvement [10], and ensure the safety of food production and supplies for modern agriculture in limited cultivable areas [11,12].

Along with pesticide development, most organophosphate and carbamate insecticides have been phased out because of their high toxicity to non-target organisms, and the demand for some synthetic pyrethroids and nicotinic insecticides is shrinking because of insect pest resistance from long-term application and toxicity to honey bees or aquarium organisms. To effectively control insect pests that harm crops, especially rice borers, diamide insecticides, with novel chemistry and action modes, appeared after organophosphates, carbamates, and synthetic pyrethroids and nicotinic insecticides at the end of 20 century [13]. Flubendiamide from Nihon Nohyaku Co., Ltd. (Tokyo, Japan) was the first diamide insecticide with a phthalic diamide skeleton [14,15]. 

**Figure 1:**
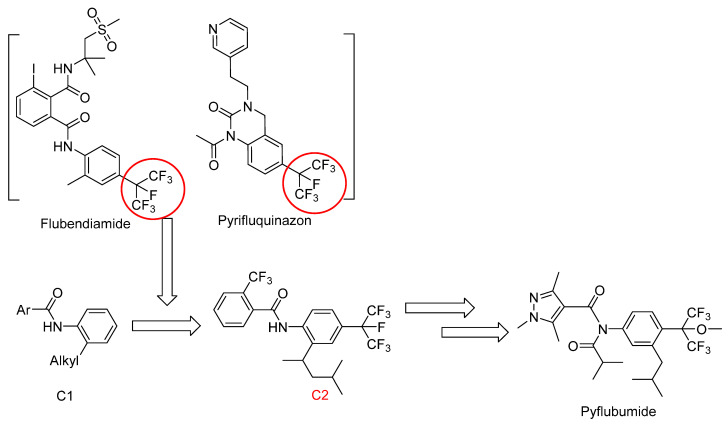
The creation process of pyflubumide.

Chlorantraniliprole, developed by Dupont using a structural modification of the diamide skeleton, was the second diamide insecticide with an anthranilic diamide skeleton [16,17]. Several diamides with phthalic or anthranilic diamide structural skeletons have been developed by different companies. Due to their high insecticidal activity, diamide insecticides are among the main insecticidal agrochemicals. Meanwhile, structural modifications based on phthalic and anthranilic diamide skeletons, as well as characteristic 4-hexafluoroisopropyl phenyl and 4-heptafluoroisopropylphenyl moieties, have attracted the attention of many research teams. For example, Katsuta et al. found the high insecticidal activity of meta-diamide based on optimizing flubendiamide and discovered broflanilide with an action mode different from phthalic or anthranilic diamide insecticides [18]. Nihon Nohyaku Co., Ltd. attempted to incorporate the hepta/hexafluoroisopropyl phenyl functional group into the skeleton of formamide fungicides (such as flubendiamide and pyrifluquinazon) to discover new fungicidal molecules. A new compound C2 containing N-2-methyl-4-heptafluoroisopropylphenyl exhibited low fungicidal activity, and finally found novel acaricide pyflubumide after lots of structure modification [19] (Figure 1). 

Pyflubumide displays excellent acaricidal activity against *Tetranychus urticae* and *Panonychus citri*, including strains that have developed resistance to conventional acaricides, and acts on the mitochondrial complex II in the respiratory chain of phytophagous mites [20]. Its safety against non-target arthropods is suitable for integrated pest management (IPM) programs. It is an interesting molecule combining the skeleton of formamide fungicide, functional fragment diamide bonds, and hexafluorophenyl in one insecticidal molecule; in particular, two amide bonds couple to one nitrogen atom [21]. 

**Figure 2:**
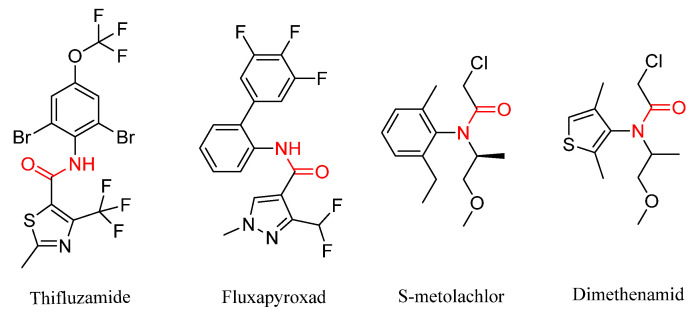
Chemical structures of representative amide pesticides.

**Figure 3:**
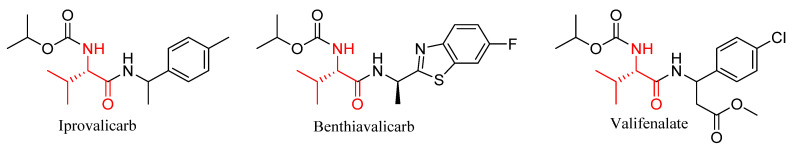
Chemical structures of representative pesticides containing aminoacid fragments.

**Figure 4:**
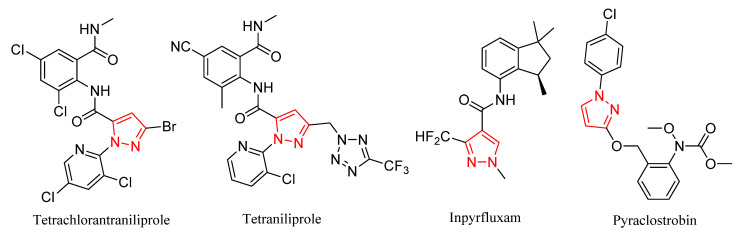
Commercial pesticides that contain pyrazole.

**Figure 5:**
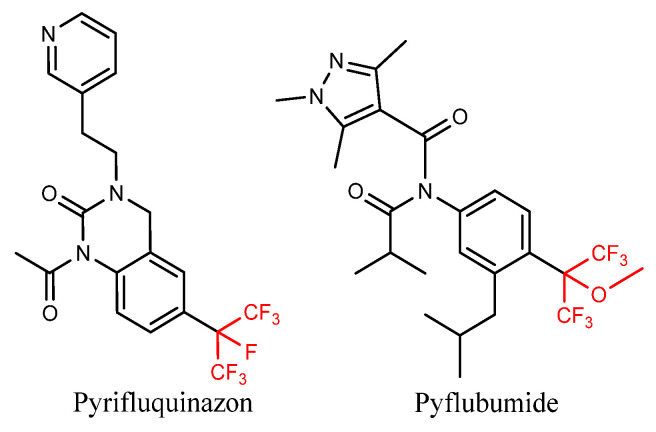
Commercial insecticides that contain heptafluoroisopropyl or hexafluoro-2-methoxypropyl.

Amide bonds often appear in small organic compounds and macromolecules. In addition to the covalent bonds constructing diamide insecticides, they are also vital functional groups in many other pesticide active ingredients, such as the succinate dehydrogenase inhibitor (SDHI) fungicides thifluzamide, fluxapyroxad, N-aryl amide herbicides S-metolachlor, and dimethenamid [22,23,24,25] (Figure 2). Amino acids are small molecules that are suitable for building amide bonds and are often found in pesticides. For example, D-valine appears in the fungicides iprovalicarb, benthiavalicarb, and valifenalate [26,27,28] (Figure 3). Pyrazole in nitrogen-containing heterocycles is of great importance in the field of medicinal chemistry [29,30], and it also provides many pesticide molecules with a variety of biological activities, such as the insecticides tetrachlorantraniliprole and tetraniliprole, and fungicides inpyrfluxam and pyraclostrobin [31,32,33,34] (Figure 4). Heptafluoroisopropyl and hexafluoro-2-methoxypropylphenyl are often the key functional fragments in recent insecticide molecules such as pyrifluquinazon and pyflubumide [35] (Figure 5).

**Figure 6:**
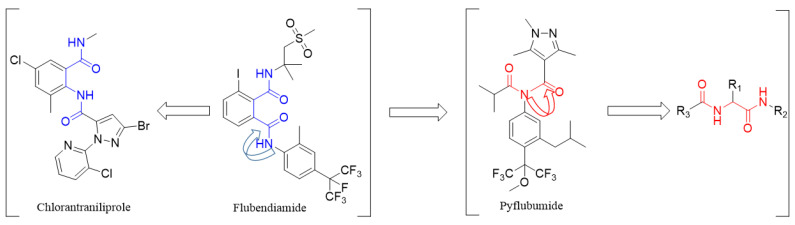
The design strategy of target compounds.

Inspired by the strategy employed by Dupont to modify the flubendiamide structure and finding chlorantraniliprole by switching the amide linkage of phthalic acid diamides, we were motivated to use pyflubumide as the lead structure and design new structures via functional group migration. In this study, small amino acid molecules were employed as diamide bond skeletons to combine functional fragments substituted with pyrazolyl and polyfluoro isopropylphenyl into one molecule for the design and synthesis of new pesticidal molecules (Figure 6).

The insecticidal, acaricidal, and fungicidal activities of all the new compounds were investigated. To the best of our knowledge, this is the first study to demonstrate the potent fungicidal activity of novel diamide derivatives containing pyrazolyl and hepta/hexafluoroisopropyl-substituted phenyl groups.

## 2. Results and Discussion

### 2.1. Chemistry

**Scheme 1:**
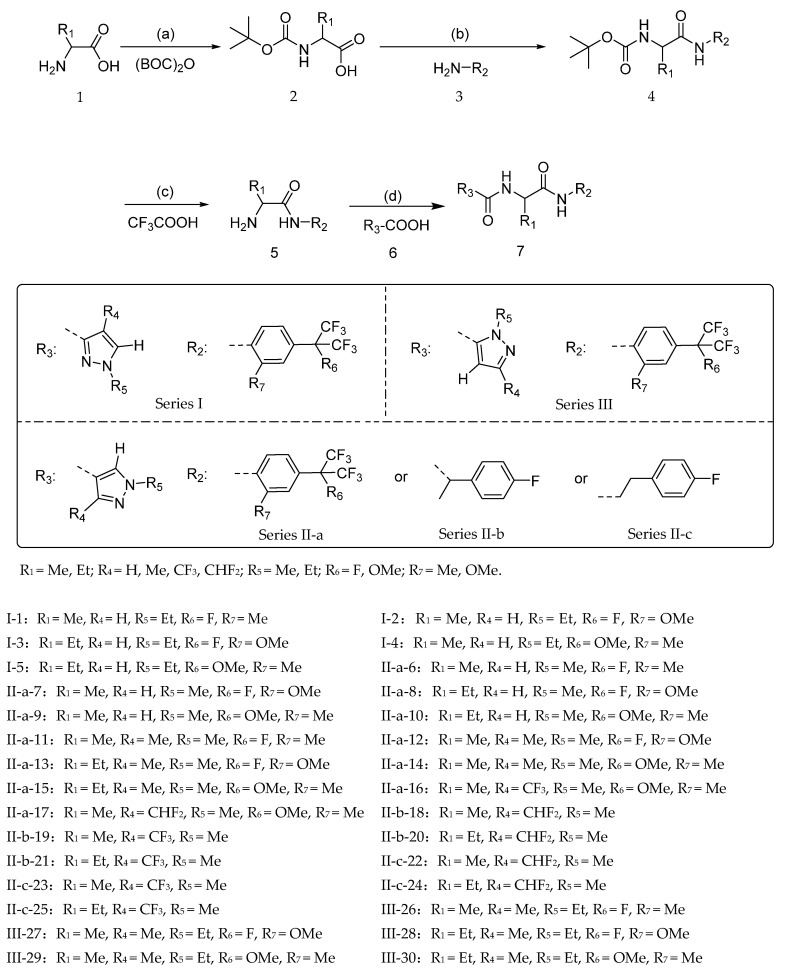
General synthetic route for compounds. Reagents and conditions: (**a**) NaOH, 1,4-Dioxane, H_2_O, 0–25 °C; (**b**) DCC, CH_2_Cl_2_, 0–25 °C; (**c**) CH_2_Cl_2_, 25 °C; (**d**) EDCl, HOBT, DIEA, CH_2_Cl_2_, 0–25 °C.

The designed compounds were prepared using two alpha-amino acids as starting raw materials to construct two amide bonds. The synthetic route must include the protection and deprotection steps of one active group because alpha-amino contains two reactive groups, as outlined in Scheme 1, in which the target compounds were prepared in four steps. Commercially available DL-alanine or DL-2-aminobutyric acid (1) was first reacted with di-tert-butyl dicarbonate in water and 1,4-dioxane to protect the amino group and afford intermediate 2 in good yields. Second, intermediate 2 and ingredient 3 were mixed in dichloromethane and subjected to a condensation reaction to obtain intermediate 4. Third, the protective amino group of intermediate 4 was removed using trifluoroacetic acid to obtain intermediate 5, which can be used in the next reaction without further purification. Finally, in the presence of the condensing agent 1-ethyl-3-(3-dimethylaminopropyl)carbodiimide (EDCI), intermediate 5 was reacted with substituted pyrazolecarboxylic acids to obtain the final product at a good yield.

### 2.2. Structure–Activity Relationship

#### 2.2.1. Insecticidal and Acaricidal Activity

As the primary objective of this study, preliminary tests of the insecticidal and acaricidal activities of target compounds I-1–III-30 against the pest species *Aphis craccivora*, *Tetranychus cinnabarinus*, and *Plutella xylostella* were carried out. The insecticidal and acaricidal activities of each compound are shown in Table 1.

First, the insecticidal activity of the target compounds I-1–III-30 against *Aphis craccivora* was determined, and imidacloprid was tested under the same conditions as a comparison compound. The results summarized in Table 1 indicate that compounds I-4, II-a-10, II-a-14, and III-26 exhibited good insecticidal activities against *Aphis craccivora* with more than 90.0% mortality at 400 μg/mL, which was comparable to that of the control imidacloprid (100.0%). When the concentration of the compounds was reduced from 400 to 200 μg/mL, compounds I-4, II-a-10, and III-26 still showed good mortality against *Aphis craccivora*. However, a significant decrease in insecticidal activity was observed when the concentration was reduced from 200 to 100 μg/mL. Furthermore, the insecticide activity of these compounds varied drastically according to the type and pattern of substitution on the pyrazolyl group, alkyl groups between the two amide bonds, and the substituted phenyl rings. For the effect of methyl and ethyl groups in pyrazolyl in the series of I-1–III-30 compounds, when the substituent was ethyl, such as I-4 and III-26, they exhibited higher insecticidal potency than the corresponding analog with methyl, such as II-a-9 and II-a-11. No significant differences were observed in the effect of the alkyl group between the two amide bonds in the I-1–III-30 series of compounds. For the effect of substituents on the phenyl ring in the series of I-1–III-30 compounds, the compounds I-4, II-a-10, and II-a-14 with the methoxy-substituted hexafluoroisopropyl group gave increased insecticidal activity compared to I-1, II-a-8, and II-a-11 with the heptafluoroisopropyl group and II-b-19 and II-c-22 with para-fluorobenyl group or para-fluorophenylethyl group instead of para-hexa/heptafluorophenyl group. In addition, compounds with a methyl group at the 2-position of the phenyl ring displayed relatively better insecticidal activity than the corresponding compounds with a methoxy group at the 2-position of the phenyl ring.

Most compounds exhibited acaricidal activity against *Tetranychus cinnabarinus* (Table 1). Among the target compounds, some possessed good acaricidal activity against *Tetranychus cinnabarinus* at a concentration of 400 μg/mL. For instance, the mortalities of compounds I-5, II-a-11, II-a-14, III-26, and III-28 against *Tetranychus cinnabarinus* were more than 70.0%, which were lower than that of the control fenpyroximate (100.0%). When the concentration was reduced to 200 μg/mL, compound II-a-14 showed a 61.1% inhibition rate. Based on the structure–activity study, we found that when the substituents in pyrazolyl in the series of I-1–III-30 compounds were ethyl or two methyl groups, such as I-5, II-a-11, and III-28, they exhibited higher acaricidal potency than the corresponding analog with one methyl, such as II-a-6, II-a-10, and II-a-13. Ethyl groups were slightly more abundant than methyl groups because of the effect of the alkyl group between the two amide bonds. For the effect of substituents on the phenyl ring, from the acaricidal activity against *Tetranychus cinnabarinus*, the compounds I-5 and II-a-14 with the methoxy-substituted hexafluoroisopropyl group and the compounds II-a-11, III-26 and III-28 with the heptafluoroisopropyl group gave better acaricidal activity than the compounds II-c-24 and II-c-25 with a para-fluorobenyl group or para-fluorophenylethyl group, respectively. Compounds I-4, II-a-10, and II-a-14, with a methyl group at the 2-position of the phenyl ring, displayed better acaricidal activity than the corresponding compounds with a methoxy group at the 2-position of the phenyl ring.

All compounds exhibited insecticidal activity against *Plutella xylostella* (Table 1). As can be seen, some target compounds demonstrated excellent insecticidal activities against *Plutella xylostella* at a concentration of 400 μg/mL; for instance, compounds I-1, II-a-15, and III-30 all had inhibition rates over 80.0%, which were lower than that of the control flubendiamide (100.0%). When the concentration was reduced to 200 μg/mL, the mortality rates of compounds I-1 and II-a-15 were 76.7% and 70.0%, respectively. Based on the structure–activity study, we found that the effect of substituted pyrazolyl in the series of I-1–III-30 compounds was the same as that of the other two insects. No significant changes were found in the effect of the alkyl group between the two amide bonds in compounds I-1–III-30. Regarding the effect of substituents on the phenyl ring, based on insecticidal activity against *Plutella xylostella*, compounds II-a-15 and III-30 with the methoxy-substituted hexafluoroisopropyl group showed better insecticidal activity than compounds II-a-13 and III-28 with the heptafluoroisopropyl group and compounds II-b-19 and II-c-24 with the para-fluorobenyl group or para-fluorophenylethyl group, respectively. Regarding the effect of substituents on the phenyl ring on insecticidal activity against *Plutella xylostella*, compounds I-1, II-a-15, and III-30 with a methyl group at the 2-position of the phenyl ring displayed relatively better insecticidal activity than the corresponding compounds with a methoxy group at the 2-position of the phenyl ring.

#### 2.2.2. Fungicidal Activity

Fluxapyroxad was selected as a positive control because of its similarity to the target compounds containing two amide bonds. The in vitro antifungal activities of target compounds I-1–III-30 were assessed at 50 μg/mL (Table 2). Compound II-a-10 displayed more than 80% growth inhibition activity against *Cytospora* sp., better than the positive control fluxapyroxad (78%), In addition, compound III-26 showed slightly higher antifungal activities against *B. cinerea* than that of fluxapyroxad (84%). Most compounds also exhibited better fungicidal activity against *F. graminearum* than that of fluxapyroxad (16%), and compounds I-2, II-a-7, II-a-12, and III-28 showed slightly higher antifungal activities against *P. aphanidermatum* than fluxapyroxad (31%). Furthermore, compounds I-1, II-a-10, and II-a-17 also displayed high growth inhibition activities against *S. sclerotiorum*, similar to fluxapyroxad (90%). However, the compounds with para-fluorobenyl or para-fluorophenylethyl groups displayed almost 0% growth inhibition activity against *Cytospora* sp. and *P. aphanidermatum*. There was no sufficient inhibitory effect on several other fungi; therefore, polyfluoride-substituted groups are necessary for fungicidal activity. Thus, the para-hexa/heptafluoroisopropylphenyl group in the newly synthesized compounds is a key fragment to provide the fungicidal activity of new compounds. This is the first report on the significant effect of the para-hexa/heptafluoroisopropylphenyl group on the fungicidal activity of organic compounds.

**Figure 7:**
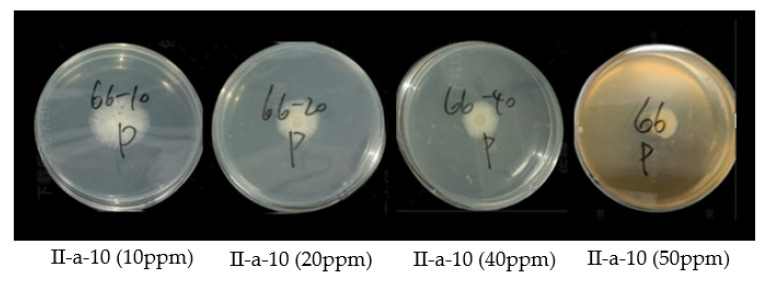
In vitro antifungal activities of compound II-a-10 against *Cytospora* sp.

Compounds with more than 80% inhibition at 50 μg/mL were further studied to obtain their EC_50_ values and explore fungicidal potency. As shown in Table 3 and Figure 7, these compounds showed relatively lower fungicidal activities in vitro against *Cytospora* sp., *B. cinerea*, and *S. sclerotiorum* than fluxapyroxad.

### 2.3. Enzyme Activity Assay

**Figure 8:**
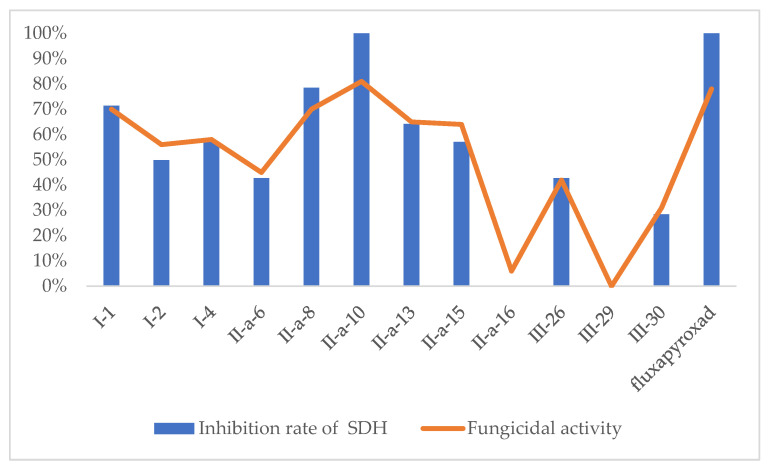
Comparison of compounds for SDH inhibition and fungicidal activity against *Cytospora* sp.

The inhibitory activities of the target compounds against succinate dehydrogenase were tested based on their similarity to amide fungicides. Twelve compounds with different fungicidal activities against *Cytospora* sp. were selected, and their inhibitory activities against succinate dehydrogenase of *Cytospora* sp. were determined at 100 μM. The fungicidal activity against *Cytospora* sp. from mycelium growth inhibition was consistent with the enzymatic inhibitory activities; for instance, compounds II-a-10 and fluxapyroxad showed 100% enzymatic inhibition with the highest fungicidal activity (Figure 8). This comparison indicates that the prepared compounds probably inhibit the respiration of pathogenic fungi and thus achieve a fungicidal effect. 

### 2.4. In Vivo Curative Effect of Compound II-a-10 against Cytospora sp.

**Figure 9:**
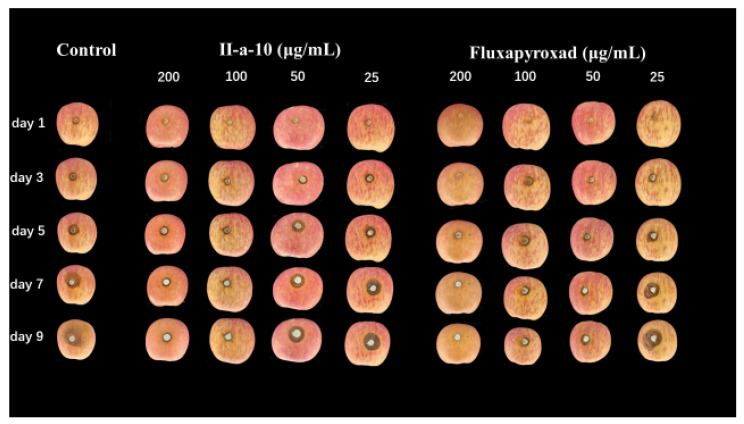
In vivo curative antifungal activities of II-a-10 against *Cytospora* sp.

To further verify the fungicidal activity of compound II-a-10, the in vivo curative effect of compound II-a-10 against *Cytospora* sp. was tested on apples. As illustrated in Figure 9 and Table 4, compound II-a-10 displayed acceptable curative activity in vivo against *Cytospora* sp., and its fungicidal activity was comparable to that of the commercial fungicide fluxapyroxad. For example, treatment with compound II-a-10 at a concentration of 25 μg/mL resulted in 59.46% curative efficacy 9 d after transplantation. However, the curative activity of fluxapyroxad reached 53.38% at 25 μg/mL.

## 3. Materials and Methods

### 3.1. Instruments and Materials

All solvents were obtained from commercial sources and were used without further processing. The melting points of all the compounds were determined using a B-III microscope (Beijing Technical Instrument Co., Beijing, China) and were uncorrected. Nuclear magnetic resonance (NMR) spectra were recorded using a Bruker AM-500 spectrometer. High-resolution mass spectra (HRMS) were obtained using an Agilent 6540 QTOF instrument.

### 3.2. Synthesis

The general synthetic procedure for intermediate 2 was as follows. Sodium hydroxide (400 mmol) was added to a mixture of compound 1 (200 mmol) in water (100 mL) and 1,4-dioxane (100 mL) at 0 °C while stirring, and the mixture was stirred for 15 min. Di-tert-butyl dicarbonate (200 mmol) was then added dropwise, and the mixture was stirred at 0 °C for 45 min. The ice bath was then removed and the temperature of the reaction mixture was allowed to rise to 25 °C. The reaction was monitored using TLC. After completion, the reaction mixture was concentrated in vacuum to remove the organic solvent, and the aqueous layer was adjusted with citric acid (pH 2–3) and extracted with ethyl acetate. The combined organic phase was dried over anhydrous sodium sulfate, and concentrated at reduced pressure, resulting in intermediate 2 with yields 95.1% to 96.3% [36].

The general synthetic procedure for intermediate 4 was as follows. To a mixture of intermediate 2 (30 mmol) and compound 3 (30 mmol) that prepared according to literature methods without purification [37,38] in dichloromethane (50 mL), dicyclohexylcarbodiimide (DCC, 45 mmol) in dichloromethane (20 mL) was added dropwise at 0 °C. The reaction mixture was warmed to 25 °C and stirred overnight until the reaction was complete. The mixture was then filtered, and the filtrate was washed with a saturated sodium bicarbonate solution and saturated brine, dried over anhydrous sodium sulfate, and concentrated in vacuum. The residue was purified by flash chromatography on silica gel to give intermediate 4 with yields of 53.4% to 55.2% [39].

The general synthesis procedure for intermediate 5 was as follows. Intermediate 4 (15 mmol) was dissolved in dichloromethane (45 mL) and trifluoroacetic acid (45 mL) and stirred at room temperature. When the reaction was complete, as monitored by TLC, the reaction mixture was concentrated in vacuum, the concentrate was adjusted to pH 7 with sodium bicarbonate, and then extracted with ethyl acetate. The combined organic phase was filtered after drying over anhydrous sodium sulfate and concentrated in vacuum to give intermediate 6 with yields of 95.3% to 96.4% [40].

The general synthesis procedure for compounds I-1–III-30 was as follows. Intermediate 5 (2.76 mmol) was dissolved in dichloromethane (50 mL) at 0 °C and added to EDCI (2.76 mmol), 1-hydroxybenzotriazole (2.76 mmol) and *N*,*N*-diisopropylethylamine (2.76 mmol). After the mixture was stirred for 60 min at 0 °C, compound 6 (2.76 mmol) was added, and the mixture was stirred at 25 °C until the reaction was complete, as indicated by TLC. The reaction mixture was washed with a saturated citric acid solution, sodium bicarbonate solution, and saturated brine. The obtained organic phase was dried over anhydrous sodium sulfate, filtered, and concentrated in vacuum. The residue was purified by flash chromatography on silica gel to give compounds (I-1–III-30) with yields of 43–58% [41]. ^1^H and ^13^C NMR spectral data of compounds I-1–III-30 is in supplementary materials.

1-Ethyl-N-(1-((2-methyl-4-(perfluoropropan-2-yl)phenyl)amino)-1-oxopropan-2-yl)-1H-pyrazole-3-carboxamide I-1. White solid (54% yield), m.p. 120.3–122.4 °C. ^1^H NMR (500 MHz, DMSO-*d*_6_) δ 9.72 (s, 1H), 8.13 (d, *J* = 7.4 Hz, 1H), 7.88 (d, *J* = 2.3 Hz, 1H), 7.86 (d, *J* = 8.6 Hz, 1H), 7.54 (s, 1H), 7.51 (d, *J* = 8.6 Hz, 1H), 6.73 (d, *J* = 2.3 Hz, 1H), 4.81 (m, 1H), 4.25 (q, *J* = 7.3 Hz, 2H), 2.36 (s, 3H), 1.52 (d, *J* = 7.2 Hz, 3H), 1.45 (t, *J* = 7.3 Hz, 3H). ^13^C NMR (126 MHz, DMSO) δ 172.02, 161.64, 145.94, 139.68, 132.55, 131.61, 127.55 (d, *J*_CF_ = 10.0 Hz, Ar-C_3_), 125.07, 123.73 (d, *J*_CF_ = 10.6 Hz, Ar-C_5_), 121.52 (d, *J*_CF_ = 20.4 Hz, Ar-C_4_), 120.76 (qd, ^1^*J*_CF_ = 287.0 Hz, ^2^*J*_CF_ = 28.0 Hz, CF_3_), 106.41, 91.55 (dsept, ^1^*J*_CF_ = 200.3 Hz, ^2^*J*_CF_ = 32.6 Hz, CF(CF_3_)_2_), 49.15, 47.23, 18.86, 18.25, 15.84. HRMS calcd. for C_19_H_19_F_7_N_4_O_2_ ([M+H]^+^), 469.1474; found, 469.1742.

1-Ethyl-N-(1-((2-methoxy-4-(perfluoropropan-2-yl)phenyl)amino)-1-oxopropan-2-yl)-1H-pyrazole-3-carboxamide I-2. Yellow solid (53% yield), m.p. 80.3–82.2 °C. ^1^H NMR (500 MHz, Chloroform-*d*) δ 8.82 (s, 1H), 8.46 (d, *J* = 8.6 Hz, 1H), 7.39 (d, *J* = 2.3 Hz, 2H), 7.16 (d, *J* = 8.7 Hz, 1H), 7.01 (s, 1H), 6.79 (d, *J* = 2.3 Hz, 1H), 4.85 (m, 1H), 4.16 (q, *J* = 7.3 Hz, 2H), 3.85 (s, 3H), 1.56 (d, *J* = 7.1 Hz, 3H), 1.47 (t, *J* = 7.4 Hz, 3H). ^13^C NMR (126 MHz, CDCl_3_) δ 169.72, 161.30, 147.29, 144.45, 129.19, 129.19, 120.50 (d, *J*_CF_ = 21.1 Hz, Ar-C_4_), 119.57 (qd, ^1^*J*_CF_ = 215.5 Hz, ^2^*J*_CF_ = 29.0 Hz, CF_3_), 118.72, 117.64 (d, *J*_CF_ = 9.9 Hz, Ar-C_3_), 106.33 (d, *J*_CF_ = 12.4 Hz, Ar-C_5_), 105.74, 90.33 (dsept, ^1^*J*_CF_ = 201.4 Hz, ^2^*J*_CF_ = 32.8Hz, CF(CF_3_)_2_), 55.02, 48.48, 46.57, 16.42, 14.40. HRMS calcd. for C_19_H_19_F_7_N_4_O_3_ ([M+H]^+^), 485.1424; found, 485.1427.

1-Ethyl-N-(1-((2-methoxy-4-(perfluoropropan-2-yl)phenyl)amino)-1-oxobutan-2-yl)-1H-pyrazole-3-carboxamide I-3. Yellow solid (56% yield), m.p. 110.6–111.5 °C. ^1^H NMR (500 MHz, Chloroform-*d*) δ 8.60 (s, 1H), 8.41 (d, *J* = 8.6 Hz, 1H), 7.37 (d, *J* = 8.2 Hz, 1H), 7.32 (d, *J* = 2.3 Hz, 1H), 7.10 (d, *J* = 8.7 Hz, 1H), 6.95 (s, 1H), 6.72 (d, *J* = 2.3 Hz, 1H), 4.64 (td, *J* = 7.8, 6.3 Hz, 1H), 4.10 (q, *J* = 7.3 Hz, 2H), 3.79 (s, 3H), 2.02 (dq, *J* = 13.8, 7.3 Hz, 1H), 1.80 (dq, *J* = 14.1, 7.5 Hz, 1H), 1.41 (t, *J* = 7.3 Hz, 3H), 0.98 (t, *J* = 7.4 Hz, 3H). ^13^C NMR (126 MHz, CDCl_3_) δ 169.51, 162.58, 147.64, 147.17, 131.27, 128.71, 120.88 (d, *J*_CF_ = 21.1 Hz, Ar-C_4_), 119.54 (qd, ^1^*J*_CF_ = 286 Hz, ^2^*J*_CF_ = 26.5 Hz, CF_3_), 118.68, 117.69 (d, *J*_CF_ = 10 Hz, Ar-C_3_), 114.01, 106.35 (d, *J*_CF_ = 12.4 Hz, Ar-C_5_), 90.28 (dsept, ^1^*J*_CF_ = 201.5 Hz, ^2^*J*_CF_ = 32.8 Hz, CF(CF_3_)_2_), 55.02, 54.12, 37.88, 24.65, 12.58, 8.95. HRMS calcd. for C_20_H_21_F_7_N_4_O_3_ ([M+H]^+^), 499.1580; found, 499.1582.

1-Ethyl-N-(1-((4-(1,1,1,3,3,3-hexafluoro-2-methoxypropan-2-yl)-2-methylphenyl)amino)-1-oxopropan-2-yl)-1H-pyrazole-3-carboxamide I-4. Yellow solid (55% yield), m.p. 99.8–100.9 °C. ^1^H NMR (500 MHz, Chloroform-*d*) δ 8.88 (s, 1H), 8.04 (d, *J* = 8.7 Hz, 1H), 7.33 (d, *J* = 2.4 Hz, 1H), 7.29 (d, *J* = 12.1 Hz, 2H), 7.25 (s, 1H), 6.70 (d, *J* = 2.3 Hz, 1H), 4.82 (m, 1H), 4.09 (q, *J* = 7.3 Hz, 2H), 3.36 (s, 3H), 2.25 (s, 3H), 1.50 (d, *J* = 7.0 Hz, 3H), 1.40 (t, *J* = 7.3 Hz, 3H). ^13^C NMR (126 MHz, CDCl_3_) δ 169.53, 161.97, 144.04, 137.03, 129.38, 129.00, 127.54, 125.65, 122.16, 121.63 (q, *J*_CF_ = 288.8 Hz, CF_3_),120.68, 105.80, 81.75 (hept, *J*_CF_ = 28.5 Hz, C(CF_3_)_2_), 53.15, 48.35, 46.64, 17.19, 15.49, 14.38. HRMS calcd. for C_20_H_22_F_6_N_4_O_3_ ([M+H]^+^), 481.1674; found, 481.1677.

1-Ethyl-N-(1-((4-(1,1,1,3,3,3-hexafluoro-2-methoxypropan-2-yl)-2-methylphenyl)amino)-1-oxobutan-2-yl)-1H-pyrazole-3-carboxamide I-5. Yellow solid (51% yield), m.p. 136.6–137.6 °C. ^1^H NMR (500 MHz, Chloroform-*d*) δ 8.71 (s, 1H), 8.07 (d, *J* = 8.7 Hz, 1H), 7.33 (d, *J* = 2.3 Hz, 1H), 7.31 (d, *J* = 8.7 Hz, 1H), 7.25 (d, *J* = 7.9 Hz, 2H), 6.70 (s, 1H), 4.63 (q, *J* = 7.8 Hz, 1H), 4.10 (q, *J* = 7.3 Hz, 2H), 3.37 (s, 3H), 2.26 (s, 3H), 2.09 (dq, *J* = 14.2, 7.0 Hz, 1H), 1.82 (dq, *J* = 14.4, 7.5 Hz, 1H), 1.42 (t, *J* = 7.3 Hz, 3H), 1.02 (t, *J* = 7.4 Hz, 3H). ^13^C NMR (126 MHz, CDCl_3_) δ 169.01, 162.01, 144.18, 136.99, 129.28, 128.99, 127.44, 125.68, 122.15, 121.42 (q, *J*_CF_ = 291.1 Hz, CF_3_), 120.69, 105.72, 81.74 (hept, *J*_CF_ = 27.7 Hz, C(CF_3_)_2_), 54.16, 53.15, 46.61, 23.06, 17.20, 14.41, 9.43. HRMS calcd. for C_21_H_24_F_6_N_4_O_3_ ([M+H]^+^), 495.1831; found, 495.1835.

1-Methyl-N-(1-((2-methyl-4-(perfluoropropan-2-yl)phenyl)amino)-1-oxopropan-2-yl)-1H-pyrazole-4-carboxamide II-a-6. White solid (52% yield), m.p. 125.6–126.9 °C. ^1^H NMR (500 MHz, Chloroform-*d*) δ 8.77 (s, 1H), 8.08 (d, *J* = 9.1 Hz, 1H), 7.79 (s, 1H), 7.73 (s, 1H), 7.31 (d, *J* = 7.6 Hz, 2H), 6.37 (d, *J* = 7.5 Hz, 1H), 4.80 (q, *J* = 7.1 Hz, 1H), 3.85 (s, 3H), 2.25 (s, 3H), 1.50 (d, *J* = 7.0 Hz, 3H). ^13^C NMR (126 MHz, CDCl_3_) δ 169.75, 162.25, 137.52, 137.39, 130.88, 127.75, 126.59 (d, *J*_CF_ = 10.1 Hz, Ar-C_3_), 123.13 (d, *J*_CF_ = 10.1 Hz, Ar-C_5_), 121.29 (d, *J*_CF_ = 20.2 Hz, Ar-C_4_), 120.75, 119.54 (qd, ^1^*J*_CF_ = 286 Hz, ^2^*J*_CF_ = 26.5 Hz, CF_3_), 116.48, 90.27 (dsept, ^1^*J*_CF_ = 201.6 Hz, ^2^*J*_CF_ = 32.8 Hz, CF(CF_3_)_2_), 48.50, 38.33, 17.07, 15.71. HRMS calcd. for C_18_H_17_F_7_N_4_O_2_ ([M+H]^+^), 455.1318; found, 455.1320.

N-(1-((2-Methoxy-4-(perfluoropropan-2-yl)phenyl)amino)-1-oxopropan-2-yl)-1-methyl-1H-pyrazole-4-carboxamide II-a-7. Yellow solid (50% yield), m.p. 112.3–113.3 °C. ^1^H NMR (500 MHz, Chloroform-*d*) δ 8.46 (s, 1H), 8.39 (d, *J* = 8.6 Hz, 1H), 7.38 (d, *J* = 2.1 Hz, 1H), 7.13 (d, *J* = 8.7 Hz, 1H), 7.00 (s, 1H), 6.85 (d, *J* = 9.2 Hz, 1H), 6.57 (d, *J* = 2.1 Hz, 1H), 4.75 (m, 1H), 4.10 (s, 3H), 3.85 (s, 3H), 1.51 (d, *J* = 7.0 Hz, 3H). ^13^C NMR (126 MHz, CDCl_3_) δ 169.89, 161.48, 147.19, 137.35, 130.78, 128.81, 120.83 (d, *J*_CF_ = 20.2 Hz, Ar-C_4_), 119.54 (qd, ^1^*J*_CF_ = 288.5 Hz, ^2^*J*_CF_ = 27.7 Hz, CF_3_), 118.66, 117.69 (d, *J*_CF_ = 10.1 Hz, Ar-C_3_), 116.89, 106.35 (d, *J*_CF_ = 12.6 Hz, Ar-C_5_), 90.28 (dsept, ^1^*J*_CF_ = 202.9 Hz, ^2^*J*_CF_ = 32.8 Hz, CF(CF_3_)_2_), 55.06, 48.60, 38.29, 16.92. HRMS calcd. for C_18_H_17_F_7_N_4_O_3_ ([M+Na]^+^), 493.1087; found, 493.1092.

N-(1-((2-Methoxy-4-(perfluoropropan-2-yl)phenyl)amino)-1-oxobutan-2-yl)-1-methyl-1H-pyrazole-4-carboxamide II-a-8. Yellow solid (50% yield), m.p. 136.2–139 °C. ^1^H NMR (500 MHz, Chloroform-*d*) δ 8.64 (s, 1H), 8.46 (d, *J* = 8.6 Hz, 1H), 7.88 (s, 1H), 7.85 (s, 1H), 7.17 (d, *J* = 8.7 Hz, 1H), 7.05 (s, 1H), 6.98 (d, *J* = 7.9 Hz, 1H), 4.73 (q, *J* = 7.4 Hz, 1H), 3.90 (s, 3H), 3.88 (s, 3H),2.01 (m, 1H), 1.80 (m, 1H), 1.01 (t, *J* = 7.4 Hz, 3H). ^13^C NMR (126 MHz, CDCl_3_) δ 169.44, 161.62, 147.19, 137.38, 130.76, 128.68, 120.90 (d, *J*_CF_ = 20.2 Hz, Ar-C_4_), 119.53 (qd, ^1^*J*_CF_ = 287.3 Hz, ^2^*J*_CF_ = 27.7 Hz, CF_3_), 118.69, 117.69 (d, *J*_CF_ = 10.1 Hz, Ar-C_3_), 116.96, 106.34 (d, *J*_CF_ = 12.6 Hz, Ar-C_5_), 90.28 (dsept, ^1^*J*_CF_ = 202.9 Hz, ^2^*J*_CF_ = 34 Hz, CF(CF_3_)_2_), 55.05, 54.24, 38.28, 24.54, 9.05. HRMS calcd. for C_19_H_19_F_7_N_4_O_3_ ([M+H]^+^), 485.1424; found, 485.1426.

N-(1-((4-(1,1,1,3,3,3-Hexafluoro-2-methoxypropan-2-yl)-2-methylphenyl)amino)-1-oxopropan-2-yl)-1-methyl-1H-pyrazole-4-carboxamide II-a-9. White solid (48% yield), m.p. 161.1–161.9 °C. ^1^H NMR (500 MHz, Chloroform-*d*) δ 8.95 (s, 1H), 7.96 (d, *J* = 9.3 Hz, 1H), 7.83 (d, *J* = 3.6 Hz, 2H), 7.28 (d, *J* = 7.3 Hz, 2H), 7.19 (d, *J* = 7.3 Hz, 1H), 4.86 (m, 1H), 3.81 (s, 3H), 3.37 (s, 3H), 2.25 (s, 3H), 1.42 (d, *J* = 7.0 Hz, 3H). ^13^C NMR (126 MHz, CDCl_3_) δ 170.29, 162.29, 137.77, 136.69, 130.97, 129.15, 128.06, 125.60, 122.73, 121.02, 120.38 (q, *J*_CF_ = 378 Hz, CF_3_), 116.65, 81.70 (hept, *J*_CF_ = 27.7 Hz, C(CF_3_)_2_), 53.18, 48.36, 38.26, 17.17, 15.77. HRMS calcd. for C_19_H_20_F_6_N_4_O_3_ ([M+H]^+^), 467.1518; found, 467.1521.

N-(1-((4-(1,1,1,3,3,3-Hexafluoro-2-methoxypropan-2-yl)-2-methylphenyl)amino)-1-oxobutan-2-yl)-1-methyl-1H-pyrazole-4-carboxamide II-a-10. White solid (51% yield), m.p. 174.1–174.6 °C. ^1^H NMR (500 MHz, Chloroform-*d*) δ 8.88 (s, 1H), 8.02 (d, *J* = 8.4 Hz, 1H), 7.91 (d, *J* = 6.6 Hz, 2H), 7.37 (d, *J* = 10.1 Hz, 2H), 7.22 (d, *J* = 8.0 Hz, 1H), 4.76 (q, *J* = 7.4 Hz, 1H), 3.90 (s, 3H), 3.46 (s, 3H), 2.33 (s, 3H), 2.05 (dq, *J* = 14.1, 7.2 Hz, 1H), 1.81 (dq, *J* = 14.1, 7.4 Hz, 1H), 1.01 (t, *J* = 7.4 Hz, 3H). ^13^C NMR (126 MHz, CDCl_3_) δ 169.91, 162.37, 140.66, 137.75, 136.52, 130.91, 129.17, 125.59, 122.91, 122.38 (q, *J*_CF_ = 390.6 Hz, CF_3_), 121.32, 116.66, 81.69 (hept, *J*_CF_ = 27.7 Hz, C(CF_3_)_2_), 54.25, 53.20, 38.27, 23.49, 17.20, 9.30. HRMS calcd. for C_20_H_22_F_6_N_4_O_3_ ([M+H]^+^), 481.1630; found, 481.1680.

1,3-Dimethyl-N-(1-((2-methyl-4-(perfluoropropan-2-yl)phenyl)amino)-1-oxopropan-2-yl)-1H-pyrazole-4-carboxamide II-a-11. Yellow solid (58% yield), m.p. 107–108 °C. ^1^H NMR (500 MHz, DMSO-*d*_6_) δ 9.58 (s, 1H), 8.23 (s, 1H), 8.11 (d, *J* = 7.0 Hz, 1H), 7.87 (d, *J* = 8.5 Hz, 1H), 7.50 (s, 1H), 7.47 (d, *J* = 8.7 Hz, 1H), 4.67 (m, 1H), 3.79 (s, 3H), 2.33 (d, *J* = 4.5 Hz, 6H), 1.42 (d, *J* = 7.2 Hz, 3H). ^13^C NMR (126 MHz, DMSO) δ 172.42, 163.62, 149.09, 139.98, 132.83, 132.21, 127.53 (d, *J*_CF_ = 11.3 Hz, Ar-C_3_), 124.71, 123.79 (d, *J*_CF_ = 11.3 Hz, Ar-C_5_), 121.15 (d, *J*_CF_ = 21.4 Hz, Ar-C_4_), 120.78 (qd, ^1^*J*_CF_ = 287.3 Hz, ^2^*J*_CF_ = 27.7 Hz, CF_3_), 114.32, 91.59 (dsept, ^1^*J*_CF_ = 200.3 Hz, ^2^*J*_CF_ = 32.8 Hz, CF(CF_3_)_2_), 49.36, 38.95, 18.25, 17.96, 13.59. HRMS calcd. for C_19_H_19_F_7_N_4_O_2_ ([M+Na]^+^), 491.1294; found, 491.1299. 

N-(1-((2-Methoxy-4-(perfluoropropan-2-yl)phenyl)amino)-1-oxopropan-2-yl)-1,3-dimethyl-1H-pyrazole-4-carboxamide II-a-12. Yellow solid (46% yield), m.p. 84.4–85.4 °C. ^1^H NMR (500 MHz, Chloroform-*d*) δ 8.68 (s, 1H), 8.45 (d, *J* = 8.6 Hz, 1H), 7.75 (s, 1H), 7.18 (d, *J* = 10.5 Hz, 1H), 7.05 (s, 1H), 6.50 (d, *J* = 7.4 Hz, 1H), 4.83 (m, 1H), 3.91 (s, 3H), 3.81 (s, 3H), 2.48 (s, 3H), 1.53 (d, *J* = 7.0 Hz, 3H). ^13^C NMR (126 MHz, CDCl_3_) δ 170.16, 162.52, 147.85, 147.19, 131.21, 128.81, 120.83 (d, *J*_CF_ = 20.2 Hz, Ar-C_4_), 119.53 (qd, ^1^*J*_CF_ = 287.3 Hz, ^2^*J*_CF_ = 27.7 Hz, CF_3_), 118.66, 117.66 (d, *J*_CF_ = 10.1 Hz, Ar-C_3_), 113.81, 106.34 (d, *J*_CF_ = 11.3 Hz, Ar-C_5_), 90.27 (dsept, ^1^*J*_CF_ = 201.6 Hz, ^2^*J*_CF_ = 32.8 Hz, CF(CF_3_)_2_), 55.01, 48.62, 37.87, 17.12, 12.51. HRMS calcd. for C_19_H_19_F_7_N_4_O_3_ ([M+H]^+^), 485.1424; found, 485.1423. 

N-(1-((2-Methoxy-4-(perfluoropropan-2-yl)phenyl)amino)-1-oxobutan-2-yl)-1,3-dimethyl-1H-pyrazole-4-carboxamide II-a-13. White solid (47% yield), m.p. 161.9–163.1 °C. ^1^H NMR (500 MHz, Chloroform-*d*) δ 8.49 (s, 1H), 8.39 (d, *J* = 8.6 Hz, 1H), 7.71 (s, 1H), 7.12 (d, *J* = 8.7 Hz, 1H), 6.99 (s, 1H), 6.48 (d, *J* = 7.3 Hz, 1H), 4.66 (q, *J* = 6.9 Hz, 1H), 3.85 (s, 3H), 3.74 (s, 3H), 2.42 (s, 3H), 1.98 (dp, *J* = 14.0, 7.3 Hz, 1H), 1.75 (dq, *J* = 14.2, 7.3 Hz, 1H), 0.95 (t, *J* = 7.4 Hz, 3H). ^13^C NMR (126 MHz, CDCl_3_) δ 169.51, 162.58, 147.64, 147.17, 131.27, 128.71, 120.89 (d, *J*_CF_ = 21.4 Hz, Ar-C_4_), 119.54 (qd, ^1^*J*_CF_ = 288.5 Hz, ^2^*J*_CF_ = 29 Hz, CF_3_), 118.68, 117.69 (d, *J*_CF_ = 10.1 Hz, Ar-C_3_), 114.01, 106.35 (d, *J*_CF_ = 12.6 Hz, Ar-C_5_), 90.28 (dsept, ^1^*J*_CF_ = 201.6 Hz, ^2^*J*_CF_ = 32.8 Hz, CF(CF_3_)_2_), 55.02, 54.12, 37.88, 24.65, 12.58, 8.95. HRMS calcd. for C_20_H_21_F_7_N_4_O_3_ ([M+H]^+^), 499.1580; found, 499.1582. 

N-(1-((4-(1,1,1,3,3,3-Hexafluoro-2-methoxypropan-2-yl)-2-methylphenyl)amino)-1-oxopropan-2-yl)-1,3-dimethyl-1H-pyrazole-4-carboxamide II-a-14. White solid (45% yield), m.p. 169.2–171.3 °C. ^1^H NMR (500 MHz, Chloroform-*d*) δ 8.99 (s, 1H), 7.97 (d, *J* = 9.2 Hz, 1H), 7.73 (s, 1H), 7.29 (d, *J* = 7.8 Hz, 2H), 6.73 (d, *J* = 7.5 Hz, 1H), 4.83 (m, 1H), 3.72 (s, 3H), 3.38 (s, 3H), 2.40 (s, 3H), 2.26 (s, 3H), 1.43 (d, *J* = 7.0 Hz, 3H). ^13^C NMR (126 MHz, CDCl_3_) δ 170.22, 163.22, 148.18, 136.80, 131.19, 129.15, 125.61, 122.64, 121.39 (q, *J*_CF_ = 291 Hz, CF_3_), 120.94, 120.91, 113.44, 81.71 (hept, *J*_CF_ = 15.2 Hz, C(CF_3_)_2_), 53.18, 48.29, 37.90, 17.20, 15.94, 12.55. HRMS calcd. for C_20_H_22_F_6_N_4_O_3_ ([M+H]^+^), 481.1674; found, 481.1677. 

N-(1-((4-(1,1,1,3,3,3-Hexafluoro-2-methoxypropan-2-yl)-2-methylphenyl)amino)-1-oxobutan-2-yl)-1,3-dimethyl-1H-pyrazole-4-carboxamide II-a-15. White solid (48% yield), m.p. 125.3–126.3 °C. ^1^H NMR (500 MHz, Chloroform-*d*) δ 8.94 (s, 1H), 7.93 (d, *J* = 9.0 Hz, 1H), 7.74 (s, 1H), 7.29 (d, *J* = 7.8 Hz, 2H), 6.88 (d, *J* = 7.8 Hz, 1H), 4.69 (q, *J* = 7.5 Hz, 1H), 3.70 (s, 3H), 3.38 (s, 3H), 2.39 (s, 3H), 2.24 (s, 3H), 1.96 (dq, *J* = 14.0, 7.1 Hz, 1H), 1.73 (dq, *J* = 14.2, 7.4 Hz, 1H), 0.93 (t, *J* = 7.4 Hz, 3H). ^13^C NMR (126 MHz, CDCl_3_) δ 169.88, 163.31, 148.12, 136.70, 131.16, 129.17, 128.19, 125.58, 122.79, 121.38 (q, *J*_CF_ = 291.1 Hz, CF_3_), 121.21, 113.57, 81.70 (hept, *J*_CF_ = 27.7 Hz, C(CF_3_)_2_), 54.06, 53.18, 37.87, 23.57, 17.21, 12.57, 9.23. HRMS calcd. for C_21_H_24_F_6_N_4_O_3_ ([M+H]^+^), 495.1831; found, 492.1834. 

N-(1-((4-(1,1,1,3,3,3-Hexafluoro-2-methoxypropan-2-yl)-2-methylphenyl)amino)-1-oxopropan-2-yl)-1-methyl-3-(trifluoromethyl)-1H-pyrazole-4-carboxamide II-a-16. White solid (45% yield), m.p. 171.6–174.4 °C. ^1^H NMR (500 MHz, Chloroform-*d*) δ 8.55 (s, 1H), 8.01 (d, *J* = 8.6 Hz, 1H), 7.90 (s, 1H), 7.31 (d, *J* = 8.7 Hz, 1H), 7.28 (s, 1H), 6.61 (d, *J* = 6.5 Hz, 1H), 4.76 (m, 1H), 3.88 (s, 3H), 3.39 (s, 3H), 2.24 (s, 3H), 1.49 (d, *J* = 7.0 Hz, 3H). ^13^C NMR (126 MHz, CDCl_3_) δ 169.00, 160.18, 137.94 (q, *J*_CF_ = 37.8 Hz, pyrazole-C_3_), 136.69, 134.21, 129.09, 127.44, 125.78, 122.57, 120.72, 119.48 (q, *J*_CF_ = 192.8 Hz, CF_3_), 114.93, 81.72 (hept, *J*_CF_ = 30.0 Hz, C(CF_3_)_2_), 53.20, 49.04, 38.85, 17.05, 15.60. HRMS calcd. for C_20_H_19_F_9_N_4_O_3_ ([M+Na]^+^), 557.1211; found, 557.1216. 

3-(Difluoromethyl)-N-(1-((4-(1,1,1,3,3,3-hexafluoro-2-methoxypropan-2-yl)-2-methylphenyl)amino)-1-oxopropan-2-yl)-1-methyl-1H-pyrazole-4-carboxamide II-a-17. White solid (45% yield), m.p. 137.9–139.1 °C. ^1^H NMR (500 MHz, Chloroform-*d*) δ 8.66 (s, 1H), 8.07 (d, *J* = 8.7 Hz, 1H), 7.90 (s, 1H), 7.32 (d, *J* = 8.7 Hz, 1H), 7.27 (s, 1H), 6.90 (s, 0.25H), 6.78 (d, *J* = 12.7 Hz, 1.5H), 6.68 (s, 0.25H), 4.73 (m, 1H), 3.86 (s, 3H), 3.38 (s, 3H), 2.24 (s, 3H), 1.48 (d, *J* = 7.0 Hz, 3H). ^13^C NMR (126 MHz, CDCl_3_) δ 169.10, 161.00, 142.17 (t, *J*_CF_ = 27.7 Hz, pyrazole-C_3_), 136.87, 134.33, 129.02, 127.21, 125.79, 122.30, 121.42 (q, *J*_CF_ = 291.1 Hz, CF_3_), 120.55, 114.46, 110.61 (t, *J*_CF_ = 252 Hz, CF_2_), 81.74 (hept, *J*_CF_ = 27.7 Hz, C(CF_3_)_2_), 53.18, 48.97, 38.58, 17.07, 15.29. HRMS calcd. for C_20_H_20_F_8_N_4_O_3_ ([M+H]^+^), 517.1486; found, 517.1530. 

3-(Difluoromethyl)-N-((RS)-1-(((RS)-1-(4-fluorophenyl)ethyl)amino)-1-oxopropan-2-yl)-1-methyl-1H-pyrazole-4-carboxamide II-b-18 diastereomixture. White solid (46% yield), m.p. 149.9–152.7 °C. ^1^H NMR (500 MHz, Chloroform-*d*) δ 7.81 (d, *J* = 9.3 Hz, 1H), 7.20–7.15 (m, 1H), 7.12 (m, 2H), 7.08–6.99 (m, 1.25H), 6.95–6.88 (m, 1.5H), 6.87–6.81 (m, 1H), 6.80 (m, 0.25H), 4.93 (m, 1H), 4.56 (m, 1H), 3.81 (d, *J* = 9.5 Hz, 3H), 1.42–1.20 (m, 6H). ^13^C NMR (126 MHz, CDCl_3_) δ 170.34, 160.90 (d, *J*_CF_ = 245.7 Hz, Ar-C_4_), 160.58, 143.29 (t, *J*_CF_ = 26.5 Hz, pyrazole-C_3_), 138.01, 132.74, 126.51 (d, *J*_CF_ = 10.1 Hz, Ar-C_2,6_), 114.77, 114.30 (d, *J*_CF_ = 21.4 Hz, Ar-C_3,5_), 109.82 (t, *J*_CF_ = 235.6 Hz, CF_2_), 47.94, 47.47, 38.51, 20.99, 16.26. HRMS calcd. for C_17_H_19_F_3_N_4_O_2_ ([M+Na]^+^), 391.1358; found, 391.1363. 

N-(1-((1-(4-Fluorophenyl)ethyl)amino)-1-oxopropan-2-yl)-1-methyl-3-(trifluoromethyl)-1H-pyrazole-4-carboxamide II-b-19 diastereomixture. White solid (44% yield), m.p. 135.2–138.3 °C. ^1^H NMR (500 MHz, Chloroform-*d*) δ 7.84–7.76 (m, 1H), 7.22–7.16 (m, 1H), 7.16–7.11 (m, 1H), 7.06–6.88 (m, 3H), 6.88–6.81 (m, 1H), 4.93 (m, 1H), 4.56 (m, 1H), 3.85 (d, *J* = 11.6 Hz, 3H), 1.37 (dd, *J* = 16.4, 6.9 Hz, 3H), 1.25 (dd, *J* = 15.9, 6.9 Hz, 3H). ^13^C NMR (126 MHz, CDCl_3_) δ 170.17, 160.92 (d, *J*_CF_ = 247 Hz, Ar-C_4_), 159.58, 138.50 (q, *J*_CF_ = 38 Hz, pyrazole-C_3_), 137.90, 133.09, 126.60 (d, *J*_CF_ = 7.6 Hz, Ar-C_2,6_), 119.76 (q, *J*_CF_ = 270.3 Hz, CF_3_), 115.42, 114.47 (d, *J*_CF_ = 34 Hz, Ar-C_3,5_), 48.02, 47.47, 38.71, 20.92, 16.91. HRMS calcd. for C_17_H_18_F_4_N_4_O_2_ ([M+H]^+^), 387.1444; found, 387.1442.

3-(Difluoromethyl)-N-(1-((1-(4-fluorophenyl)ethyl)amino)-1-oxobutan-2-yl)-1-methyl-1H-pyrazole-4-carboxamide II-b-20 diastereomixture. White solid (46% yield), m.p. 154.2–157.8 °C. ^1^H NMR (500 MHz, Chloroform-*d*) δ 7.82 (d, *J* = 11.9 Hz, 1H), 7.21–7.16 (m, 1H), 7.16–7.11 (m, 1H), 7.05 (m, 1H), 7.01 (m, 0.25H), 7.00–6.94 (m, 1H), 6.93 (m, 0.5H), 6.92–6.82 (m, 2H), 6.78 (m, 0.25H), 5.02–4.89 (m, 1H), 4.43 (m, 1H), 3.82 (d, *J* = 9.6 Hz, 3H), 1.79–1.68 (m, 1H), 1.57 (m, 1H), 1.37 (dd, *J* = 17.7, 7.0 Hz, 3H), 0.80 (dt, *J* = 35.6, 7.4 Hz, 3H). ^13^C NMR (126 MHz, CDCl_3_) δ 169.56, 160.85 (d, *J*_CF_ = 245.7 Hz, Ar-C_4_), 160.64, 143.12 (t, *J*_CF_ = 27 Hz, pyrazole-C_3_), 138.04, 132.90, 126.58 (d, *J*_CF_ = 12.6 Hz, Ar-C_2,6_), 114.90, 114.30 (d, *J*_CF_ = 22.7 Hz, Ar-C_3,5_), 109.96 (t, *J*_CF_ = 234.4 Hz, CF_2_), 53.55, 47.44, 38.48, 23.92, 21.27, 8.84. HRMS calcd. for C_18_H_21_F_3_N_4_O_2_ ([M+H]^+^), 383.1695; found, 383.1697.

N-(1-((1-(4-Fluorophenyl)ethyl)amino)-1-oxobutan-2-yl)-1-methyl-3-(trifluoromethyl)-1H-pyrazole-4-carboxamide II-b-21 diastereomixture. White solid (46% yield), m.p. 165.6–170.3 °C. ^1^H NMR (500 MHz, Chloroform-*d*) δ 7.82 (d, *J* = 10.6 Hz, 1H), 7.20–7.16 (m, 1H), 7.16–6.96 (m, 3H), 6.95–6.89 (m, 1H), 6.87–6.82 (m, 1H), 4.94 (m, 1H), 4.51–4.38 (m, 1H), 3.82 (d, *J* = 13.9 Hz, 3H), 1.75–1.64 (m, 1H), 1.61–1.46 (m, 1H), 1.36 (dd, *J* = 23.3, 6.9 Hz, 3H), 0.78 (dt, *J* = 48.2, 7.4 Hz, 3H). ^13^C NMR (126 MHz, CDCl_3_) δ 169.62, 160.92 (d, *J*_CF_ = 245.7 Hz, Ar-C_4_), 159.61, 138.55 (q, *J*_CF_ = 37.5 Hz, pyrazole-C_3_), 138.05, 133.21, 126.59 (d, *J*_CF_ = 10.8 Hz, Ar-C_3,5_), 119.80 (q, *J*_CF_ = 264.6 Hz, CF_3_), 115.49, 114.37 (d, *J*_CF_ = 20.2 Hz, Ar-C_3,5_), 53.61, 47.46, 38.70, 24.55, 20.84, 8.81. HRMS calcd. for C_18_H_20_F_4_N_4_O_2_ ([M+H]^+^), 401.1601; found, 401.1600.

3-(Difluoromethyl)-N-(1-((4-fluorophenethyl)amino)-1-oxopropan-2-yl)-1-methyl-1H-pyrazole-4-carboxamide II-c-22. White solid (48% yield), m.p. 115.5–119.8 °C. ^1^H NMR (500 MHz, Chloroform-*d*) δ 7.80 (s, 1H), 7.07–7.00 (m, 2H), 6.95 (s, 0.25H), 6.92–6.78 (m, 3.5H), 6.73 (s, 0.25H), 6.58 (t, *J* = 5.9 Hz, 1H), 4.50 (m, 1H), 3.84 (s, 3H), 3.40 (m, 2H), 2.70 (t, *J* = 7.1 Hz, 2H), 1.32 (d, *J* = 7.1 Hz, 3H). ^13^C NMR (126 MHz, CDCl_3_) δ 171.29, 160.55 (d, *J*_CF_ = 244.4 Hz, Ar-C_4_), 160.22, 142.83 (t, *J*_CF_ = 26.5 Hz, pyrazole-C_3_), 133.36, 133.11, 129.14 (d, *J*_CF_ = 7.6 Hz, Ar-C_2,6_), 114.93, 114.24 (d, *J*_CF_ = 21.4 Hz, Ar-C_3,5_), 110.05 (t, *J*_CF_ = 192.8 Hz, CF_2_), 48.05, 39.71, 38.50, 33.70, 17.00. HRMS calcd. for C_17_H_19_F_3_N_4_O_2_ ([M+H]^+^), 369.1538; found, 369.1537.

N-(1-((4-Fluorophenethyl)amino)-1-oxopropan-2-yl)-1-methyl-3-(trifluoromethyl)-1H-pyrazole-4-carboxamide II-c-23. White solid (46% yield), m.p. 144.3–148.9 °C. ^1^H NMR (500 MHz, Chloroform-*d*) δ 7.87 (d, *J* = 4.7 Hz, 1H), 7.28 (m, 1H), 7.15 (m, 1H), 7.03 (m, 2H), 6.83 (m, 2H), 4.62 (m, 1H), 3.81 (d, *J* = 4.6 Hz, 3H), 3.42 (m, 1H), 3.35–3.23 (m, 1H), 2.68 (t, *J* = 7.3 Hz, 2H), 1.30 (d, *J* = 5.4 Hz, 3H). ^13^C NMR (126 MHz, CDCl_3_) δ 171.72, 160.53 (d, *J*_CF_ = 244.4 Hz, Ar-C_4_), 159.31, 138.77 (q, *J*_CF_ = 26.5 Hz, pyrazole-C_3_),133.38, 132.98, 129.13 (d, *J*_CF_ = 7.6 Hz, Ar-C_2,6_), 119.78 (q, *J*_CF_ = 270.9 Hz, CF_3_), 115.20, 114.21 (d, *J*_CF_ = 21.4 Hz, Ar-C_3,5_), 48.13, 39.82, 38.66, 33.75, 17.58. HRMS calcd. for C_17_H_18_F_4_N_4_O_2_ ([M+H]^+^), 387.1444; found, 387.1442.

3-(Difluoromethyl)-N-(1-((4-fluorophenethyl)amino)-1-oxobutan-2-yl)-1-methyl-1H-pyrazole-4-carboxamide II-c-24. White solid (45% yield), m.p. 121.7–124.6 °C. ^1^H NMR (500 MHz, Chloroform-*d*) δ 7.83 (s, 1H), 7.04 (m, 2H), 6.97 (s, 0.25H), 6.94 (d, *J* = 7.5 Hz, 1H), 6.84 (m, 2.5H), 6.75 (s, 0.25H), 6.59 (t, *J* = 5.6 Hz, 1H), 4.39 (q, *J* = 7.4 Hz, 1H), 3.85 (s, 3H), 3.41 (q, *J* = 7.2 Hz, 2H), 2.70 (t, *J* = 7.1 Hz, 2H), 1.80 (dp, *J* = 13.6, 7.4 Hz, 1H), 1.60 (dp, *J* = 14.7, 7.4 Hz, 1H), 0.83 (t, *J* = 7.4 Hz, 3H). ^13^C NMR (126 MHz, CDCl_3_) δ 170.52, 160.55 (d, *J*_CF_ = 245.7 Hz, Ar-C_4_), 160.37, 142.82 (t, *J*_CF_ = 27.7 Hz, pyrazole-C_3_), 133.31, 133.15, 129.11 (d, *J*_CF_ = 7.9 Hz, Ar-C_2,6_), 114.97, 114.26 (d, *J*_CF_ = 21.6 Hz, Ar-C_3,5_), 110.08 (t, *J*_CF_ = 234.4 Hz, CF_2_), 53.56, 39.66, 38.52, 33.74, 24.20, 8.80. HRMS calcd. for C_18_H_21_F_3_N_4_O_2_ ([M+H]^+^), 383.1695; found, 383.1697.

N-(1-((4-Fluorophenethyl)amino)-1-oxobutan-2-yl)-1-methyl-3-(trifluoromethyl)-1H-pyrazole-4-carboxamide II-c-25. White solid (47% yield), m.p. 152.8–155.1 °C. ^1^H NMR (500 MHz, Chloroform-*d*) δ 7.81 (s, 1H), 7.06 (dd, *J* = 8.5, 5.4 Hz, 2H), 6.86 (t, *J* = 8.7 Hz, 2H), 6.64 (d, *J* = 7.4 Hz, 1H), 6.35 (t, *J* = 5.5 Hz, 1H), 4.39 (q, *J* = 6.9 Hz, 1H), 3.89 (s, 3H), 3.49–3.39 (m, 2H), 2.72 (t, *J* = 7.1 Hz, 2H), 1.82 (m, 1H), 1.57 (m, 1H), 0.83 (t, *J* = 7.4 Hz, 3H). ^13^C NMR (126 MHz, CDCl_3_) δ 170.22, 160.59 (d, *J*_CF_ = 244.7 Hz, Ar-C_4_), 159.36, 138.13 (q, *J*_CF_ = 27.5 Hz, pyrazole-C_3_), 133.53, 133.25, 129.13 (d, *J*_CF_ = 7.9 Hz, Ar-C_2,6_), 119.8 (q, *J*_CF_ = 269.6 Hz, CF_3_), 115.54, 114.31 (d, *J*_CF_ = 21.5 Hz, Ar-C_3,5_), 53.54, 39.68, 38.77, 33.76, 24.51, 8.61. HRMS calcd. for C_18_H_20_F_4_N_4_O_2_ ([M+H]^+^), 401.1601; found, 401.1601.

1-Ethyl-3-methyl-N-(1-((2-methyl-4-(perfluoropropan-2-yl)phenyl)amino)-1-oxopropan-2-yl)-1H-pyrazole-5-carboxamide III-26. White solid (48% yield), m.p. 143.2–145.1 °C. ^1^H NMR (500 MHz, Chloroform-*d*) δ 8.56 (s, 1H), 8.01 (d, *J* = 8.9 Hz, 1H), 7.31 (d, *J* = 6.8 Hz, 2H), 6.75 (d, *J* = 7.3 Hz, 1H), 6.31 (s, 1H), 4.77 (m, 1H), 4.42 (q, *J* = 7.1 Hz, 2H), 2.23 (s, 3H), 2.16 (s, 3H), 1.51 (d, *J* = 6.9 Hz, 3H), 1.31 (t, *J* = 7.1 Hz, 3H). ^13^C NMR (126 MHz, CDCl_3_) δ 169.28, 159.56, 146.13, 137.31, 132.79, 127.71, 126.68 (d, *J*_CF_ = 10.1 Hz, Ar-C_3_), 123.29 (d, *J*_CF_ = 10.1 Hz, Ar-C_5_), 121.61 (d, *J*_CF_ = 20.2 Hz, Ar-C_4_), 120.90, 119.55 (qd, ^1^*J*_CF_ = 287.3 Hz, ^2^*J*_CF_ = 27.7 Hz, CF_3_), 105.61, 90.28 (dsept, ^1^*J*_CF_ = 201.6 Hz, ^2^*J*_CF_ = 32.8 Hz, CF(CF_3_)_2_), 48.68, 45.50, 16.99, 15.90, 14.98, 12.24. HRMS calcd. for C_20_H_21_F_7_N_4_O_2_ ([M+H]^+^), 483.1631; found, 483.1627.

1-Ethyl-N-(1-((2-methoxy-4-(perfluoropropan-2-yl)phenyl)amino)-1-oxopropan-2-yl)-3-methyl-1H-pyrazole-5-carboxamide III -27. Yellow solid (47% yield), m.p. 108.7–110.2 °C. ^1^H NMR (500 MHz, Chloroform-*d*) δ 8.55 (s, 1H), 8.46 (d, *J* = 8.6 Hz, 1H), 7.20 (d, *J* = 8.7 Hz, 1H), 7.06 (s, 1H), 6.72 (d, *J* = 7.5 Hz, 1H), 6.38 (s, 1H), 4.80 (m, 1H), 4.52 (q, *J* = 7.1 Hz, 2H), 3.92 (s, 3H), 2.25 (s, 3H), 1.56 (d, *J* = 7.0 Hz, 3H), 1.41 (t, *J* = 7.2 Hz, 3H). ^13^C NMR (126 MHz, CDCl_3_) δ 169.47, 158.81, 147.11, 146.07, 133.30, 128.64, 121.01 (d, *J*_CF_ = 20.2 Hz, Ar-C_4_), 119.53 (qd, ^1^*J*_CF_ = 287.3 Hz, ^2^*J*_CF_ = 27.7 Hz, CF_3_), 118.70, 117.77 (d, *J*_CF_ = 8.8 Hz, Ar-C_3_), 106.37 (d, *J*_CF_ = 12.6 Hz, Ar-C_5_), 105.43, 90.28 (dsept, ^1^*J*_CF_ = 202.9 Hz, ^2^*J*_CF_ = 32.8 Hz, CF(CF_3_)_2_), 54.99, 48.79, 45.44, 16.98, 14.92, 12.18. HRMS calcd. for C_20_H_21_F_7_N_4_O_3_ ([M+H]^+^), 499.1580; found, 499.1583.

1-Ethyl-N-(1-((2-methoxy-4-(perfluoropropan-2-yl)phenyl)amino)-1-oxobutan-2-yl)-3-methyl-1H-pyrazole-5-carboxamide III -28. White solid (49% yield), m.p. 149.4–151.1 °C. ^1^H NMR (500 MHz, Chloroform-*d*) δ 8.48 (d, *J* = 8.6 Hz, 1H), 8.43 (s, 1H), 7.20 (d, *J* = 8.6 Hz, 1H), 7.06 (s, 1H), 6.71 (d, *J* = 7.9 Hz, 1H), 6.40 (s, 1H), 4.66 (q, *J* = 7.2 Hz, 1H), 4.51 (q, *J* = 7.2 Hz, 2H), 3.93 (s, 3H), 2.26 (s, 3H), 2.07 (dq, *J* = 13.9, 7.0 Hz, 1H), 1.82 (dq, *J* = 14.5, 7.3 Hz, 1H), 1.41 (t, *J* = 7.2 Hz, 3H), 1.04 (t, *J* = 7.4 Hz, 3H). ^13^C NMR (126 MHz, CDCl_3_) δ 168.88, 159.04, 147.09, 145.97, 133.32, 128.61, 120.99 (d, *J*_CF_ = 20.2 Hz, Ar-C_4_), 119.54 (qd, ^1^*J*_CF_ = 287.3 Hz, ^2^*J*_CF_ = 27.7 Hz, CF_3_), 118.68, 117.77 (d, *J*_CF_ = 10.1 Hz, Ar-C_3_), 106.32 (d, *J*_CF_ = 12.6 Hz, Ar-C_5_), 105.34, 90.28 (dsept, ^1^*J*_CF_ = 201.6 Hz, ^2^*J*_CF_ = 32.8 Hz, CF(CF_3_)_2_), 55.00, 45.43, 32.90, 24.54, 14.94, 12.28, 9.01. HRMS calcd. for C_21_H_23_F_7_N_4_O_3_ ([M+H]^+^), 513.1737; found, 513.1740.

1-Ethyl-N-(1-((4-(1,1,1,3,3,3-hexafluoro-2-methoxypropan-2-yl)-2-methylphenyl)amino)-1-oxopropan-2-yl)-3-methyl-1H-pyrazole-5-carboxamide III-29. White solid (46% yield), m.p. 177.8–179.5 °C. ^1^H NMR (500 MHz, DMSO-d_6_) δ 9.55 (s, 1H), 8.61 (d, J = 6.9 Hz, 1H), 7.71 (d, J = 8.4 Hz, 1H), 7.38 (d, J = 8.3 Hz, 2H), 6.76 (s, 1H), 4.65 (m, 1H), 4.41 (q, J = 7.1 Hz, 2H), 3.45 (s, 3H), 2.31 (s, 3H), 2.18 (s, 3H), 1.45 (d, J = 7.2 Hz, 3H), 1.27 (t, J = 7.1 Hz, 3H). ^13^C NMR (126 MHz, DMSO) δ 171.85, 160.04, 145.86, 138.94, 135.16, 132.42, 130.02, 126.23, 125.16, 122.97, 122.75 (q, *J*_CF_ = 291.1 Hz, CF_3_), 107.68, 82.79 (hept, *J*_CF_ = 27.7 Hz, C(CF_3_)_2_), 54.70, 49.71, 45.89, 18.44, 17.93, 16.35, 13.61. HRMS calcd. for C_21_H_24_F_6_N_4_O_3_ ([M+H]^+^), 495.1831; found, 495.1833.

1-Ethyl-N-(1-((4-(1,1,1,3,3,3-hexafluoro-2-methoxypropan-2-yl)-2-methylphenyl)amino)-1-oxobutan-2-yl)-3-methyl-1H-pyrazole-5-carboxamide III-30. White solid (43% yield), m.p. 149.5–153.4 °C. ^1^H NMR (500 MHz, Chloroform-*d*) δ 8.50 (s, 1H), 7.95 (d, *J* = 8.6 Hz, 1H), 7.31 (d, *J* = 9.1 Hz, 1H), 7.29 (s, 1H), 7.04 (d, *J* = 7.9 Hz, 1H), 6.36 (s, 1H), 4.62 (q, *J* = 7.4 Hz, 1H), 4.43 (q, *J* = 7.2 Hz, 2H), 3.38 (s, 3H), 2.24 (s, 3H), 2.15 (s, 3H), 1.98 (dq, *J* = 14.1, 7.2 Hz, 1H), 1.76 (dq, *J* = 14.2, 7.4 Hz, 1H), 1.31 (t, *J* = 7.2 Hz, 3H), 0.95 (t, *J* = 7.4 Hz, 3H). ^13^C NMR (126 MHz, CDCl_3_) δ 169.09, 159.66, 146.07, 136.40, 132.98, 129.21, 127.99, 125.73, 123.08, 121.36 (q, *J*_CF_ = 291.1 Hz, CF_3_), 121.23, 105.67, 81.68 (hept, *J*_CF_ = 27.7 Hz, C(CF_3_)_2_), 54.39, 53.20, 45.45, 23.60, 17.09, 14.99, 12.25, 9.21. HRMS calcd. for C_22_H_26_F_6_N_4_O_3_ ([M+H]^+^), 509.1987; found, 509.1991.

### 3.3. Biological Assay

#### 3.3.1. Insecticidal and Acaricidal Activity

The insecticidal and acaricidal activities were tested against *Aphis craccivora*, *Plutella xylostella*, and *Tetranychus cinnabarinus* reared in a greenhouse. Assessments were made on a dead/alive basis, and mortality rates were corrected using Abbott’s formula [42]. The evaluation was based on a percentage scale of 0–100, where 0 = no activity and 100 = total kill. For comparative purposes, imidacloprid, fenpyroximate, flubendiamide, and clean water were used as controls under the same conditions.

The insecticidal activities of the target compounds against *Aphis craccivora* were evaluated using a previously reported procedure [43]. Leaves from a soybean plant with 20 apterous adults were dipped in the test solution for 5 s, and the excess solution was removed by blotting with filter paper. Mortality rates were evaluated 36 h after treatment. Each treatment was repeated in triplicate. 

The insecticidal activities of the target compounds and flubendiamide against *Plutella xylostella* were evaluated using a previously reported procedure [44]. Fresh cabbage discs were dipped into the prepared solutions containing the compounds for 10 s, air-dried, and placed in a petri dish lined with wet filter paper. The third instar larvae of *Plutella xylostella* were then carefully transferred to the Petri dish. The percentage mortality was evaluated 48 h after treatment. Each treatment was replicated in triplicate.

The acaricidal activity of the target compounds against *Tetranychus cinnabarinus* was tested using a previously reported procedure [45]. Sieva bean plants (*Phaseolus vulgaris*) with primary leaves expanded to 10 cm were selected and thinned out to one plant per pot. A small piece was cut from a leaf taken from the main colony and placed on each leaf of the test plant. This was performed approximately 2 h before treatment to allow the mites to move to the test plant. The size of the leaf pieces from the main colony was varied to obtain approximately 30 mites per leaf. At the time of treatment, the leaf pieces used to transfer mites were removed and discarded. The mite-infested plants were dipped in the test solution for 3 s with agitation and then set in a fume hood to dry. Plants were maintained for 48 h before the numbers of live and dead adults were counted. Each treatment was repeated in triplicate. 

The insecticidal and acaricidal activities from the above tests are summarized in Table 1.

#### 3.3.2. Fungicidal Activity

The fungicidal activities of the synthesized compounds against *Rhizoctonia solani*, *Cytospora* sp., *Botrytis cinerea*, *Fusarium graminearum*, *Pythium aphanidermatum*, and *Sclerotinia sclerotiorum* were evaluated in vitro at 50 mg/L for preliminary screening according to previously reported procedures [46]. The commercial SDHI fungicide fluxapyroxad was used as a positive control. Compounds with good inhibitory activities were further evaluated for their median effective concentration (EC_50_) values determined by established procedures [47]. A blank control was maintained with 0.5% DMSO (*v*/*v*) mixed with potato dextrose agar medium (PDA) (the same amount of DMSO was added to the sterile medium as a blank control). The mycelial disks (5 mm) of phytopathogenic fungi were inoculated onto PDA plates and incubated at 25 °C in the dark. Each sample was measured in triplicate and the diameters (mm) of the inhibition zones were measured using the cross-bracketing method. Growth inhibition rates were calculated when the blank control hyphae grew to the edge of the Petri dish, according to the following formula:

Mycelial growth inhibition (%) = [(dc − dt)/(dc − 5 mm)] × 100(1)

where dc and dt are the average diameters of the fungal colonies in the black control and treatment, respectively. The fungicidal activities observed in the above tests are summarized in Table 2.

### 3.4. Enzymatic Activity

Succinate dehydrogenase (SDH) enzymatic activity was determined using an SDH assay kit (Solarbio, BC0955), according to a previously reported procedure [48,49]. *Cytospora* sp. was grown in sterile potato dextrose broth (PDB) for 5 d and then treated with various concentrations of selected compounds and the SDHI fungicide fluxapyroxad [50]. After 48 h of treatment with these compounds, the mycelia were filtered, and SDH enzymatic activity was measured using the SDH assay kit according to the manufacturer’s instructions. Each sample was tested in triplicate, and the mean value was used to calculate the inhibition rate.

### 3.5. In Vivo Efficacy of Compound II-a-10

Based on the aforementioned in vitro antifungal activity tests, compound II-a-10 was further tested against apple rot. The synthesized compounds and the positive control fluxapyroxad were dissolved in 10 mL of deionized water at concentrations of 200, 100, 50, and 25 μg/mL in 0.1 mL of DMSO. Each apple was punctured with an inoculation needle and inoculated with the pathogen. After 24 h, each sample measured in triplicate was evenly sprayed onto apples that had been cleaned and treated with water and 75% aqueous ethanol. DMSO (1%) in 10 mL water was used as a blank control. All treated samples were placed in a light incubator at 25 °C and 100% relative humidity for 9 d. All assays were performed at least in triplicate using conventional methods [51].

## 4. Conclusions

In summary, a series of novel diamide compounds combining pyrazolyl and polyfluoro-substituted phenyl groups. Preliminary bioassay results indicated that compounds I-4, II-a-10, and III-26 had potent activities against *Aphis craccivora*. Compound II-a-14 exhibited moderate activity against *Tetranychus cinnabarinus*. In addition, compounds I-1, and II-a-15 showed good insecticidal activity against *Plutella xylostella*. Preliminary analysis of structure–activity relationship (SAR) indicated that the methoxy-substituted hexafluoroisopropyl group and variations in the position of the phenyl ring markedly affected the acaricidal and insecticidal activities. In addition, different types of pyrazolyl influence the biological activity of different pest species. The target compounds showed good fungicidal activities against *Cytospora* sp., *B. cinerea*, and *S. sclerotiorum*. In particular, it is worth noting that polyfluoride-substituted phenyl is necessary for the tested compounds to exhibit their fungicidal activity. The enzymatic activity experiments have shown that the prepared compounds can inhibit the respiration of pathogenic fungi thus having a fungicidal effect. Meanwhile, compound II-a-10 had a comparatively curative effect against *Cytospora* sp. compared to the commercial fungicide fluxapyroxad. Further studies on structural optimization and active mechanisms are currently in progress.

## Figures and Tables

**Table 1:** Insecticidal activities of compounds I-1–III-30 and the comparison compound.

	Against *Aphis craccivora*			Against *Tetranychus cinnabarinus*		Against *Plutella xylostella*	
Compd	400 μg/mL	200 μg/mL	100 μg/mL	400 μg/mL	200 μg/mL	400 μg/mL	200 μg/mL
I-1	85.0%	71.7%	60.0%	65.6%	47.8%	86.7%	76.7%
I-2	81.7%	61.7%	41.7%	53.3%	33.3%	65.6%	47.8%
I-3	76.7%	53.3%	30.0%	52.2%	33.3%	66.7%	40.0%
I-4	93.3%	78.3%	60.0%	53.3%	26.7%	65.6%	46.7%
I-5	80.0%	68.3%	53.3%	70.0%	50.0%	66.7%	33.3%
II-a-6	85.0%	71.7%	60.0%	50.0%	33.3%	50.0%	30.0%
II-a-7	88.3%	73.3%	58.3%	66.7%	51.1%	33.3%	16.7%
II-a-8	68.3%	56.7%	41.7%	63.3%	46.7%	46.7%	23.3%
II-a-9	80.0%	63.3%	50.0%	60.0%	38.9%	70.0%	50.0%
II-a-10	90.0%	78.3%	61.7%	58.9%	41.1%	46.7%	16.7%
II-a-11	76.7%	61.7%	51.7%	72.2%	57.8%	76.7%	46.7%
II-a-12	86.7%	68.3%	53.3%	60.0%	41.1%	60.0%	36.7%
II-a-13	86.7%	73.3%	55.0%	40.0%	26.7%	65.6%	51.1%
II-a-14	90.0%	73.3%	56.7%	73.3%	61.1%	66.7%	50.0%
II-a-15	73.3%	63.3%	50.0%	50.0%	33.3%	86.7%	70.0%
II-a-16	43.3%	26.7%	10.0%	43.3%	27.8%	46.7%	26.7%
II-a-17	48.3%	36.7%	20.0%	36.7%	20.0%	56.7%	40.0%
II-b-18	50.0%	28.3%	20.0%	47.8%	30.0%	46.7%	13.3%
II-b-19	43.3%	25.0%	10.0%	36.7%	18.9%	30.0%	10.0%
II-b-20	58.3%	45.0%	25.0%	40.0%	23.3%	53.3%	26.7%
II-b-21	46.7%	26.7%	16.7%	44.4%	32.2%	60.0%	40.0%
II-c-22	55.0%	35.0%	10.0%	31.1%	18.9%	43.3%	26.7%
II-c-23	45.0%	25.0%	11.7%	36.7%	20.0%	40.0%	13.3%
II-c-24	65.0%	51.7%	35.0%	25.6%	15.6%	36.7%	10.0%
II-c-25	50.0%	28.3%	16.7%	33.3%	13.3%	40.0%	20.0%
III-26	93.3%	81.7%	65.0%	72.2%	55.6%	46.7%	26.7%
III-27	75.0%	53.3%	26.7%	60.0%	41.1%	43.3%	23.3%
III-28	86.7%	66.7%	46.7%	73.3%	54.4%	36.7%	26.7%
III-29	76.7%	61.7%	45.0%	64.4%	48.9%	56.7%	40.0%
III-30	70.0%	55.0%	45.0%	53.3%	36.7%	80.0%	53.3%
imidacloprid	100%	100%	100%	—	—	—	—
fenpyroximate	—	—	—	100%	100%	—	—
flubendiamide	—	—	—	—	—	100%	100%

**Table 2:** In vitro fungicidal activity of compounds I-1–III-30.

	Fungicidal Activity (%) at 50 μg/mL					
Compd	*R. s* ^a^	*C. s*	*B. c*	*F. g*	*P. a*	*S. s*
I-1	32%	70%	55%	40%	29%	81%
I-2	52%	56%	0%	41%	35%	55%
I-3	53%	45%	1%	26%	7%	47%
I-4	24%	58%	18%	24%	18%	60%
I-5	0%	70%	31%	33%	3%	0%
II-a-6	22%	45%	70%	40%	13%	78%
II-a-7	47%	58%	31%	51%	34%	60%
II-a-8	58%	77%	29%	45%	27%	75%
II-a-9	11%	37%	78%	35%	0%	32%
II-a-10	32%	81%	75%	49%	19%	81%
II-a-11	27%	75%	66%	43%	15%	79%
II-a-12	61%	71%	36%	58%	35%	68%
II-a-13	50%	65%	0%	16%	21%	64%
II-a-14	23%	45%	11%	16%	15%	47%
II-a-15	0%	64%	45%	45%	8%	0%
II-a-16	2%	6%	16%	15%	0%	39%
II-a-17	9%	73%	78%	52%	2%	85%
II-b-18	0%	0%	8%	35%	0%	0%
II-b-19	0%	1%	20%	31%	4%	0%
II-b-20	5%	0%	7%	8%	0%	0%
II-b-21	10%	0%	25%	9%	0%	34%
II-c-22	13%	1%	19%	44%	0%	18%
II-c-23	5%	0%	3%	22%	0%	8%
II-c-24	10%	0%	10%	1%	0%	0%
II-c-25	25%	0%	14%	0%	0%	0%
III-26	22%	42%	85%	40%	5%	76%
III-27	57%	44%	9%	29%	20%	43%
III-28	48%	55%	12%	42%	33%	52%
III-29	40%	0%	0%	0%	10%	16%
III-30	27%	31%	54%	38%	1%	55%
fluxapyroxad	97%	78%	84%	16%	31%	90%

^a^*R. s*, *Rhizoctonia solani*; *C. s, Cytospora* sp.; *B. c, Botrytis cinerea*; *F. g, Fusarium graminearum*; *P. a, Pythium aphanidermatum*; *S. s, Sclerotinia sclerotiorum*.

**Table 3:** In vitro EC_50_ values of selected compounds.

Fungi	Compd	EC_50_ (μg/mL)
*Cytospora* sp.	II-a-10	36.169
	fluxapyroxad	19.354
*B. cinerea*	III-26	22.531
	fluxapyroxad	0.835
*S. sclerotiorum*	I-1	14.711
	II-a-10	17.683
	II-a-17	8.114
	fluxapyroxad	0.334

**Table 4:** In vivo curative effect of compound II-a-10 against *Cytospora* sp.

Compd	Conc. (μg/mL)	5 Day Control Efficacy (%)	9 Day Control Efficacy (%)
II-a-10	200	84.21%	83.78%
	100	73.69%	77.70%
	50	71.06%	73.65%
	25	52.64%	59.46%
fluxapyroxad	200	89.48%	87.16%
	100	81.58%	78.38%
	50	76.32%	76.35%
	25	55.27%	53.38%

## Data Availability

Not applicable.

## Supplementary Materials

The following supporting information can be downloaded at: https://www.mdpi.com/article/10.3390/molecules28020561/s1.^1^H and ^13^C NMR spectral data of compounds I-1–III-30.
