# Supplementary material for: Design, Synthesis, Fungicidal and Insecticidal Activities of Novel Diamide Compounds Combining Pyrazolyl and Polyfluoro-Substituted Phenyl into Alanine or 2-Aminobutyric Acid Skeletons
Source: Molecules. 2023 Jan 5;28(2):561. doi: 10.3390/molecules28020561 (PMC9861274; doi:10.3390/molecules28020561)
Supplement: Supplementary file 1 [file molecules-28-00561-s001.zip › molecules-2097014-supplementary.pdf]

Supplementary Materials:

Design, Synthesis, Fungicidal and Insecticidal Activities of Novel Diamide Compounds Combining Pyrazolyl and Polyfluoro-Substituted Phenyl into Alanine or 2-Aminobutyric acid Skeletons

Zhi-Yuan Xu [1,2], Tong Feng [1,2], Qing Liu [1], Hui-Ting Li [1], Wei Wei [1], Rong-Chuan Shi [1,2], Yi-Ming Cao [1,2], * and Shang-Zhong Liu [1,2], *

[1]Innovation center of Pesticide Research, Department of Applied Chemistry, College of Science, China Agricultural University, Beijing 100193, China;

[2]Key laboratory of National Forestry and Grassland Administration on Pest Chemical Control, China Agricultural University, Beijing 100193, China

*Correspondence: caoym@cau.edu.cn (Y.-M.C.); shangzho@cau.edu.cn (S.-Z.L.); Tel.: +86-10-62731070 (S.-Z.L.)

The NMR spectrogram of title compounds

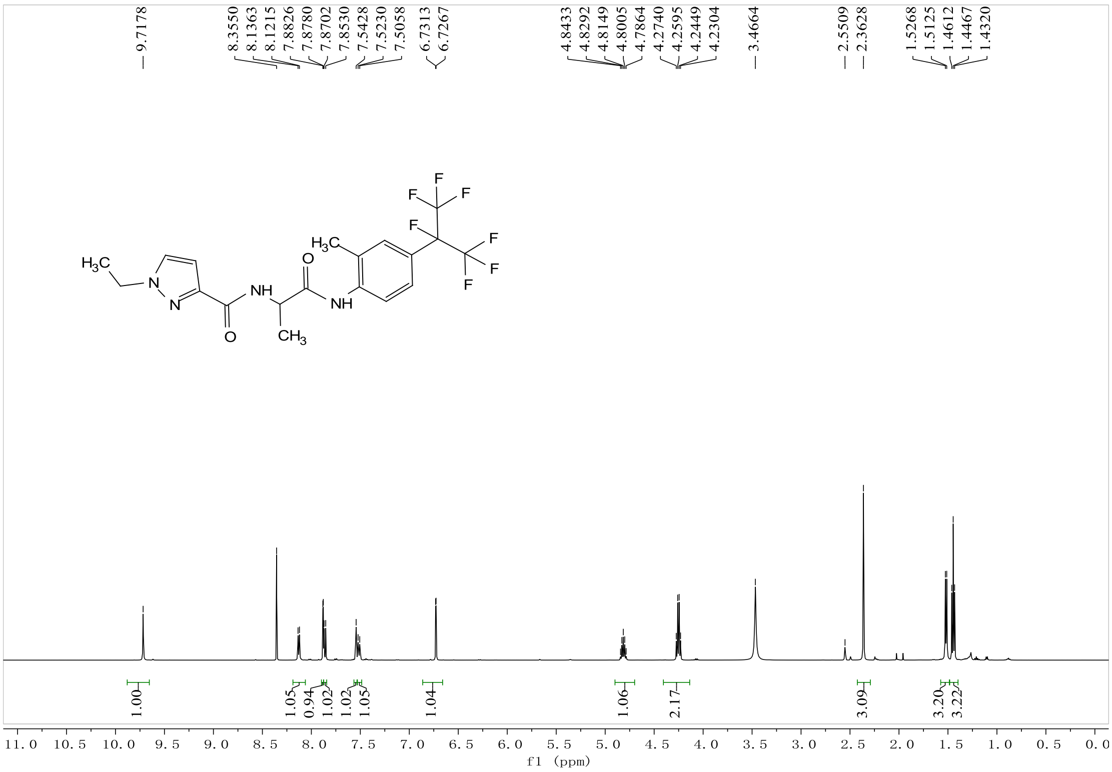

Figure S1. [1] H NMR spectrum of compound I-1.

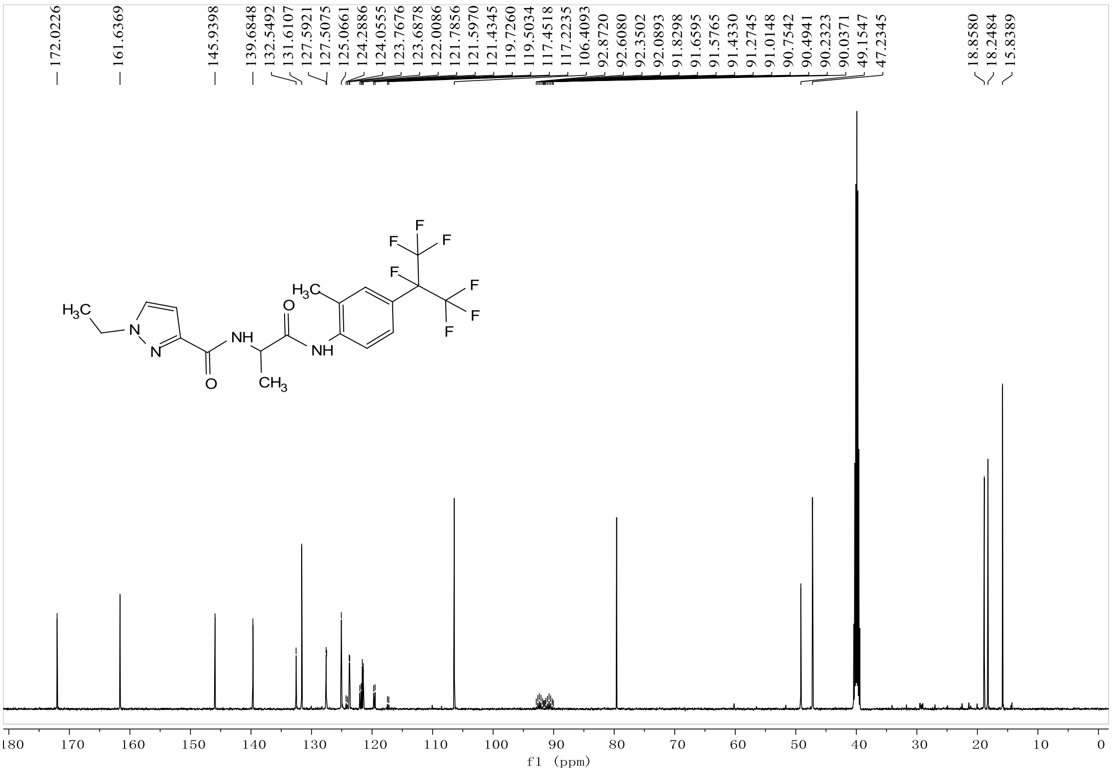

Figure S2. [13] C NMR spectrum of compound I-1.

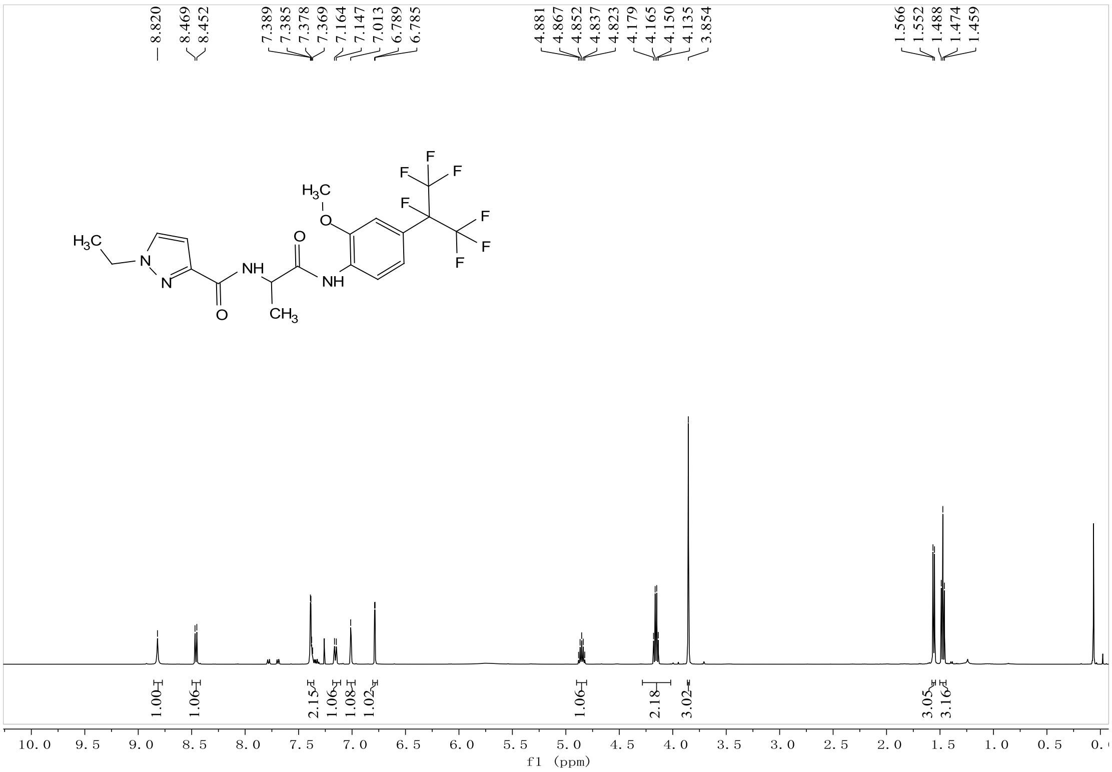

Figure S3. [1] H NMR spectrum of compound I-2.

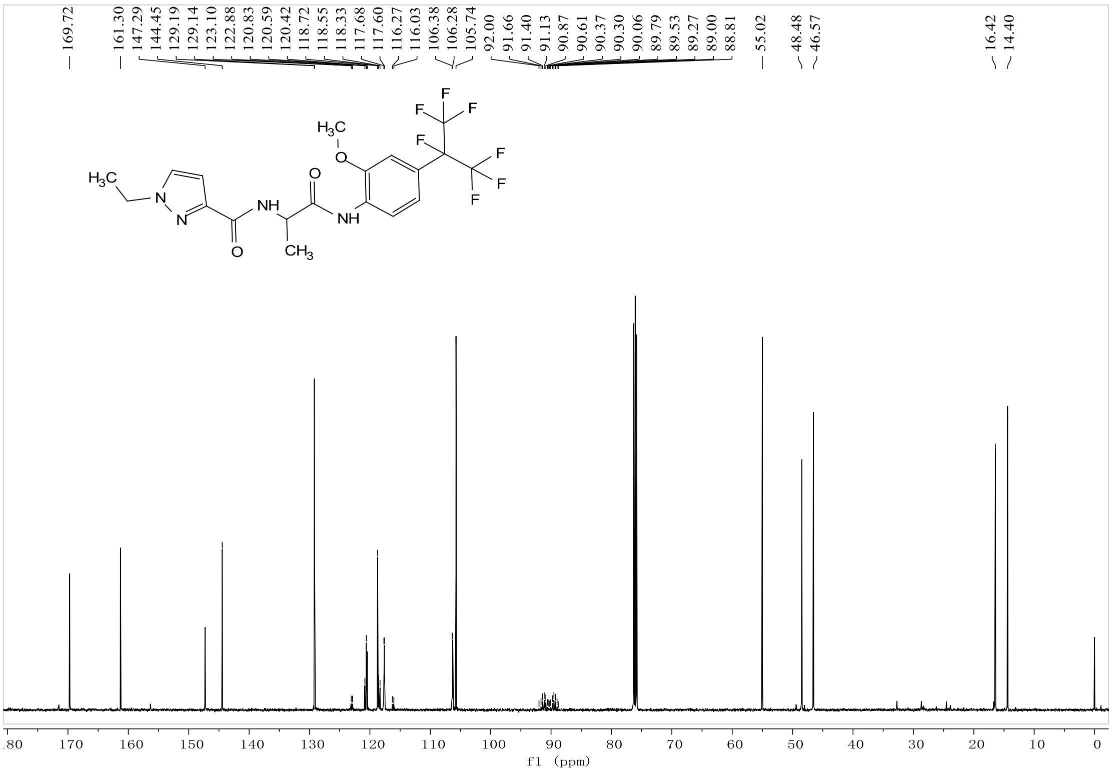

Figure S4. [13] C NMR spectrum of compound I-2.

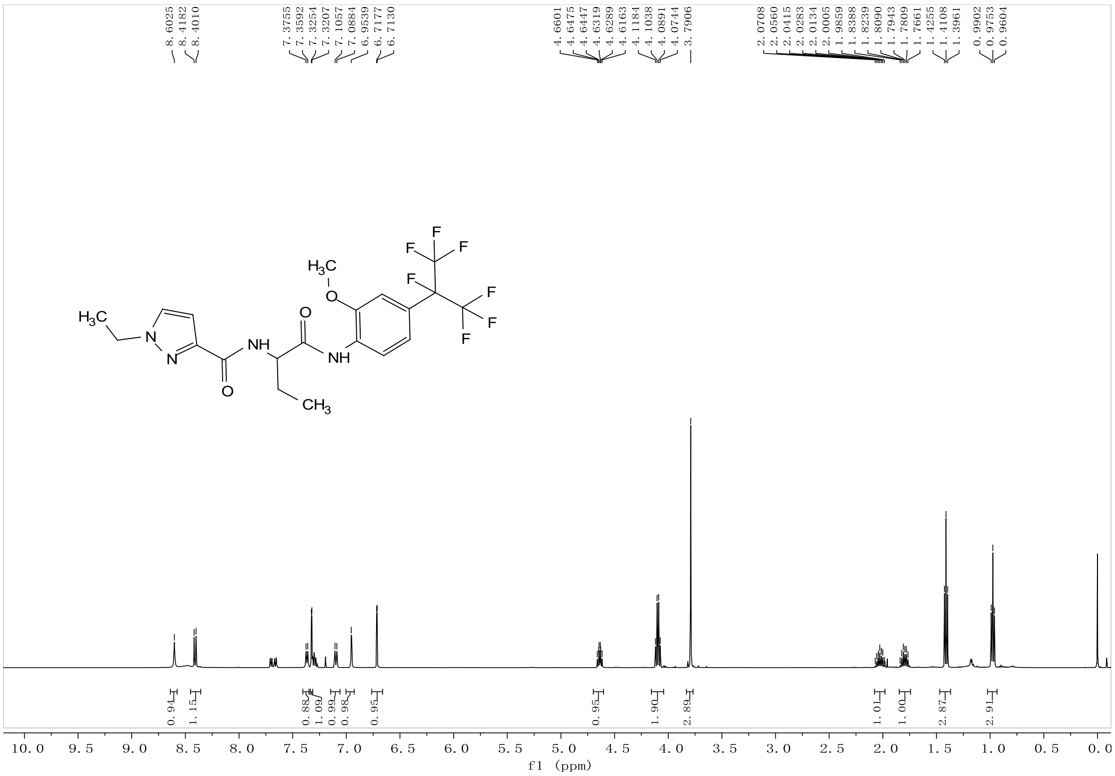

Figure S5. [1] H NMR spectrum of compound I-3.

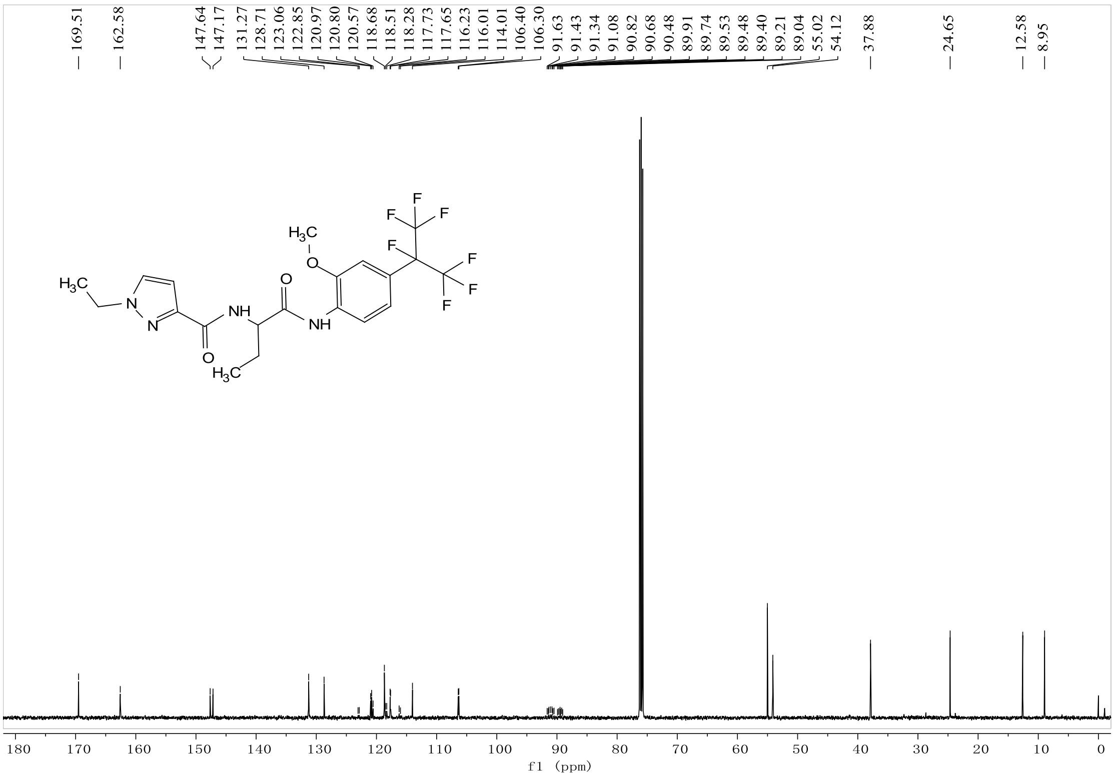

Figure S6. [13] C NMR spectrum of compound I-3.

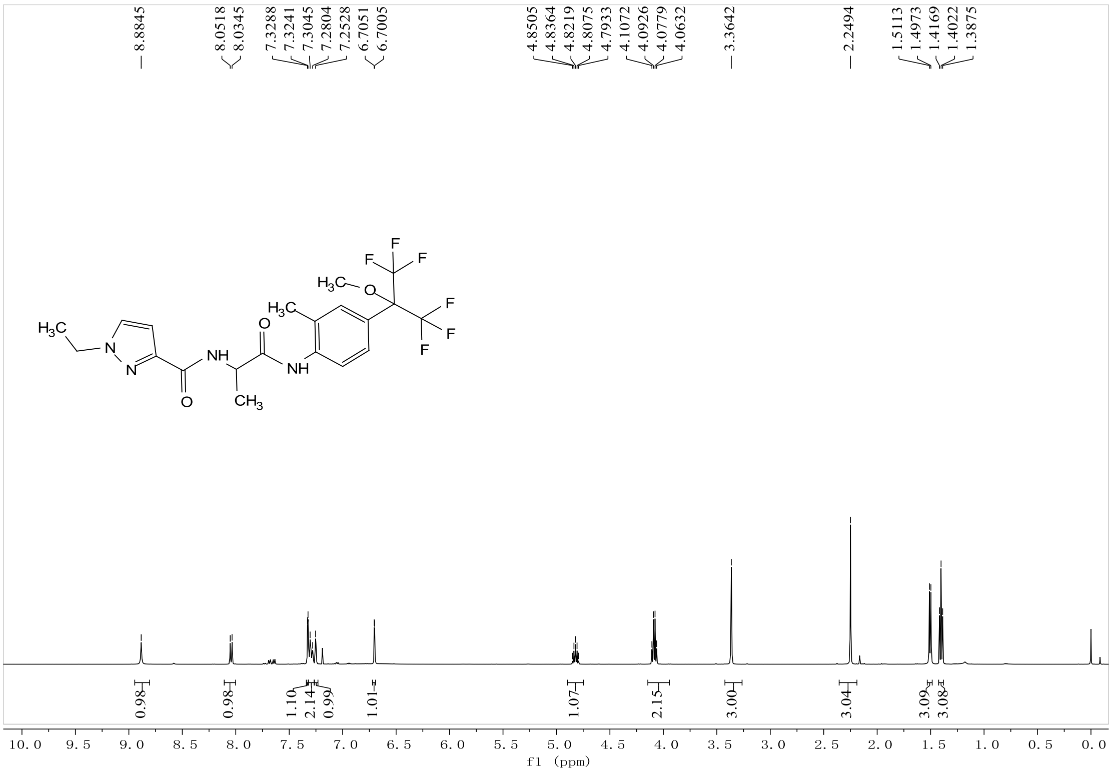

Figure S7. [1] H NMR spectrum of compound I-4.

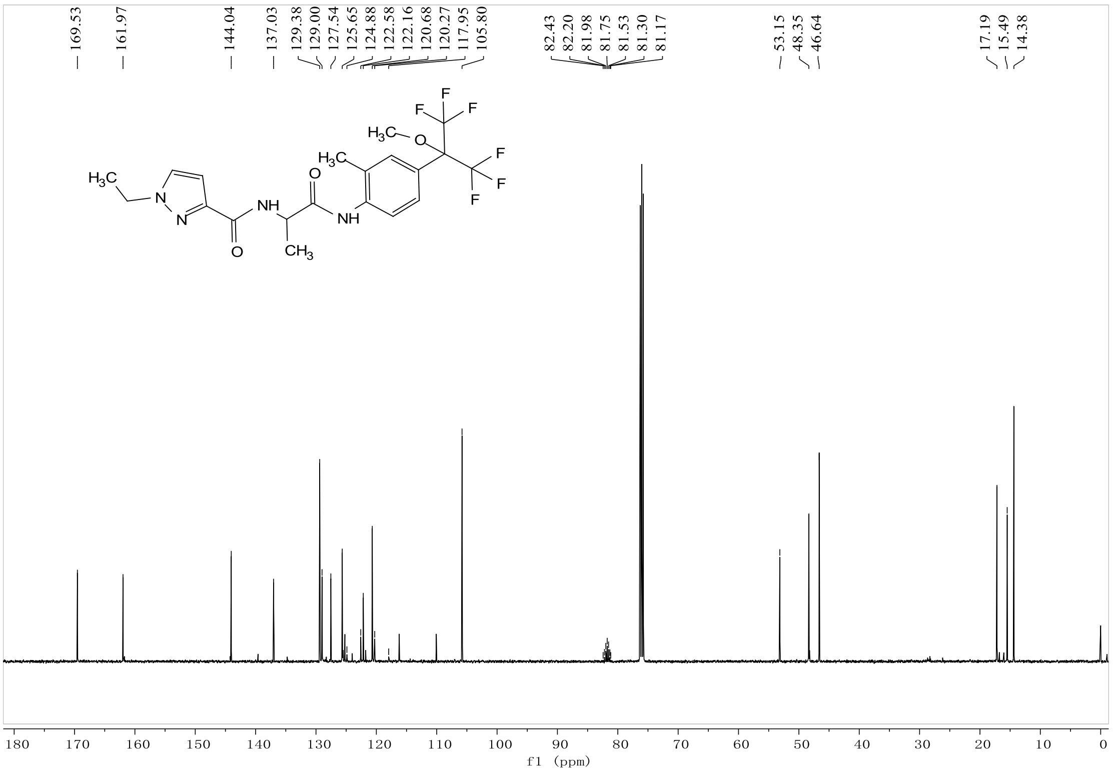

Figure S8. [13] C NMR spectrum of compound I-4.

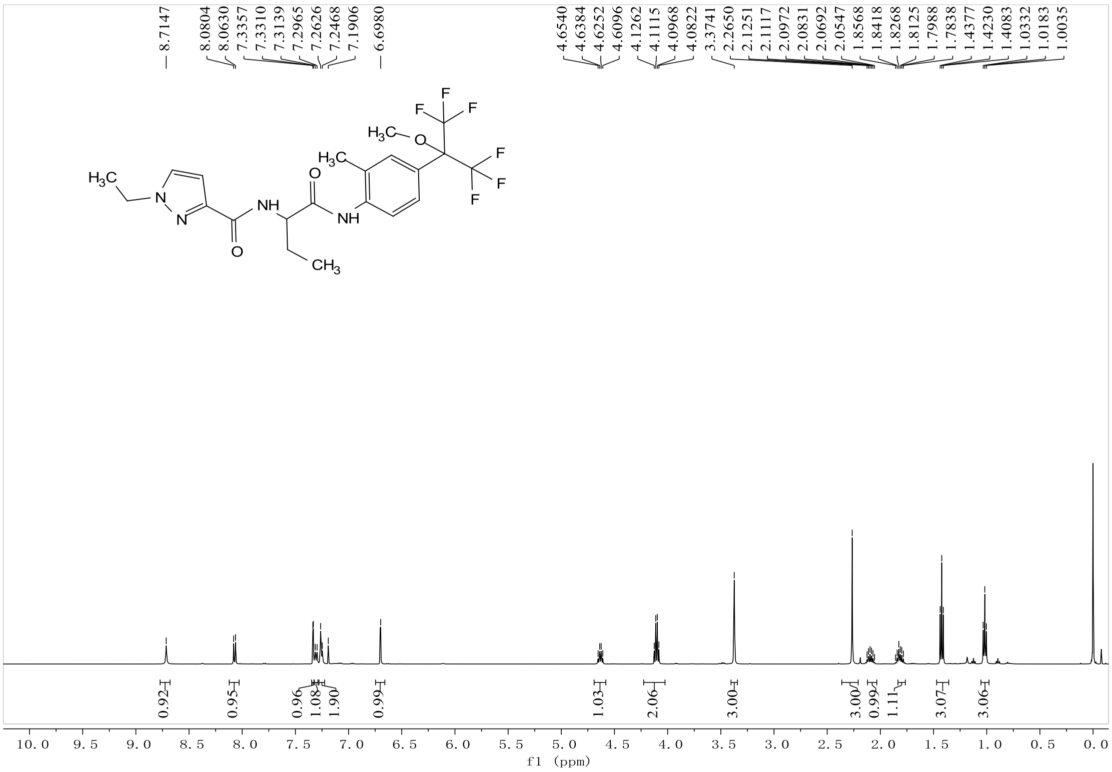

Figure S9. [1] H NMR spectrum of compound I-5.

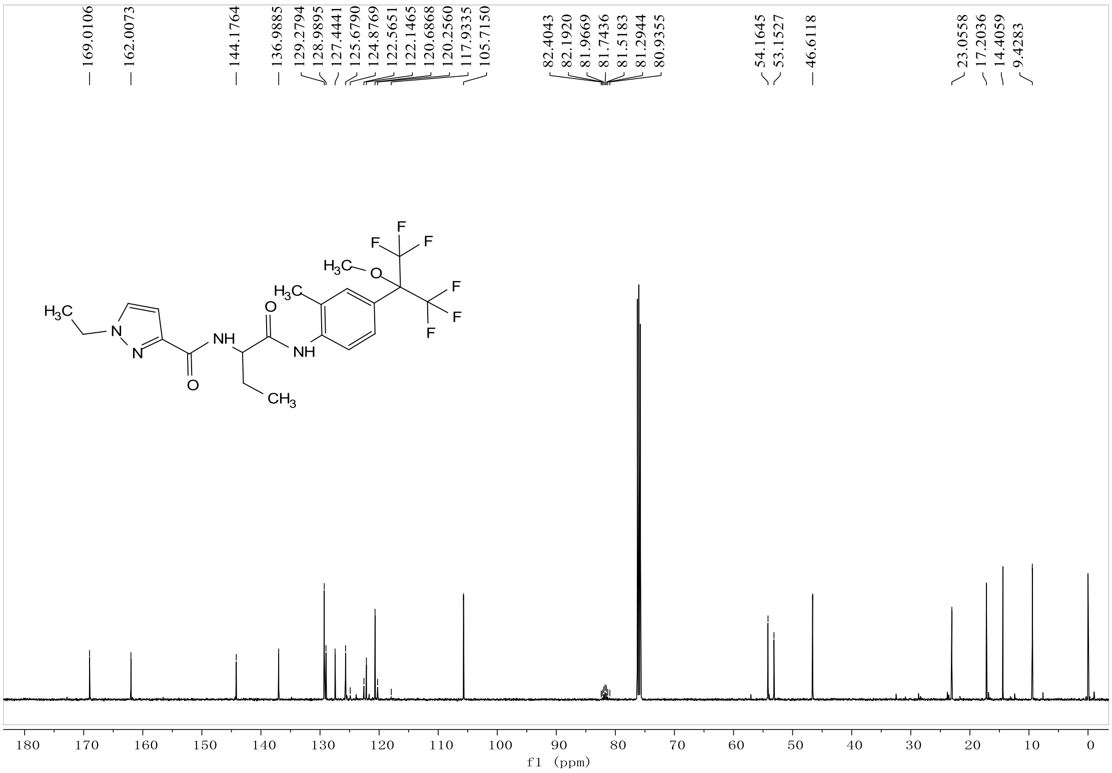

Figure S10. [13] C NMR spectrum of compound I-5.

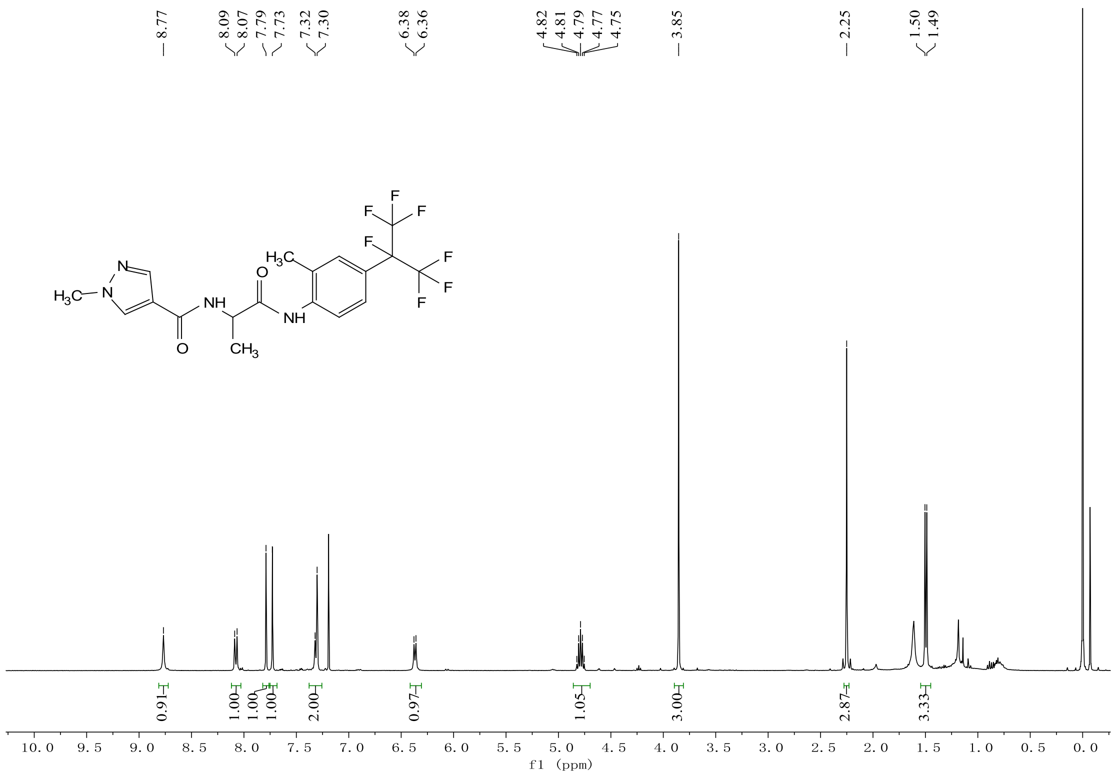

Figure S11. [1] H NMR spectrum of compound II-a-6.

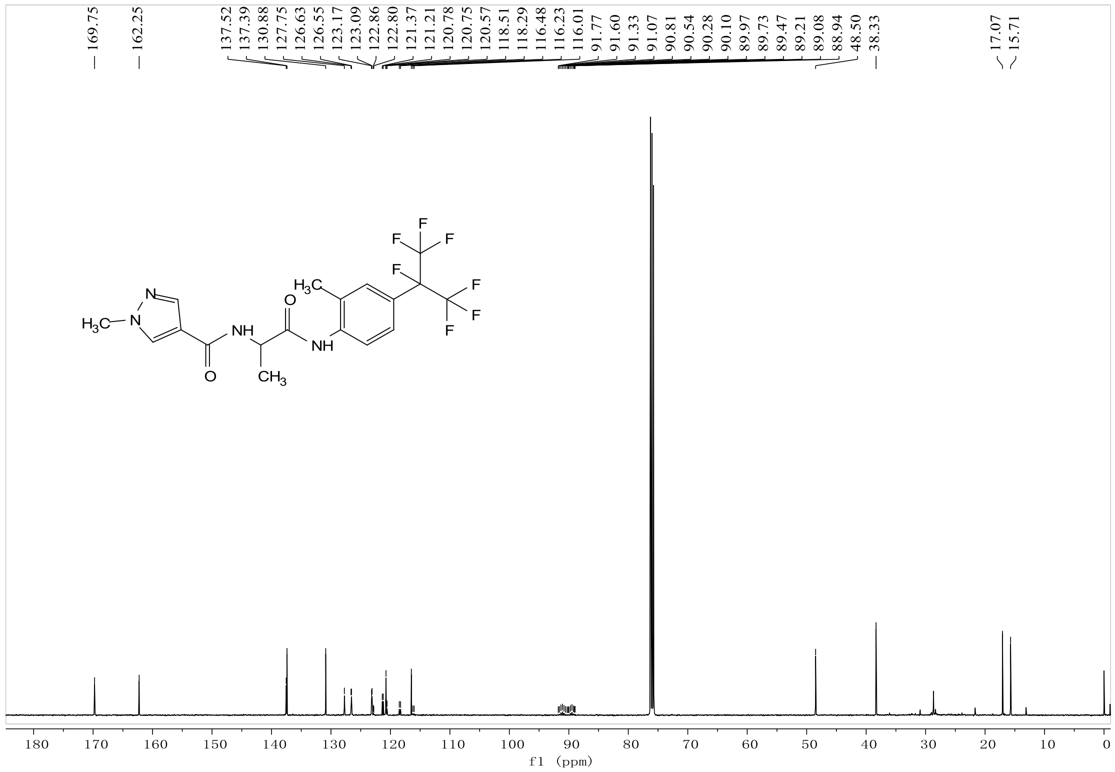

Figure S12. [13] C NMR spectrum of compound II-a-6.

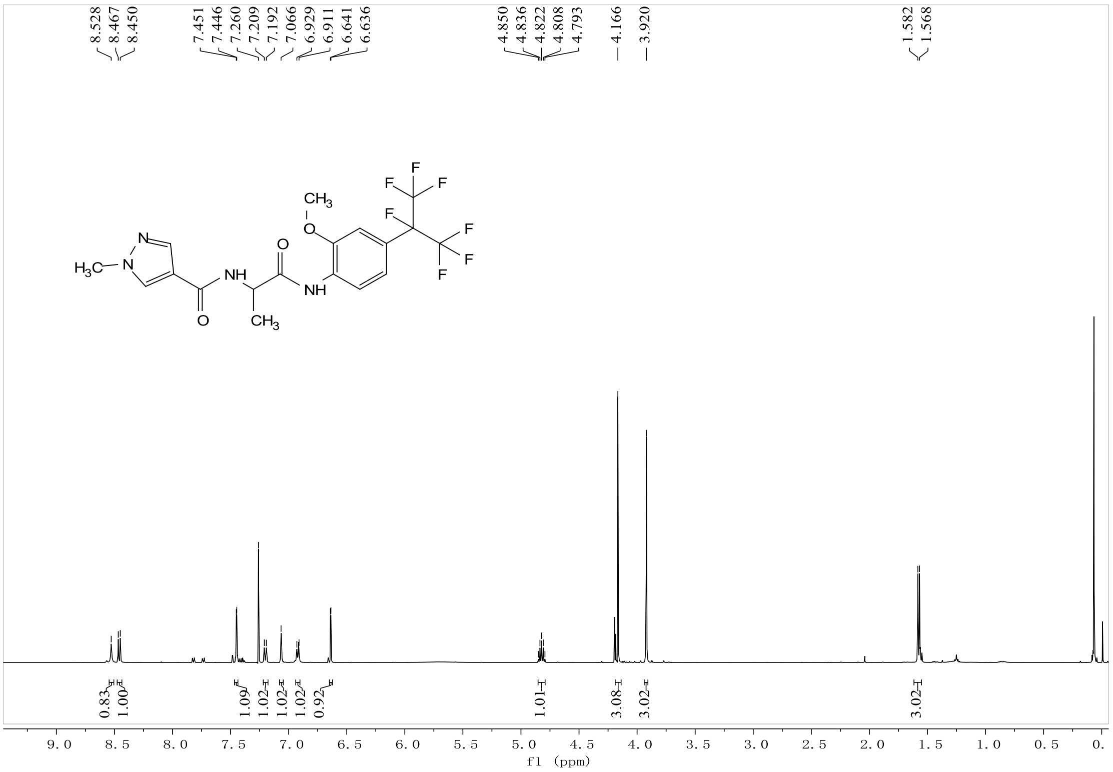

Figure S13. [1] H NMR spectrum of compound II-a-7.

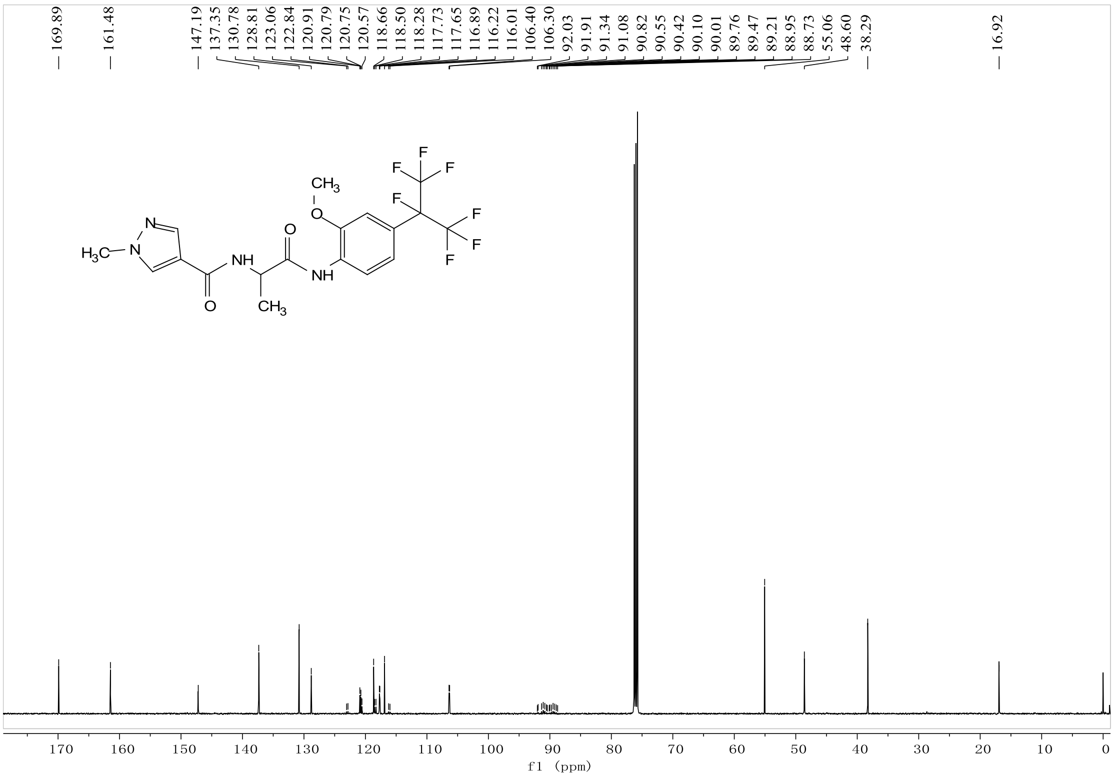

Figure S14. [13] C NMR spectrum of compound II-a-7.

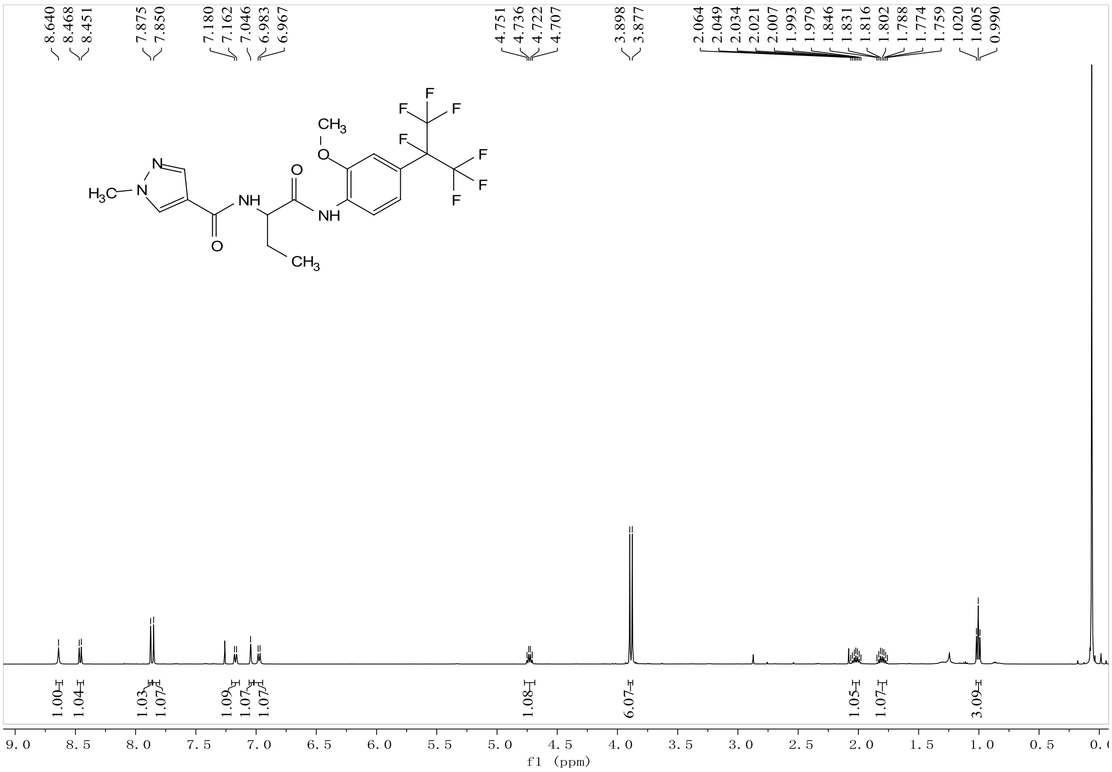

Figure S15. [1] H NMR spectrum of compound II-a-8.

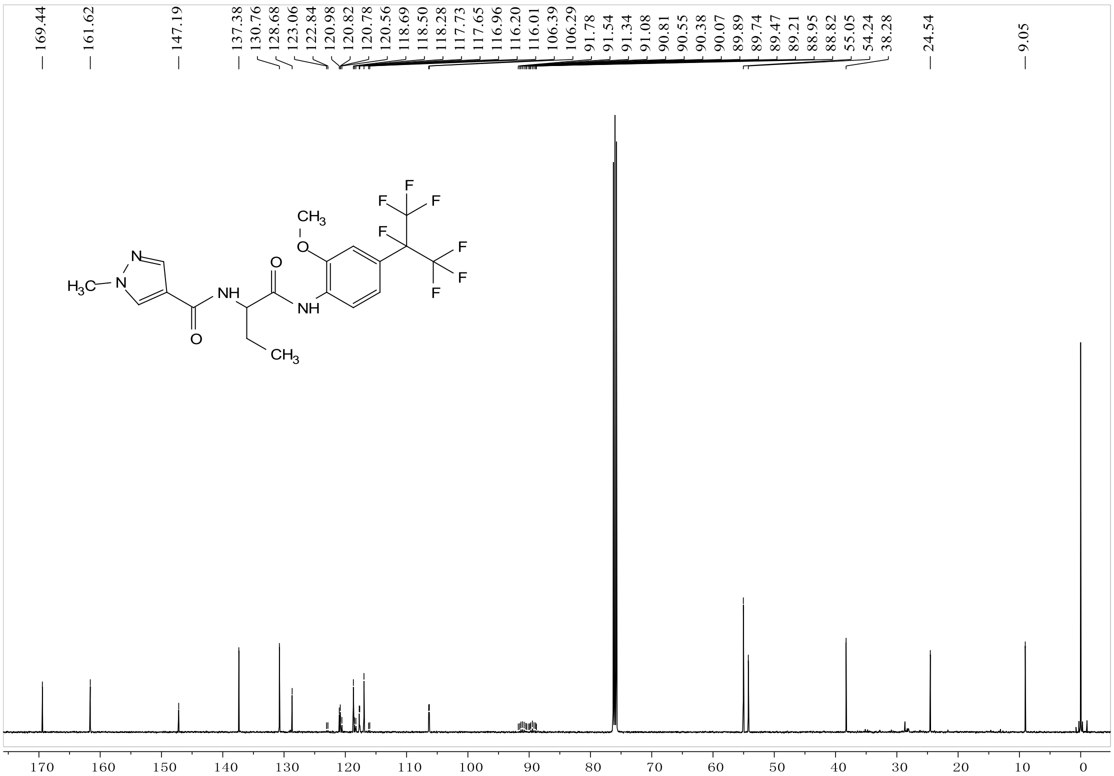

Figure S16. [13] C NMR spectrum of compound II-a-8.

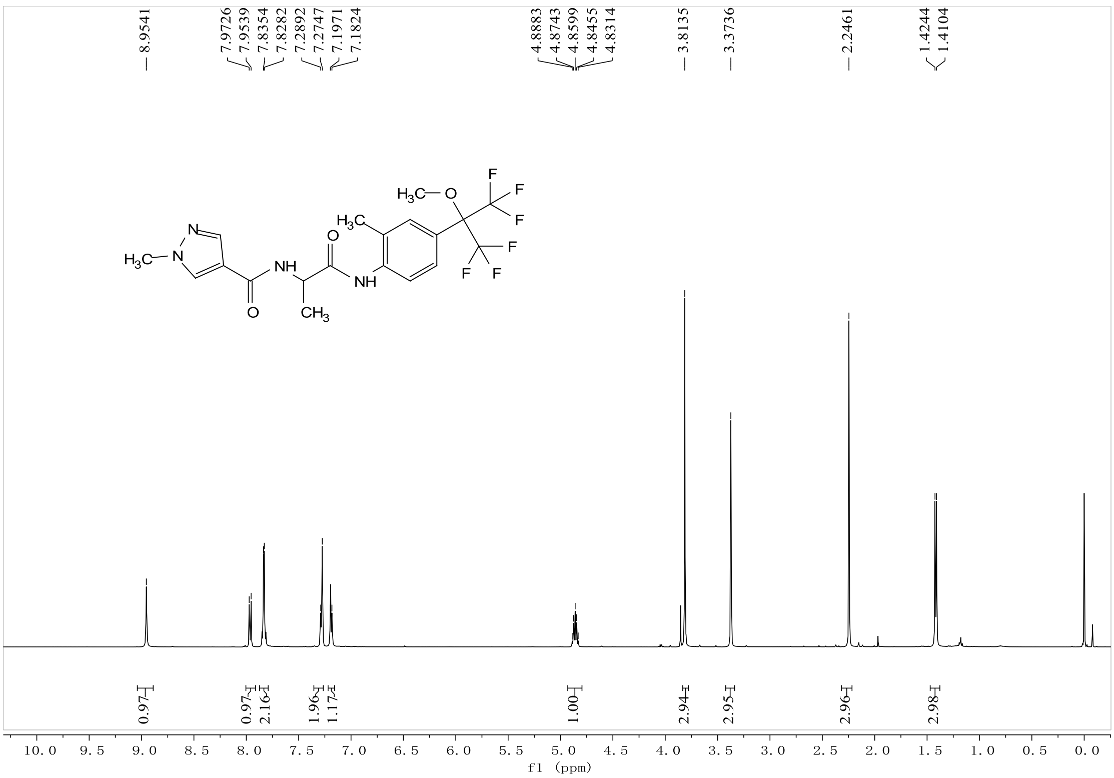

Figure S17. [1] H NMR spectrum of compound II-a-9.

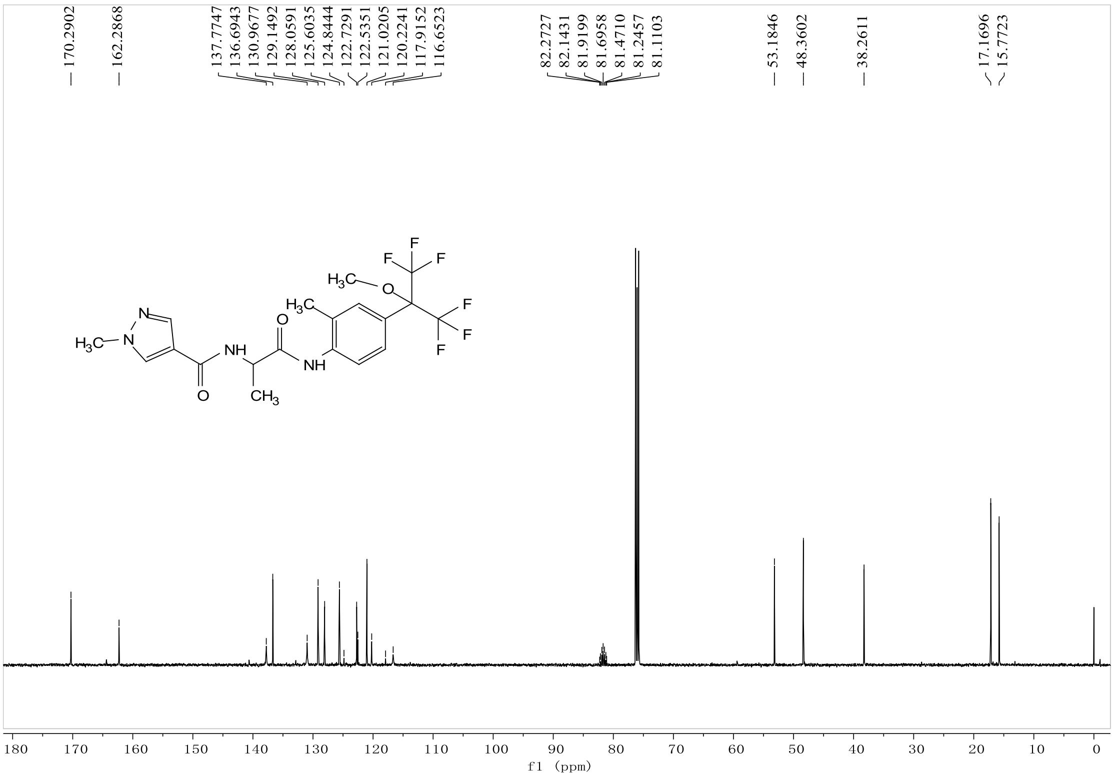

Figure S18. [13] C NMR spectrum of compound II-a-9.

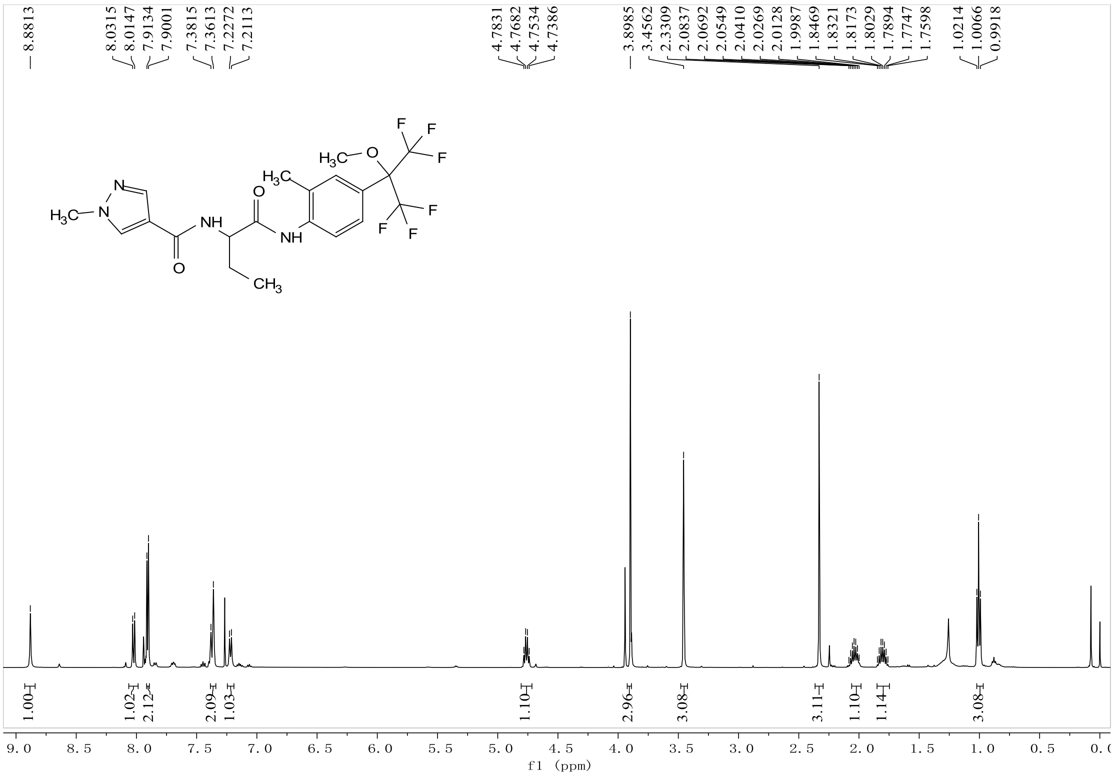

Figure S19. [1] H NMR spectrum of compound II-a-10.

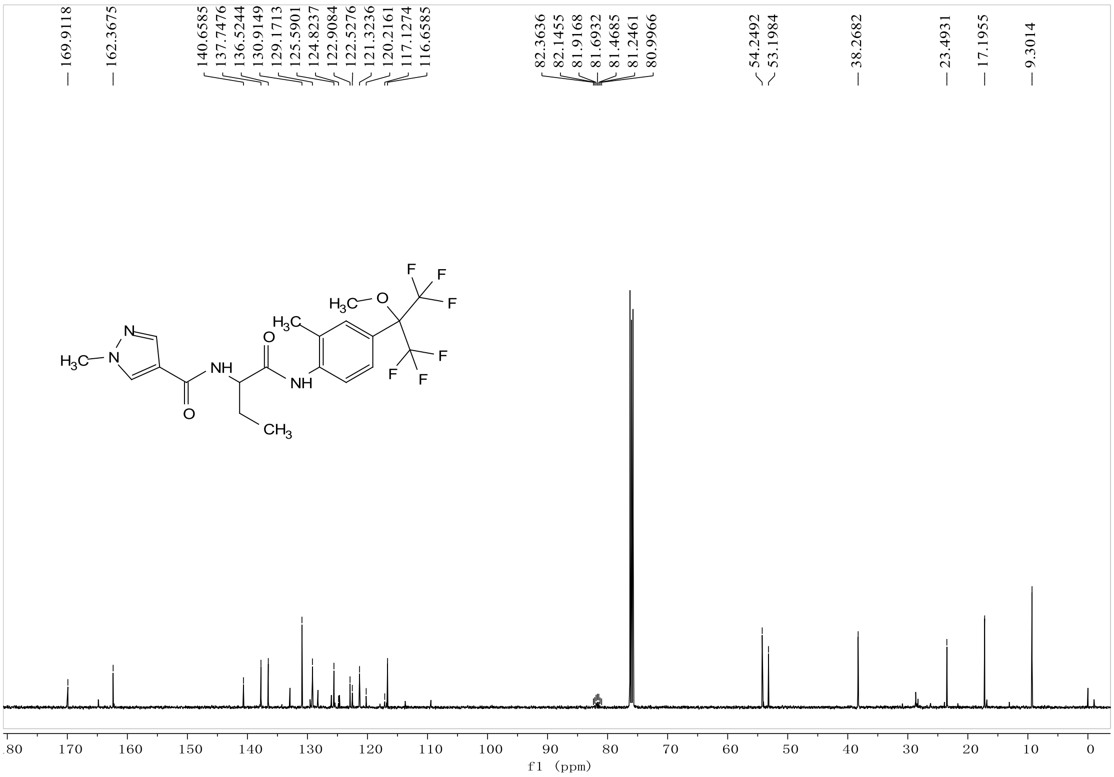

Figure S20. [13] C NMR spectrum of compound II-a-10.

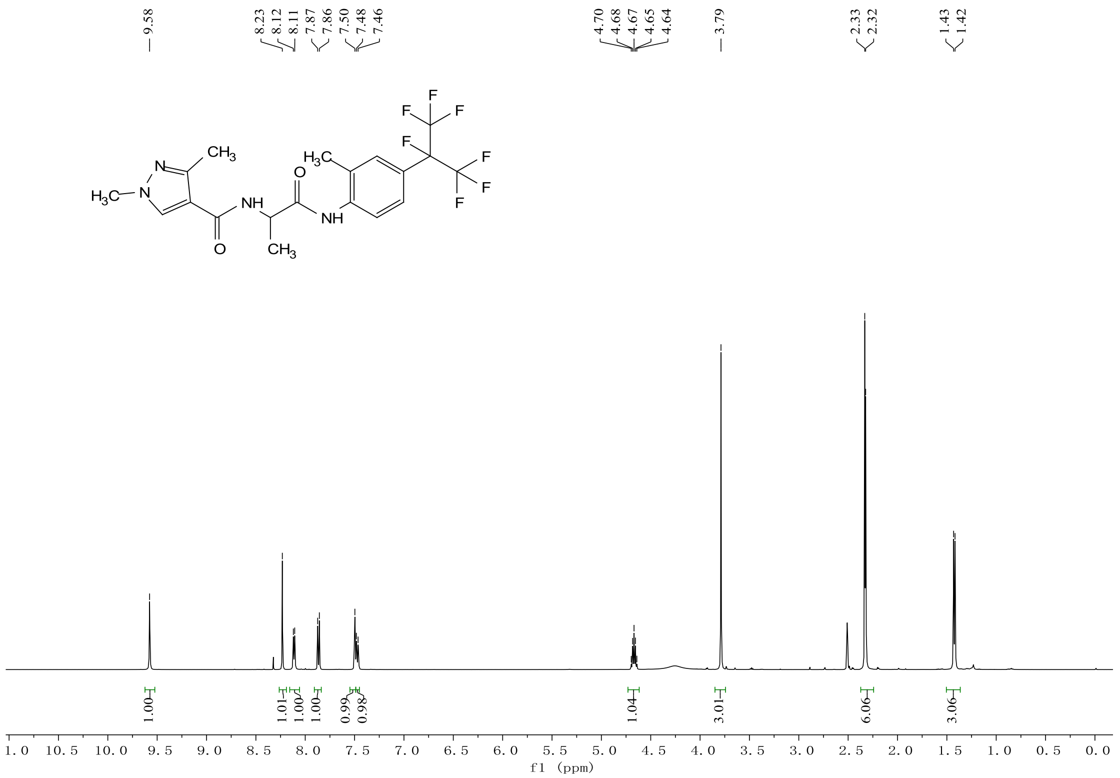

Figure S21. [1] H NMR spectrum of compound II-a-11.

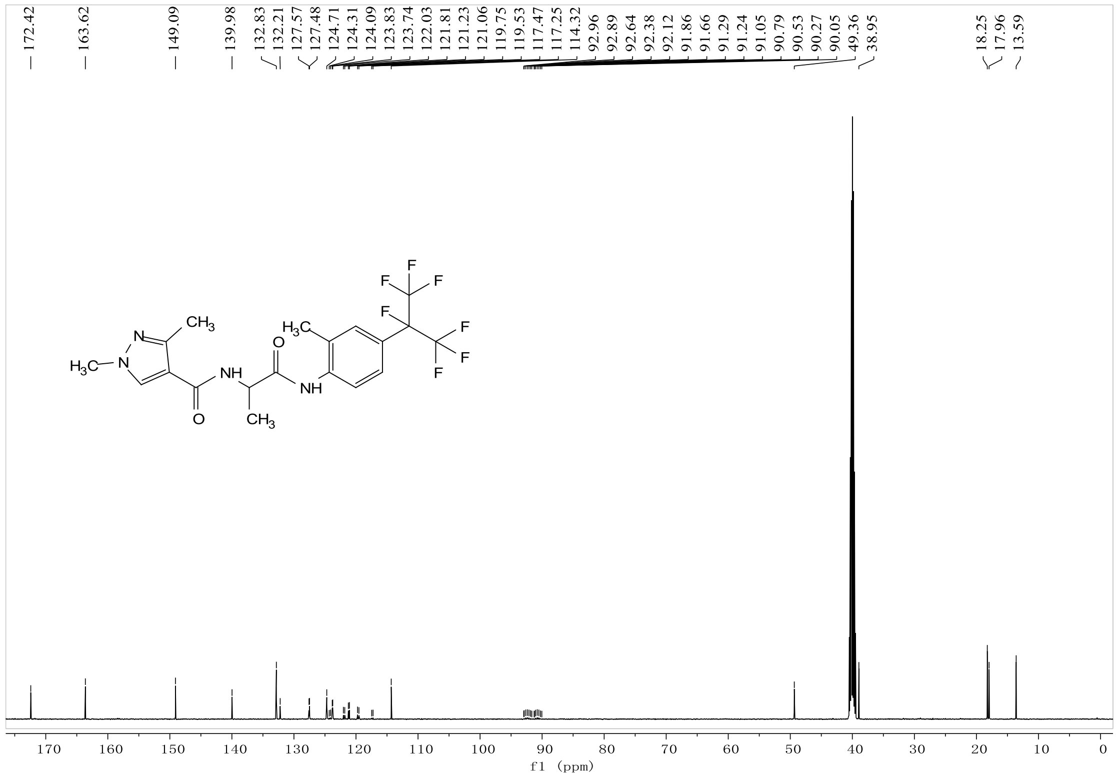

Figure S22. [13] C NMR spectrum of compound II-a-11.

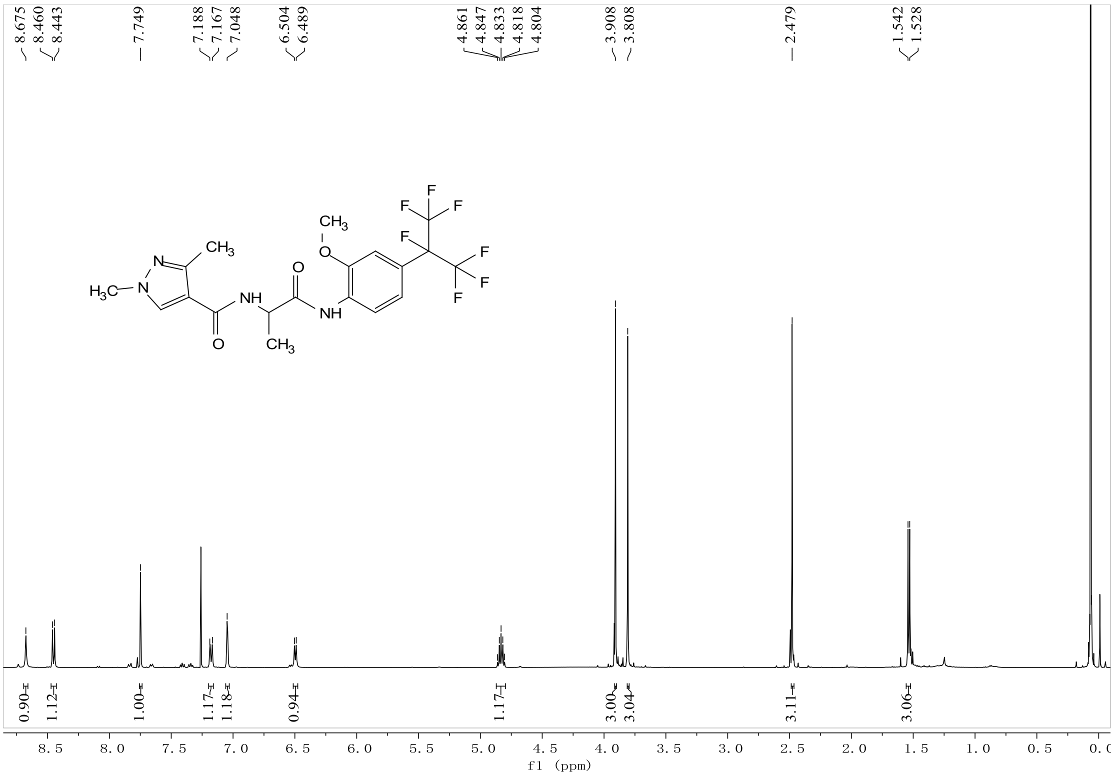

Figure S23. [1] H NMR spectrum of compound II-a-12.

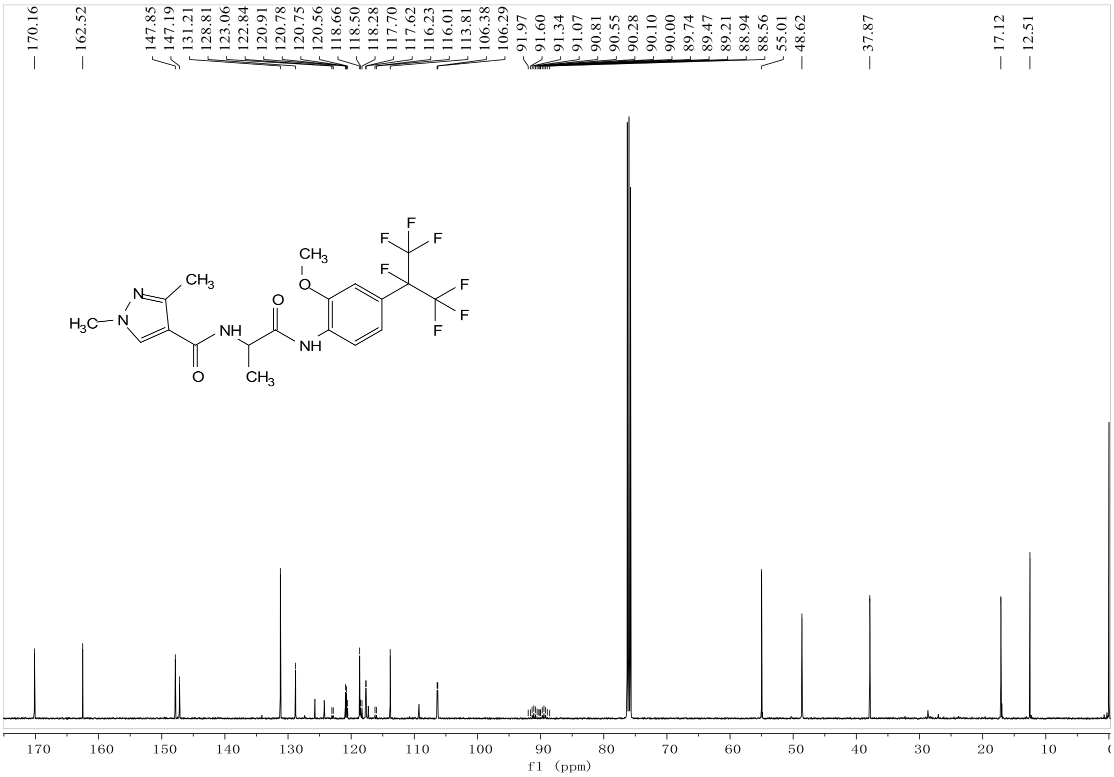

Figure S24. [13] C NMR spectrum of compound II-a-12.

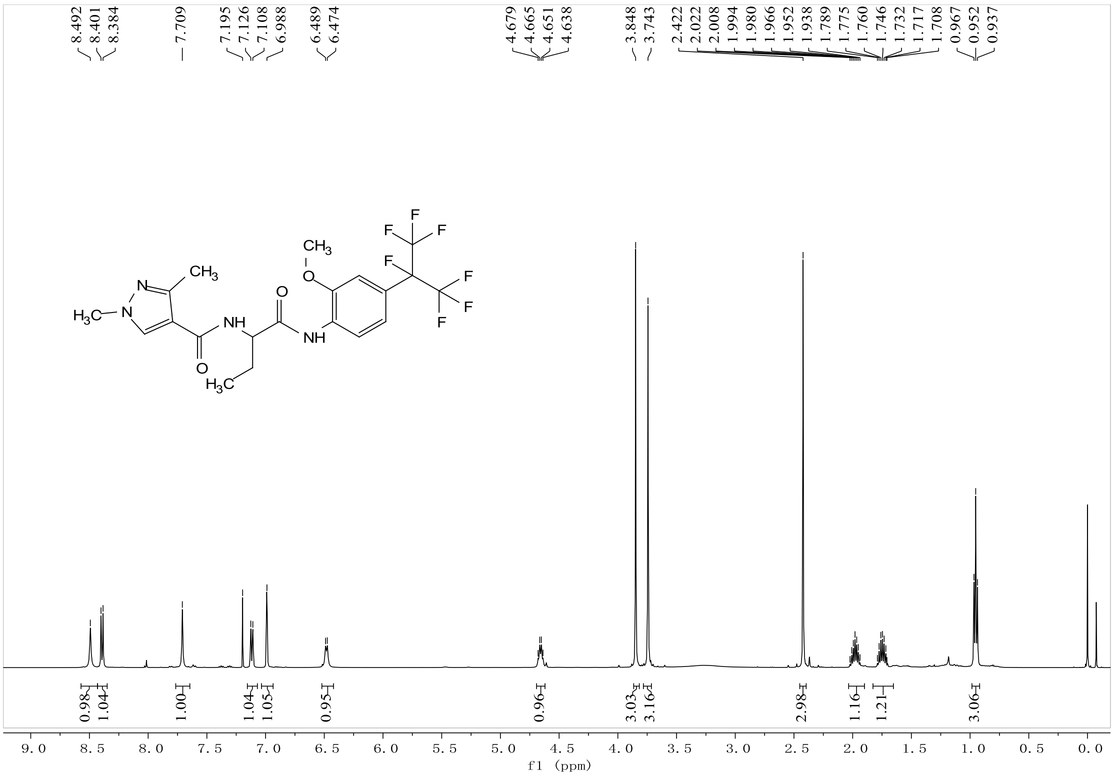

Figure S25. [1] H NMR spectrum of compound II-a-13.

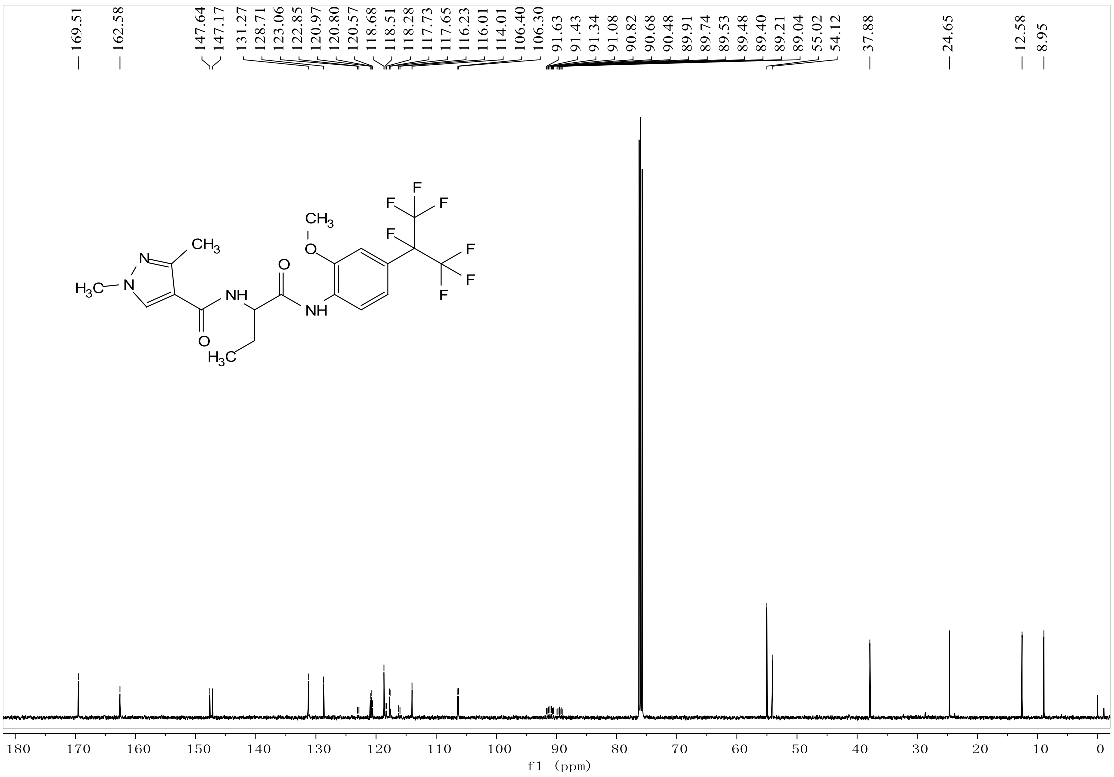

Figure S26. [13] C NMR spectrum of compound II-a-13.

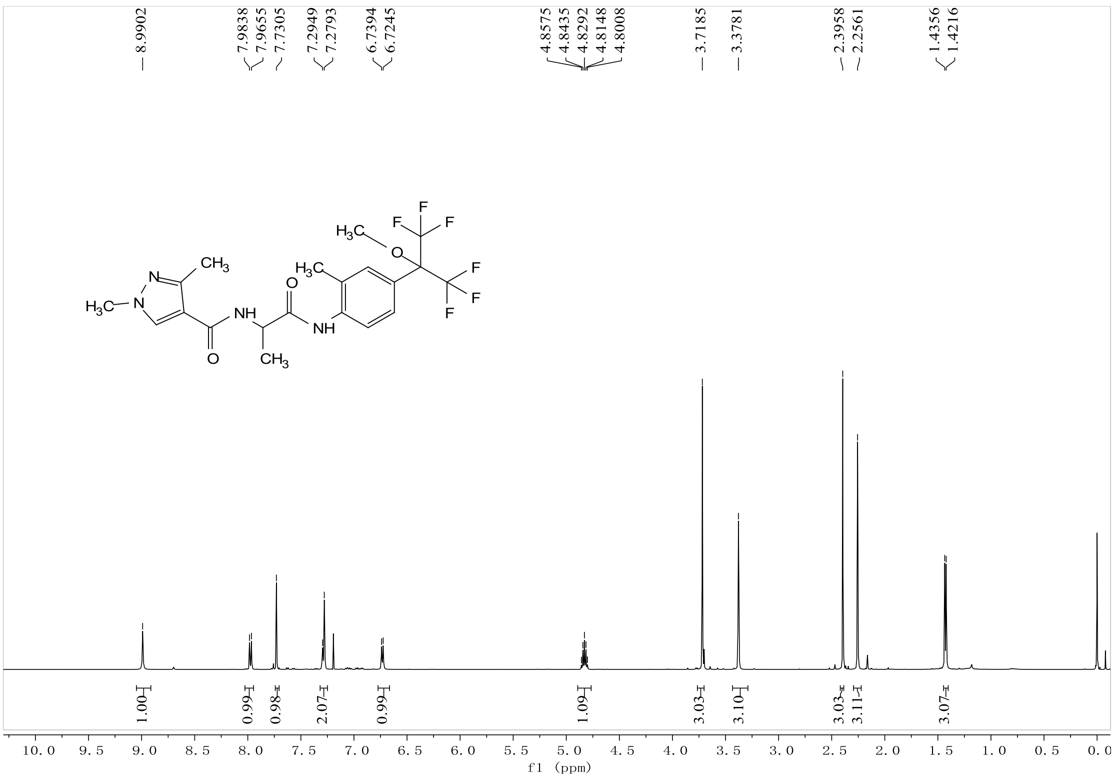

Figure S27. [1] H NMR spectrum of compound II-a-14.

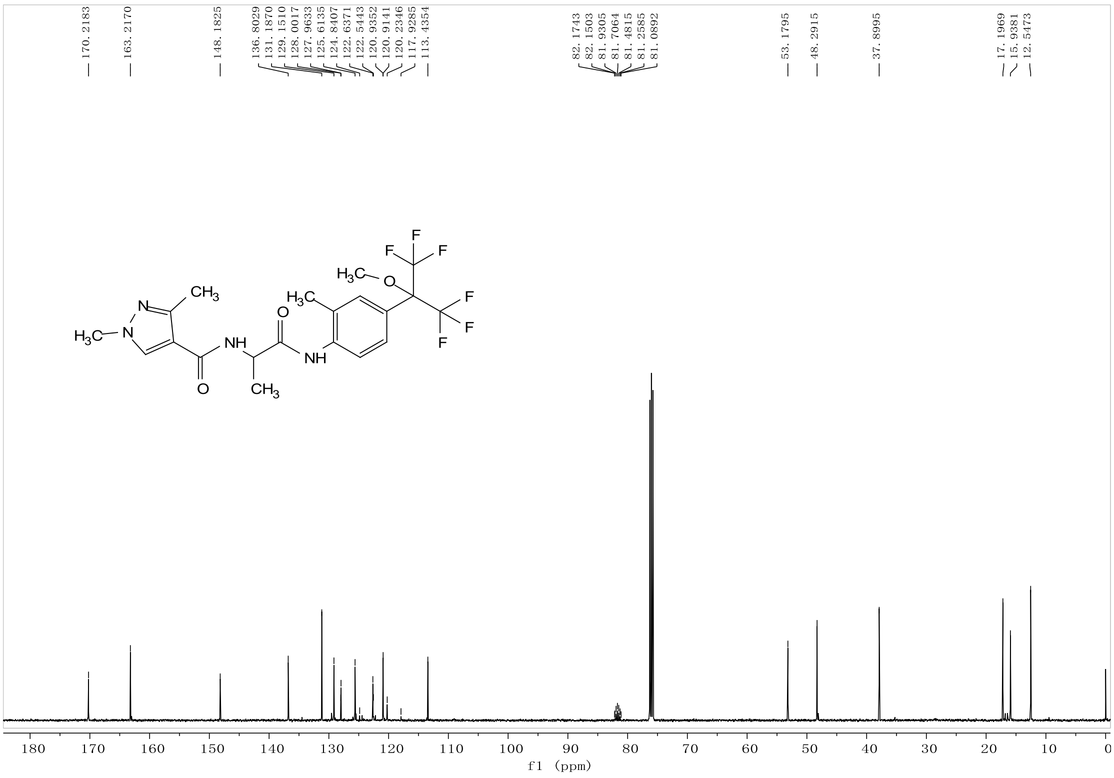

Figure S28. [13] C NMR spectrum of compound II-a-14.

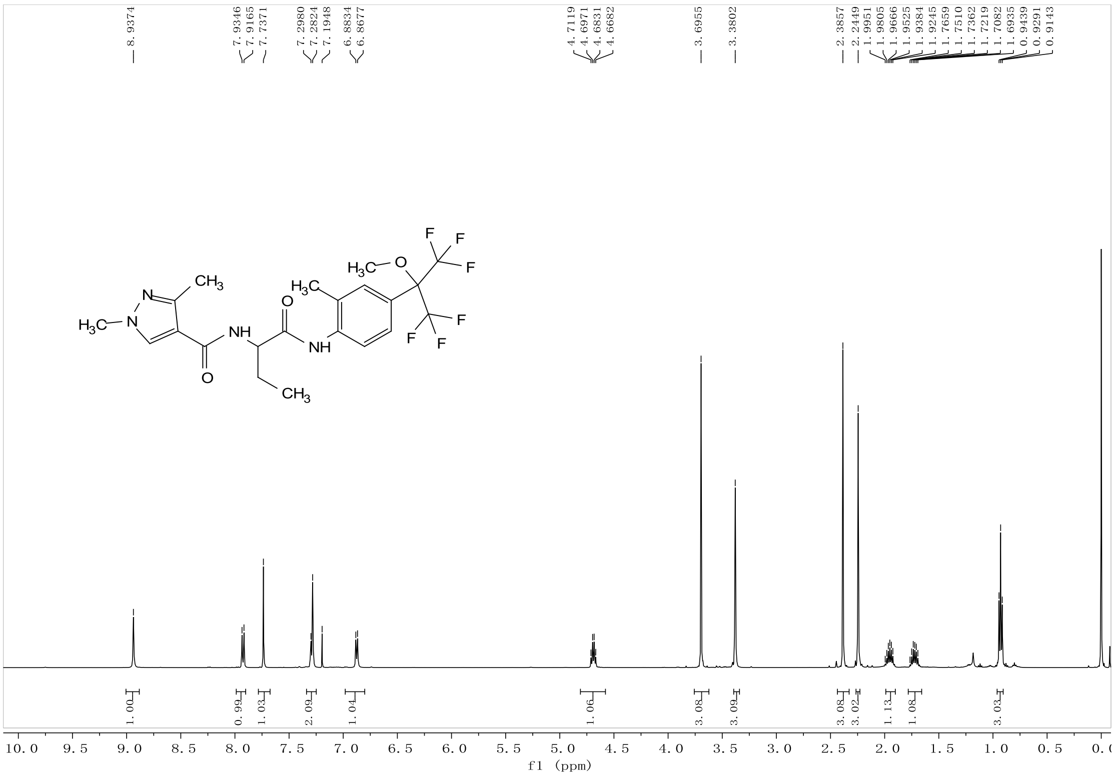

Figure S29. [1] H NMR spectrum of compound II-a-15.

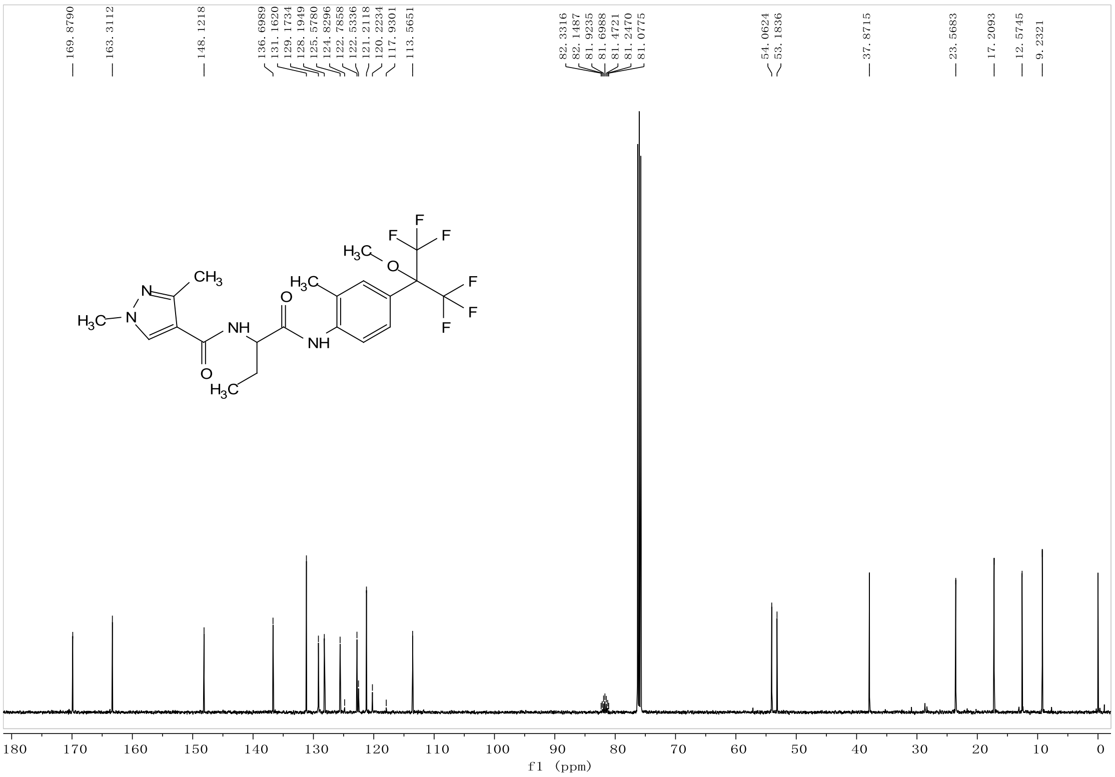

Figure S30. [13] C NMR spectrum of compound II-a-15.

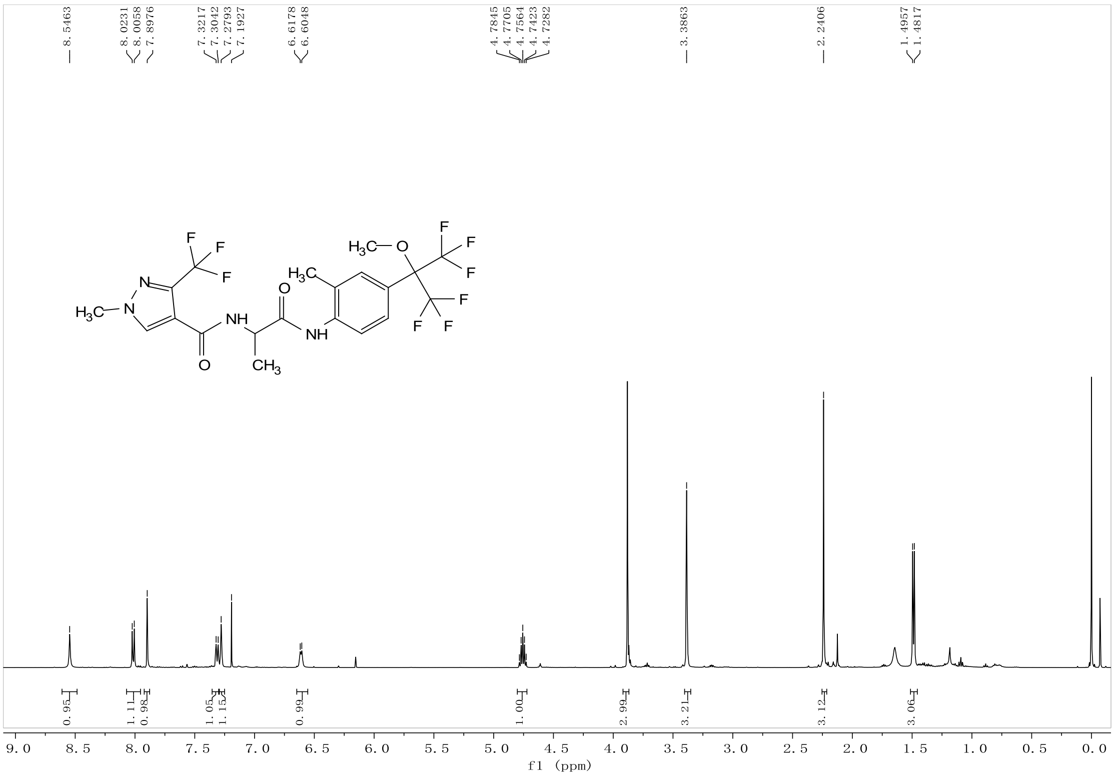

Figure S31. [1] H NMR spectrum of compound II-a-16.

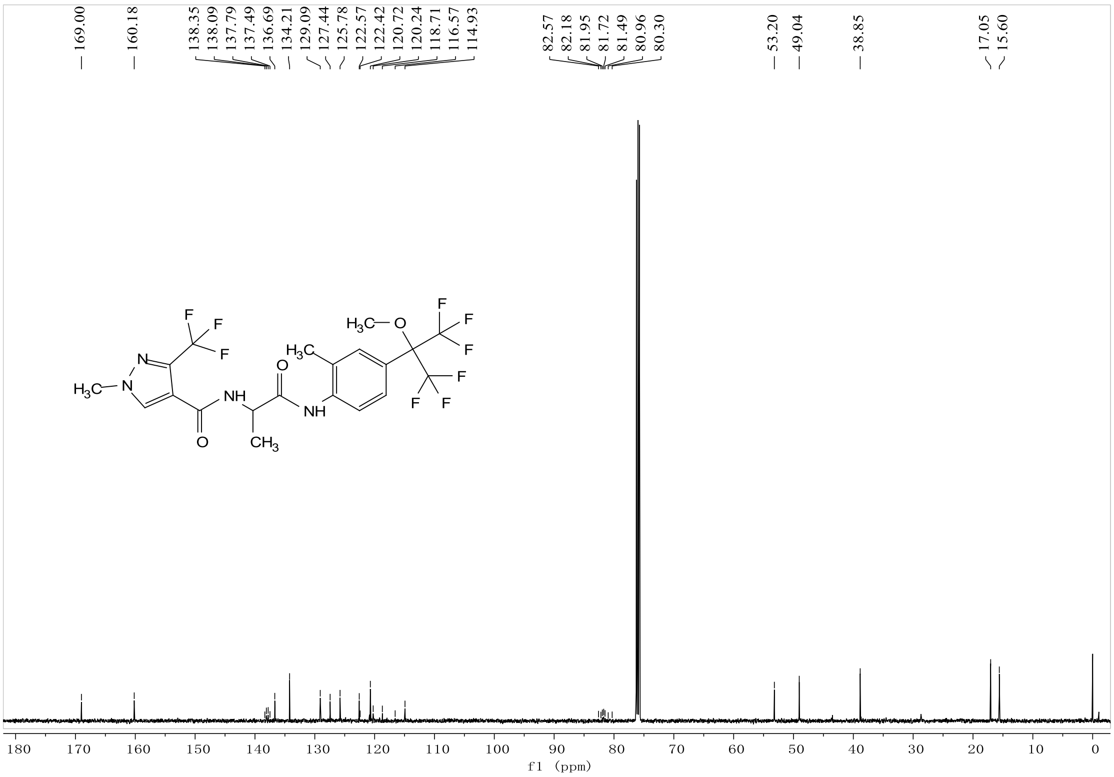

Figure S32. [13] C NMR spectrum of compound II-a-16.

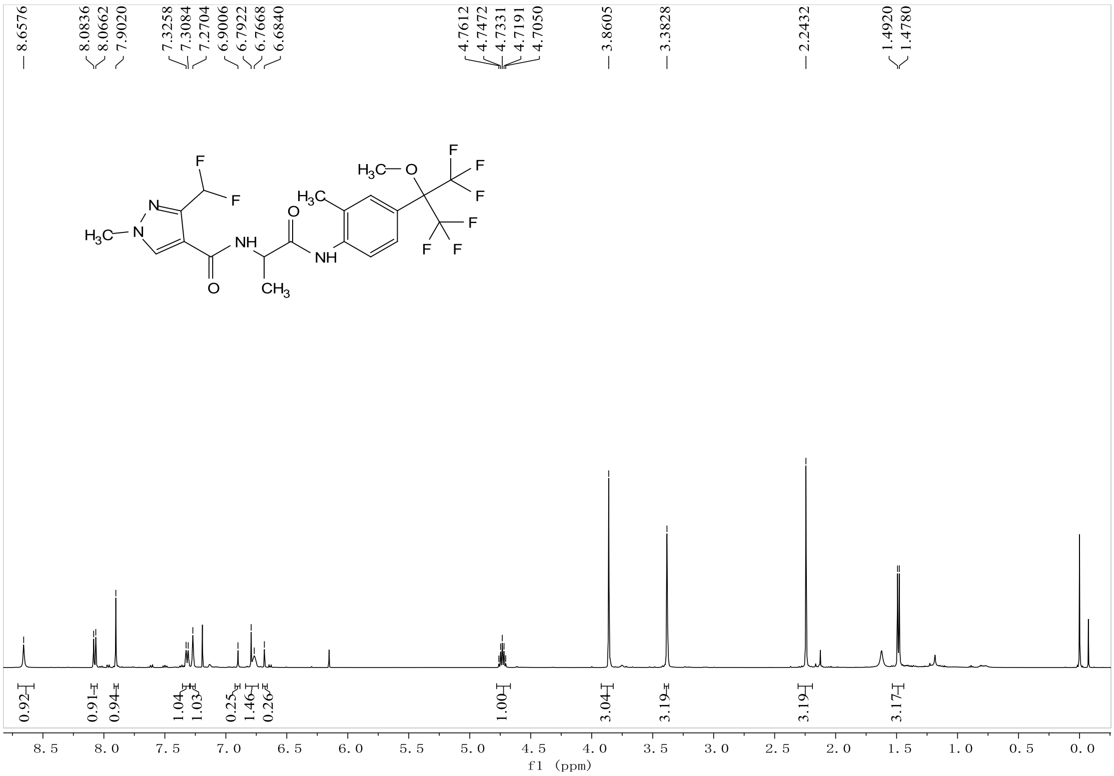

Figure S33. [1] H NMR spectrum of compound II-a-17.

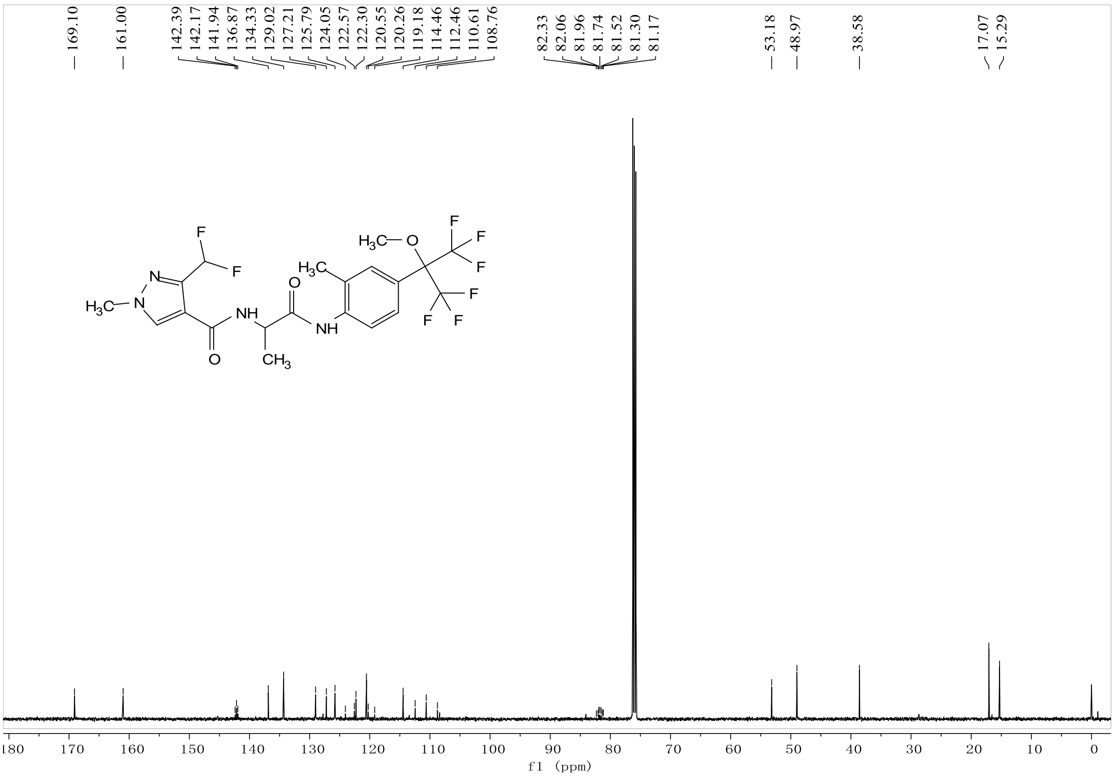

Figure S34. [13] C NMR spectrum of compound II-a-17.

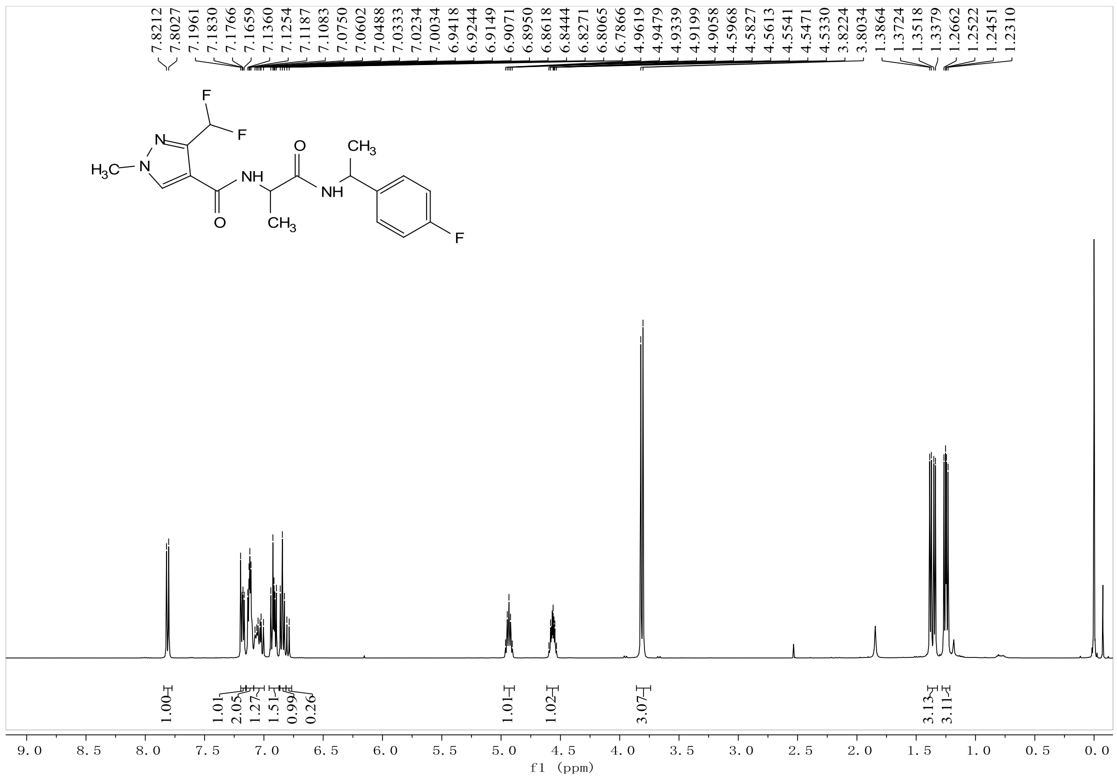

Figure S35. [1] H NMR spectrum of compound II-b-18.

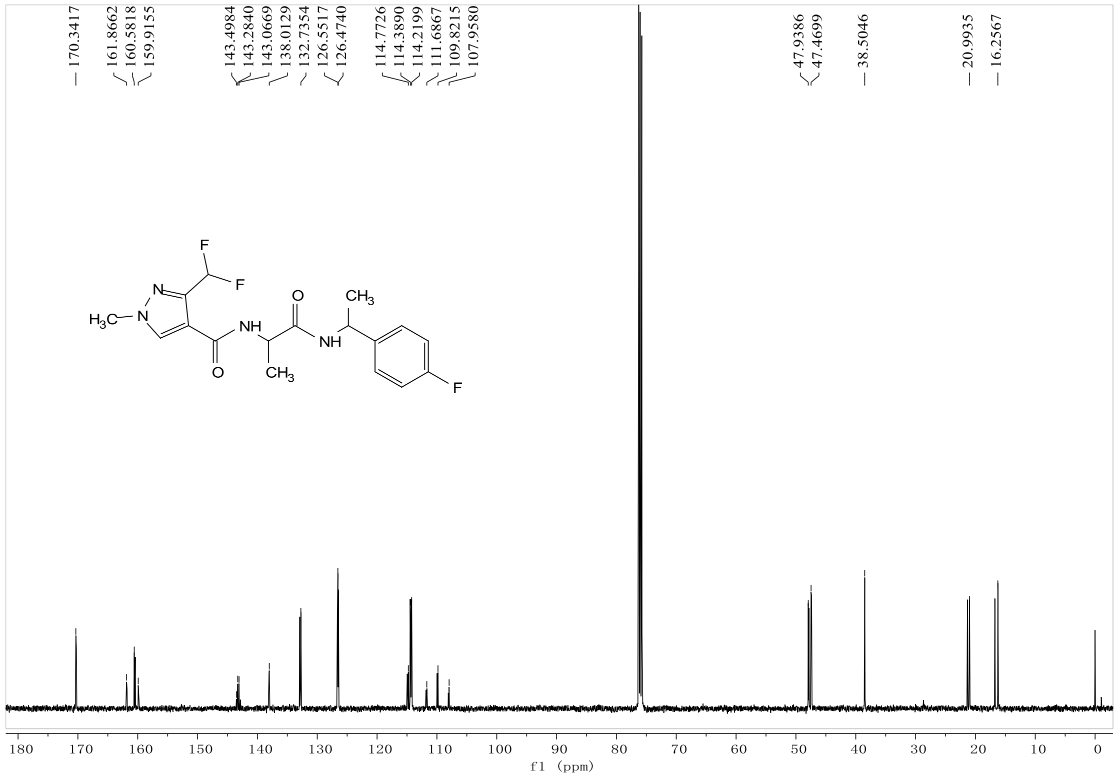

Figure S36. [13] C NMR spectrum of compound II-b-18.

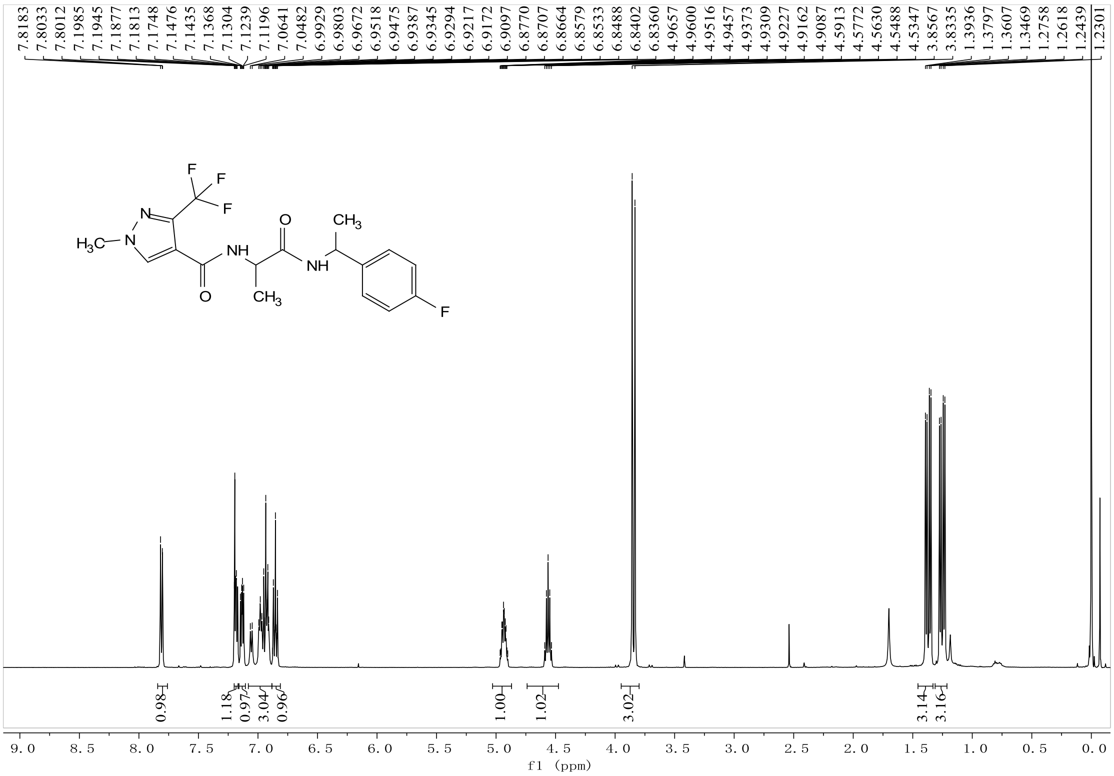

Figure S37. [1] H NMR spectrum of compound II-b-19.

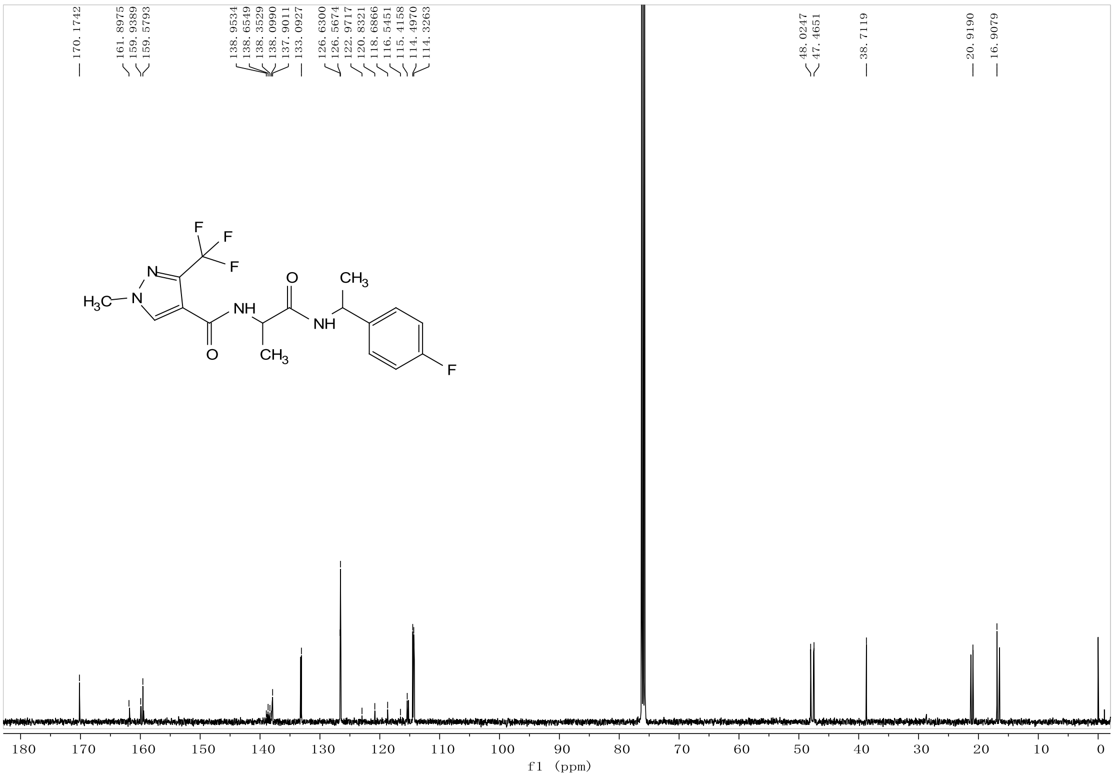

Figure S38. [13] C NMR spectrum of compound II-b-19.

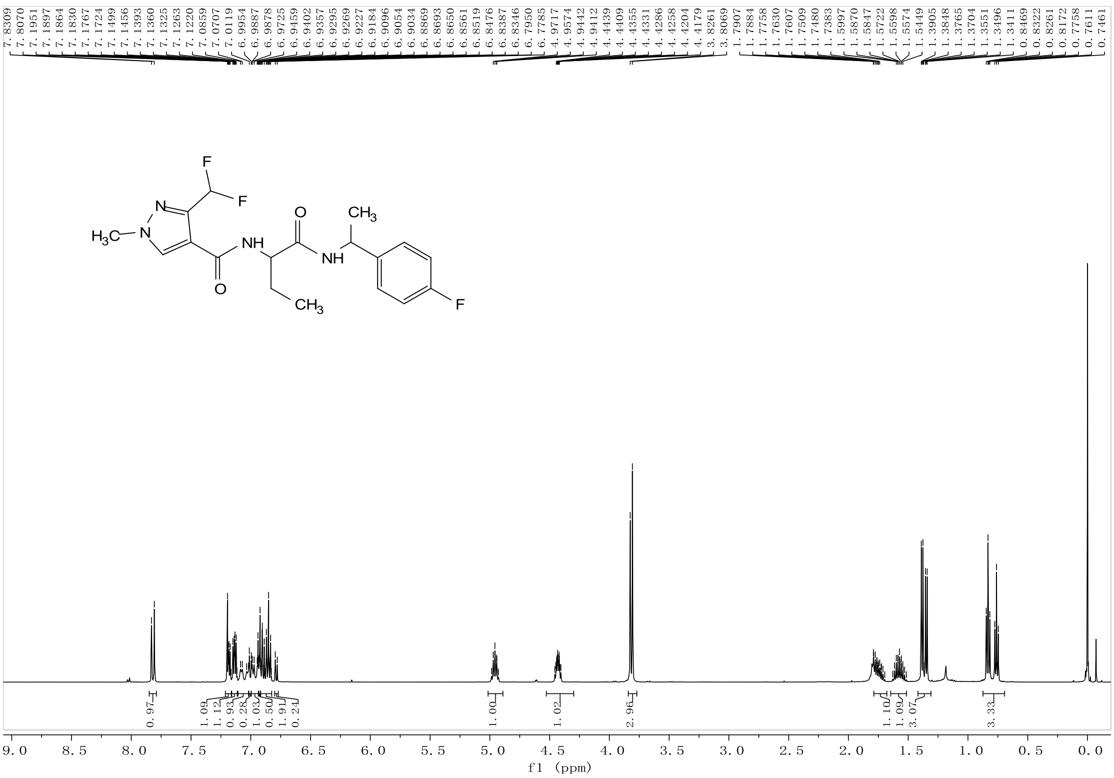

Figure S39. [1] H NMR spectrum of compound II-b-20.

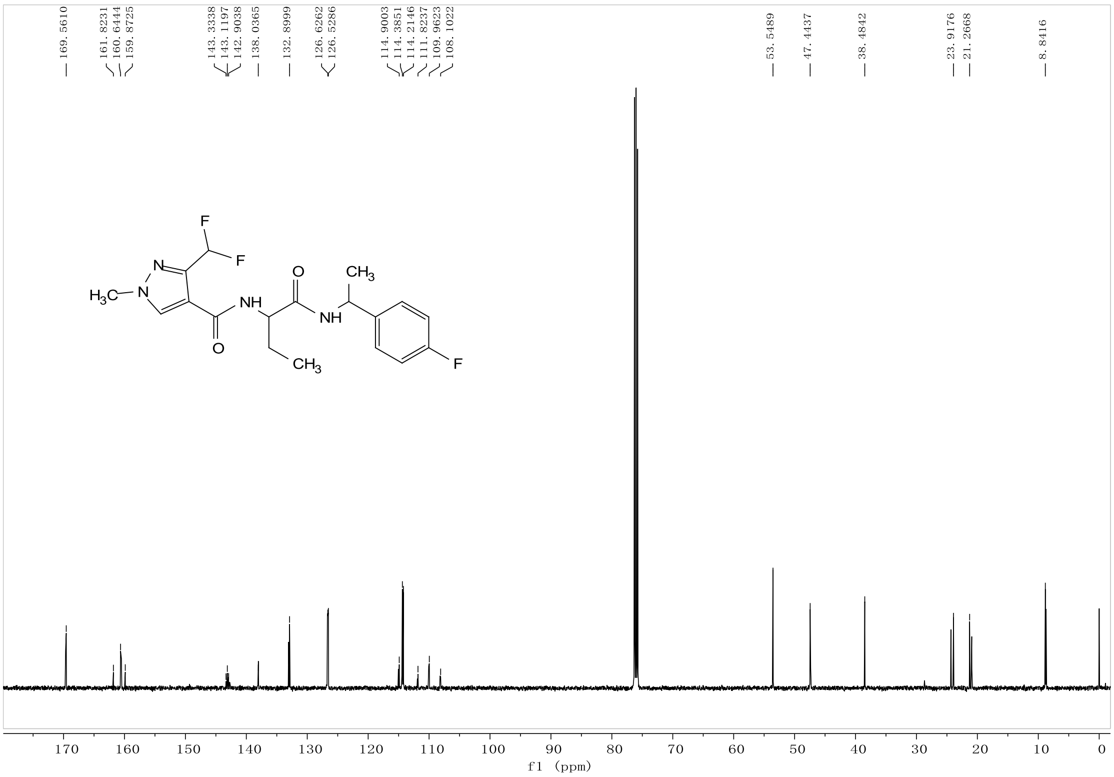

Figure S40. [13] C NMR spectrum of compound II-b-20.

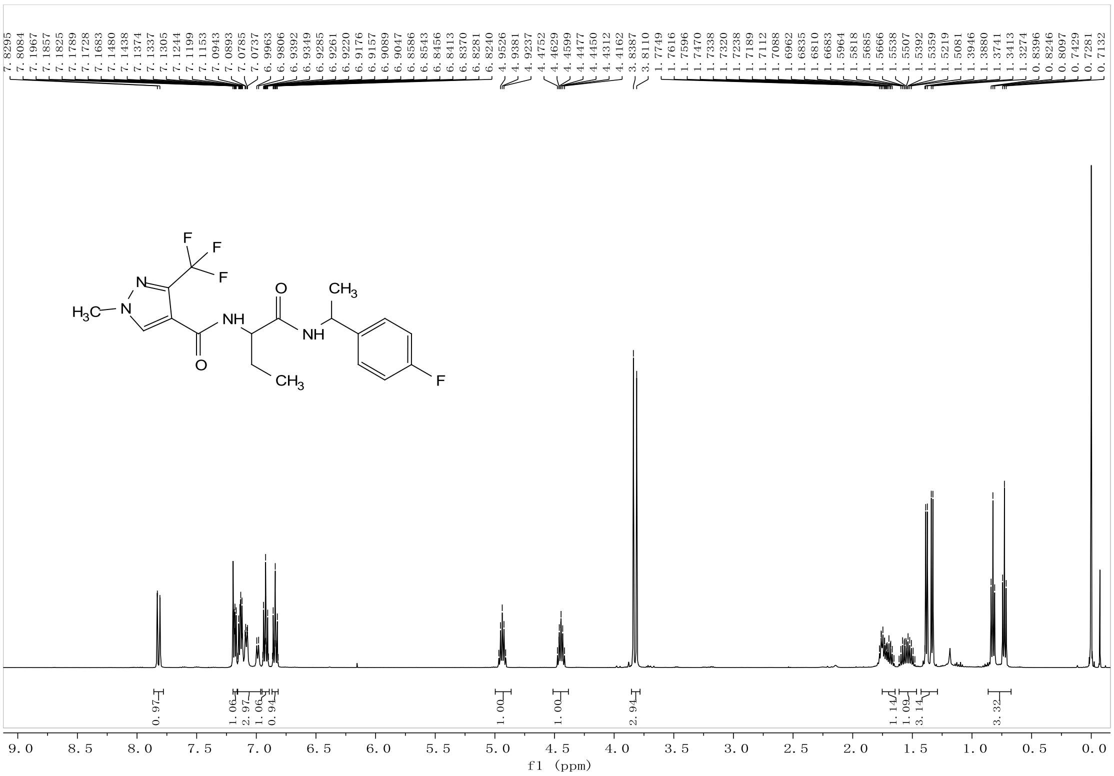

Figure S41. ^{1}H NMR spectrum of compound II-b-21.

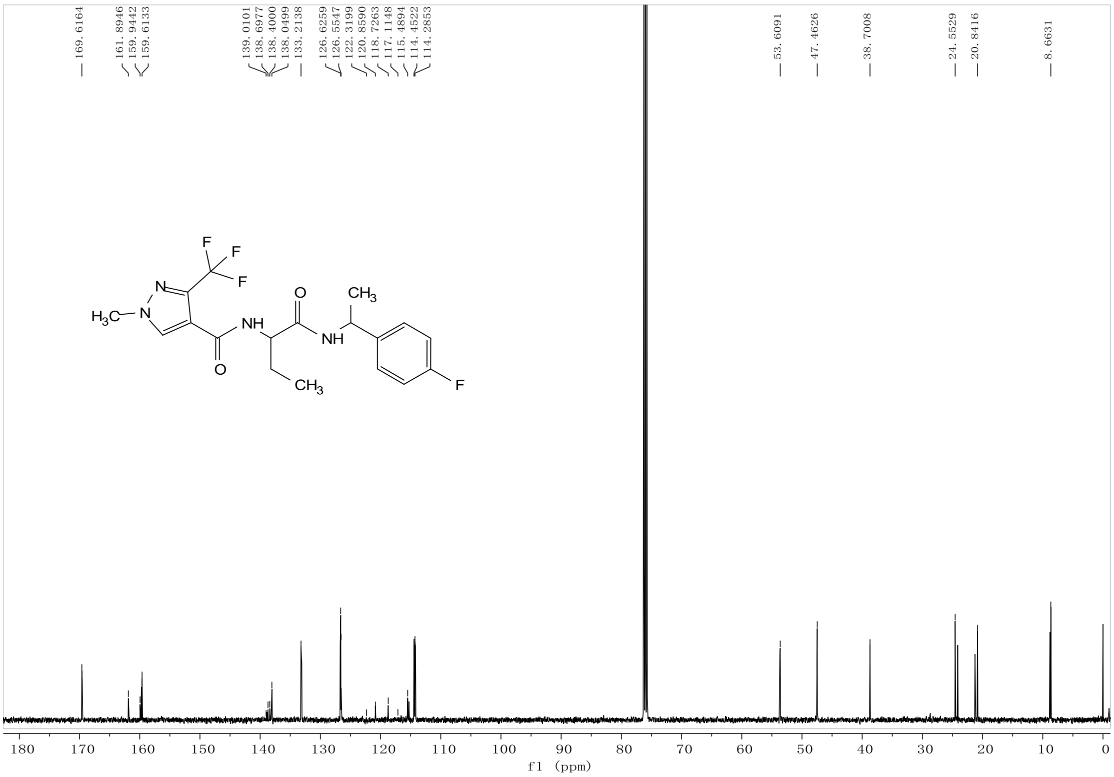

Figure S42. ^{13}C NMR spectrum of compound II-b-21.

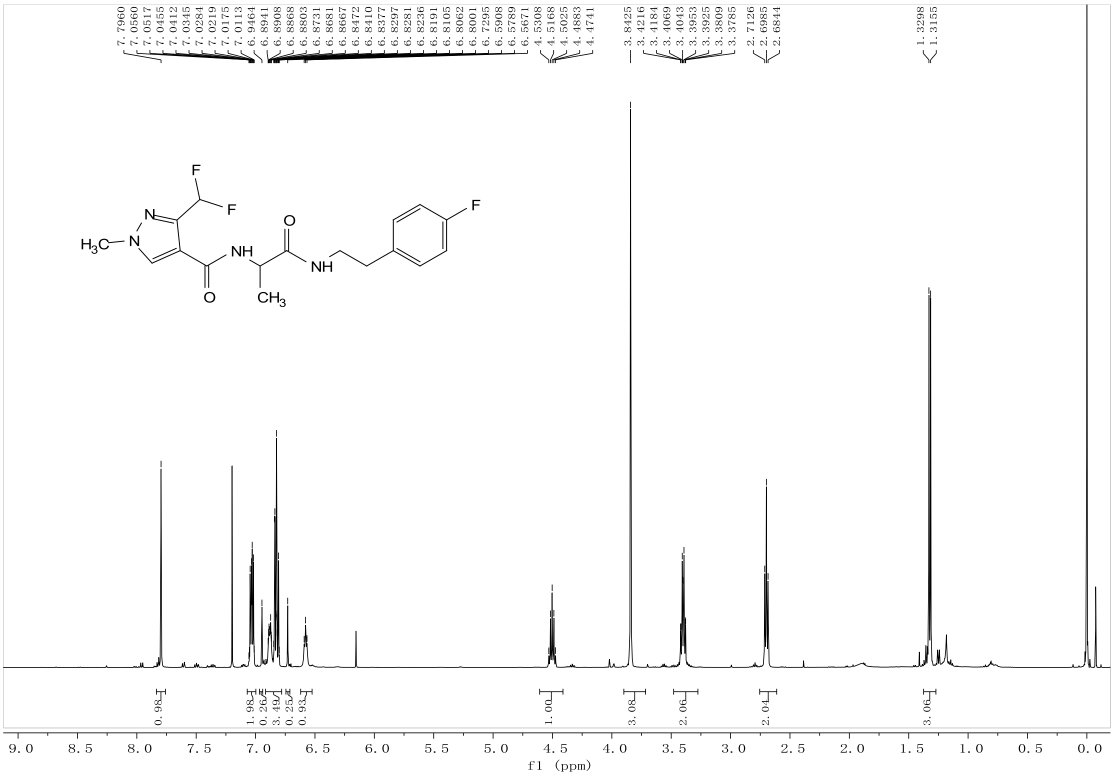

Figure S43. [1] H NMR spectrum of compound II-c-22.

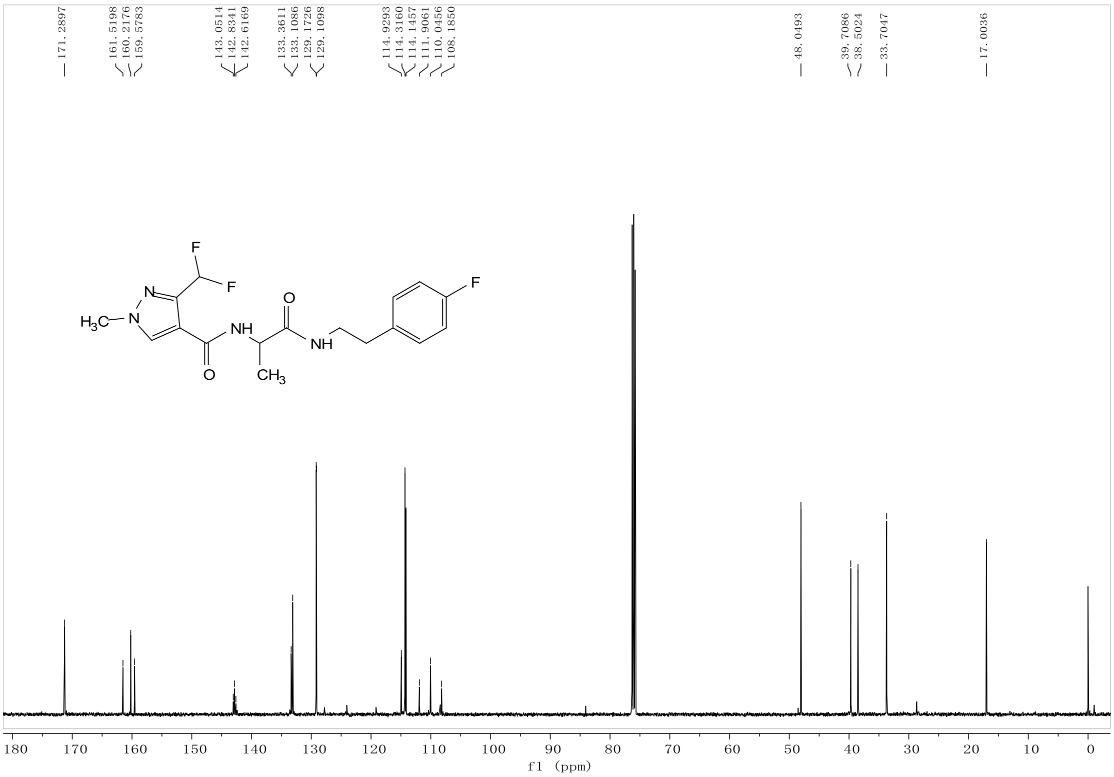

Figure S44. [13] C NMR spectrum of compound II-c-22.

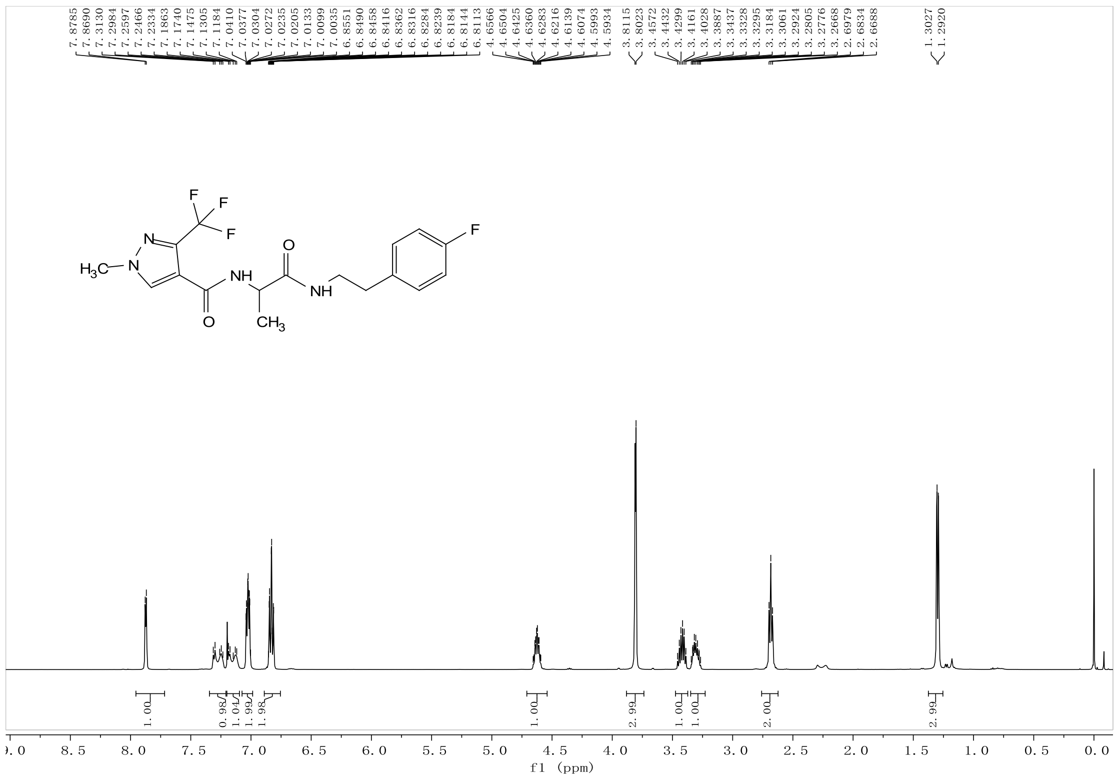

Figure S45. [1] H NMR spectrum of compound II-c-23.

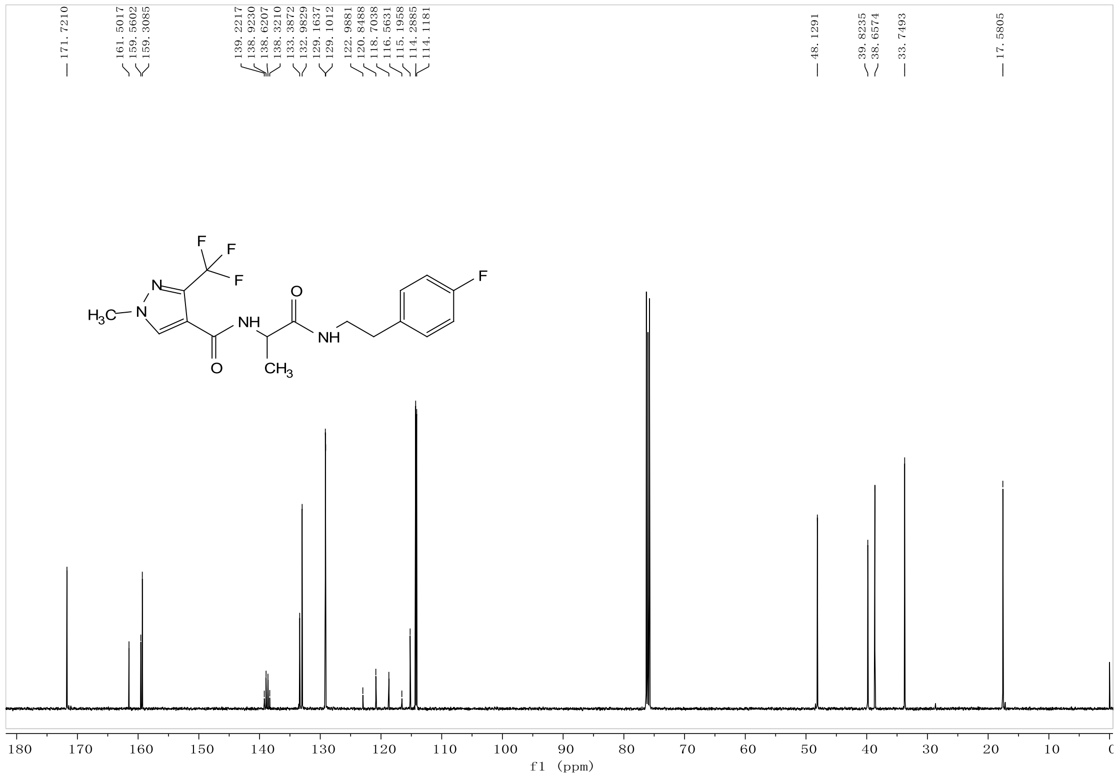

Figure S46. [13] C NMR spectrum of compound II-c-23.

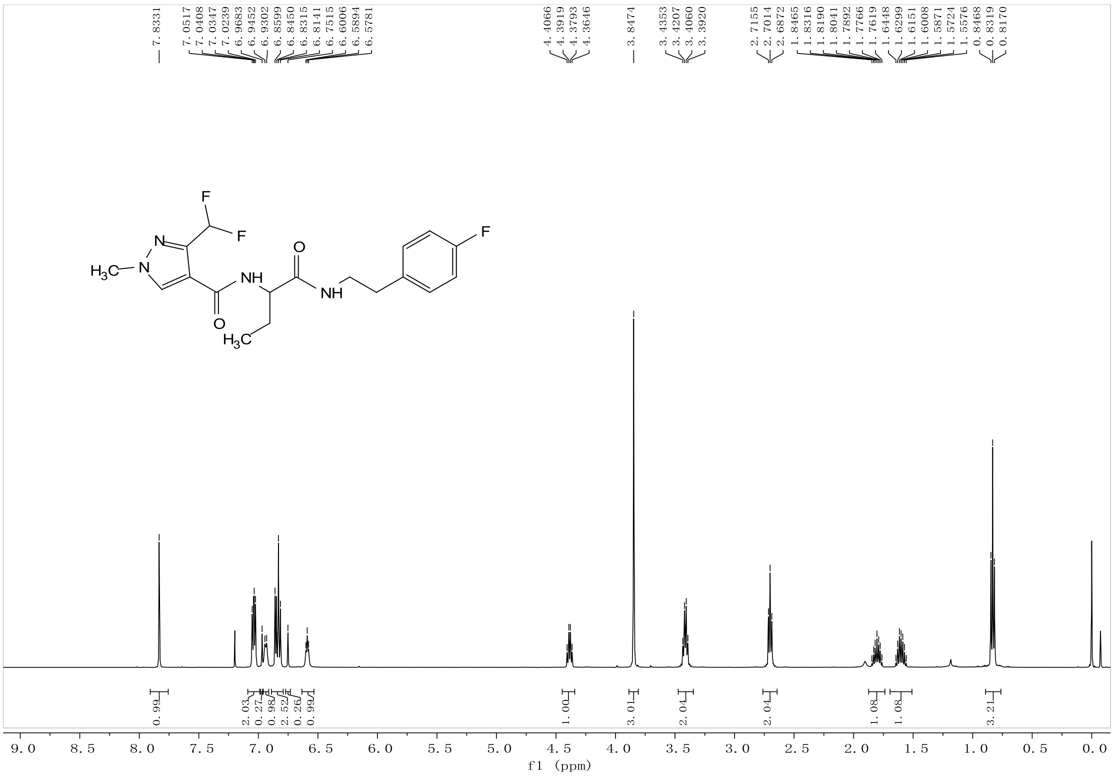

Figure S47. [1] H NMR spectrum of compound II-c-24.

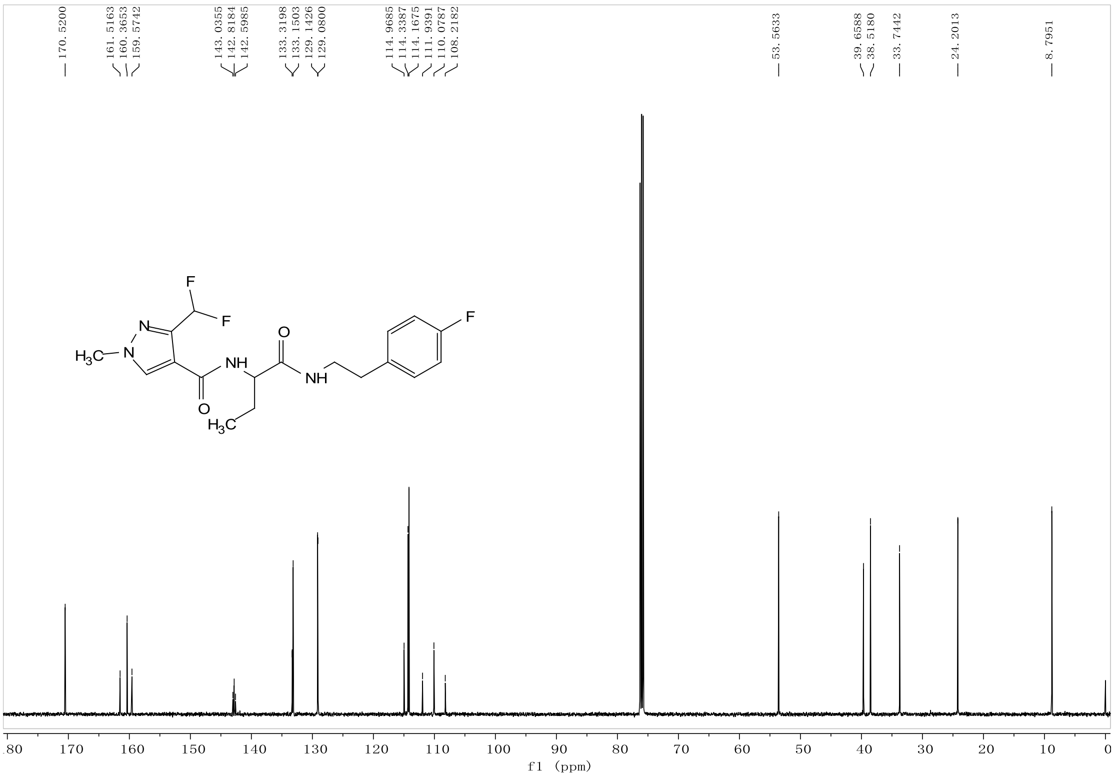

Figure S48. [13] C NMR spectrum of compound II-c-24.

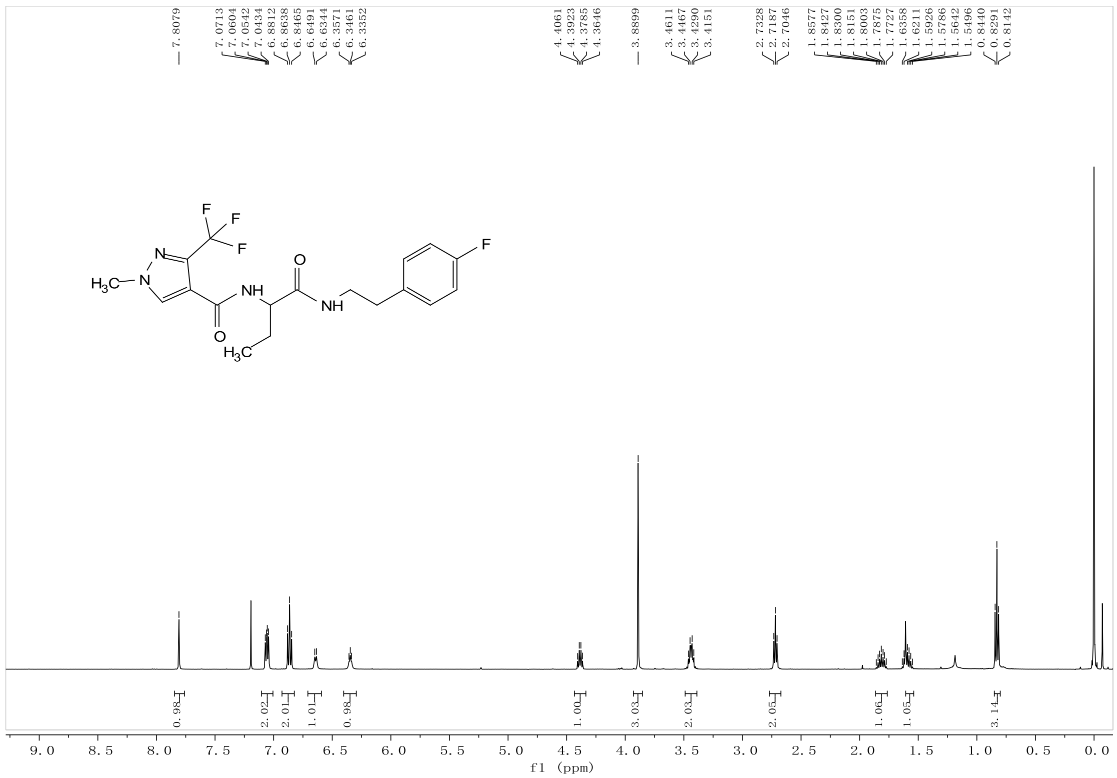

Figure S49. [1] H NMR spectrum of compound II-c-25.

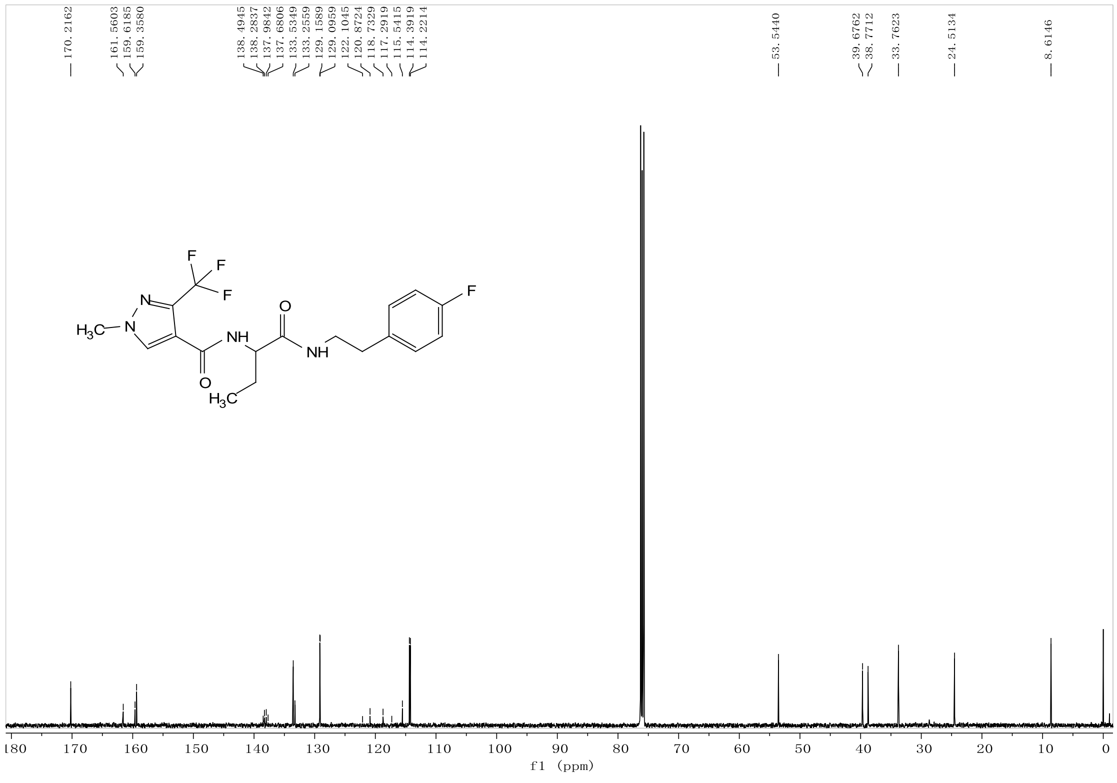

Figure S50. [13] C NMR spectrum of compound II-c-25.

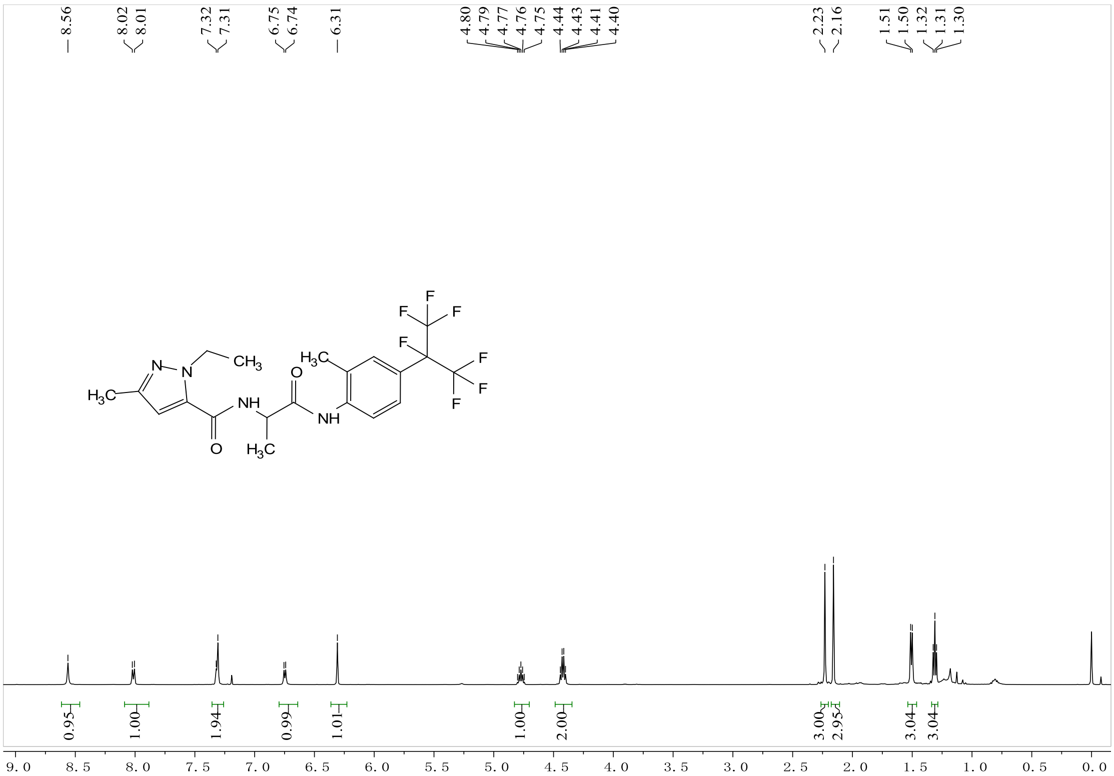

Figure S51. [1] H NMR spectrum of compound III-26.

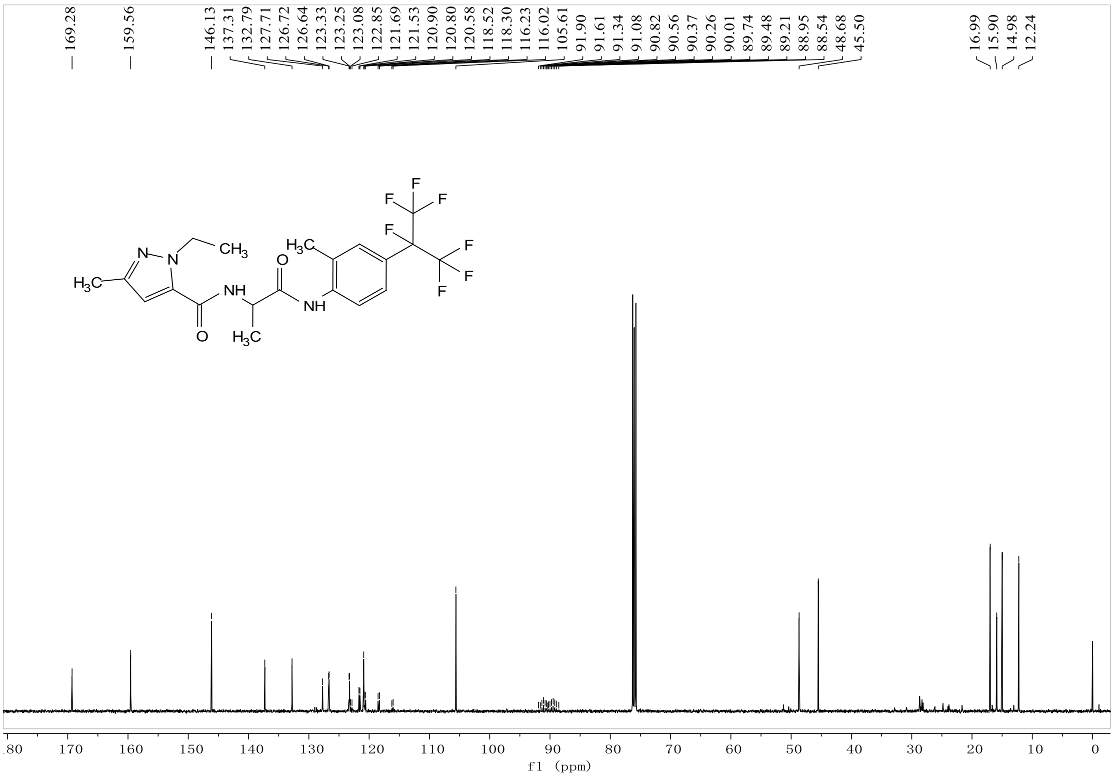

Figure S52. [13] C NMR spectrum of compound III-26.

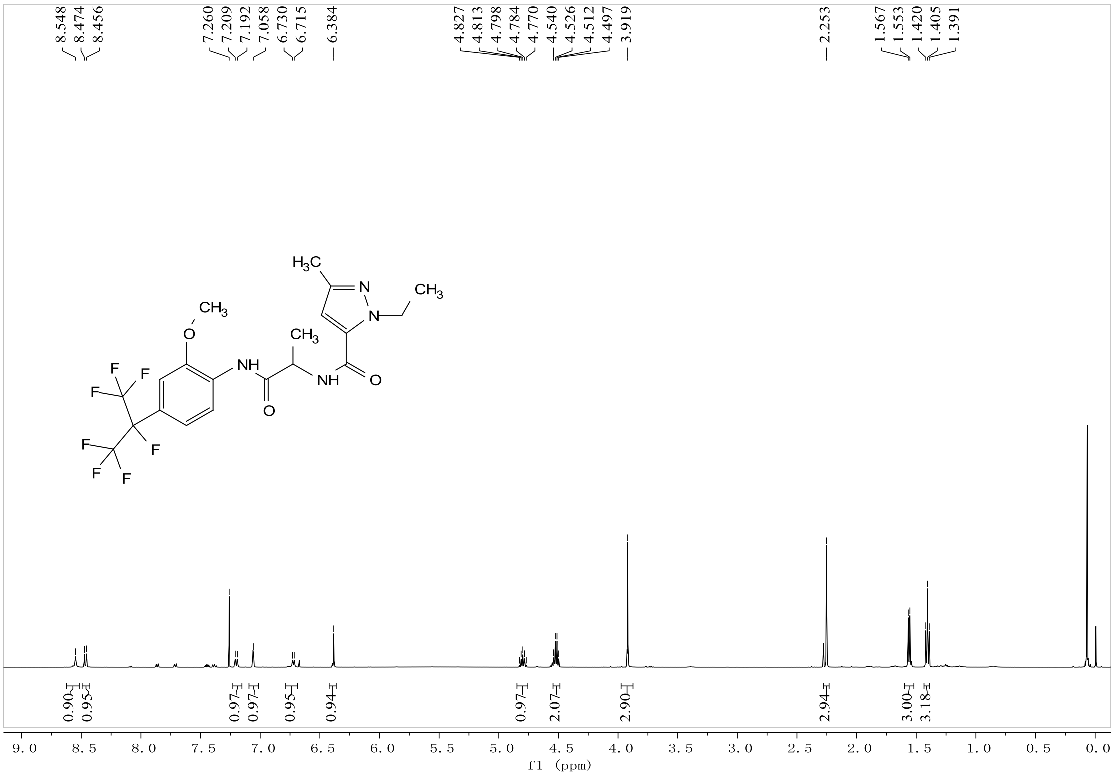

Figure S53. [1] H NMR spectrum of compound III-27.

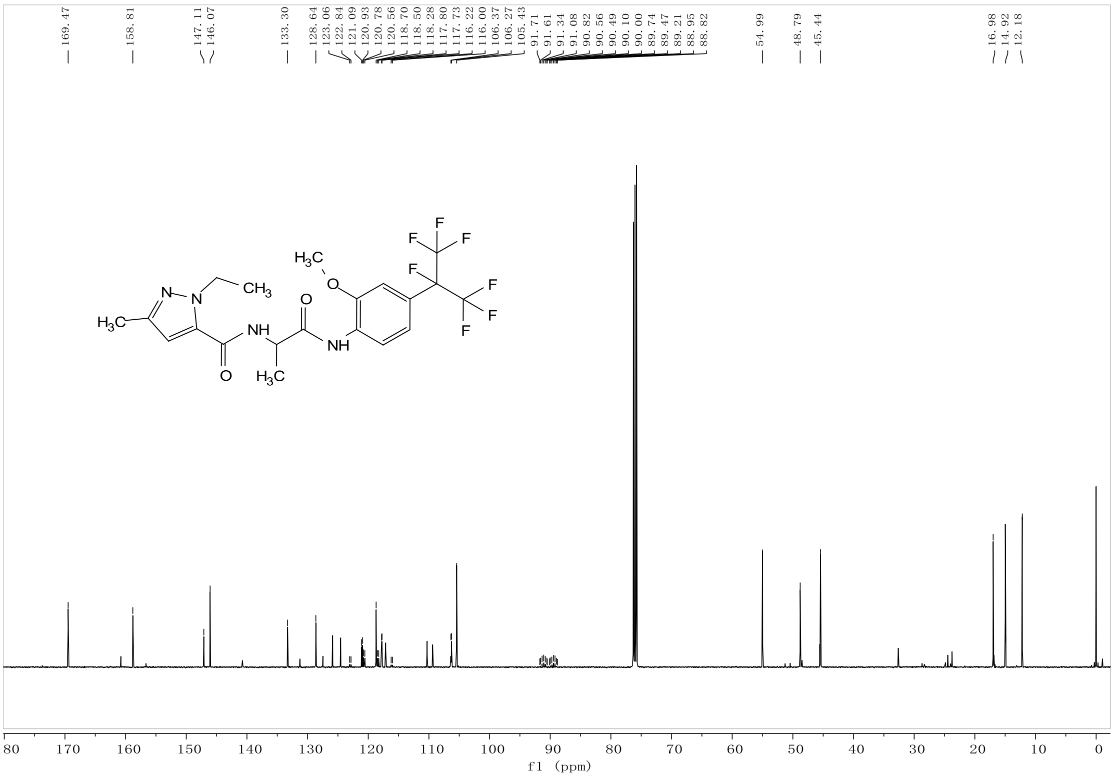

Figure S54. [13] C NMR spectrum of compound III-27.

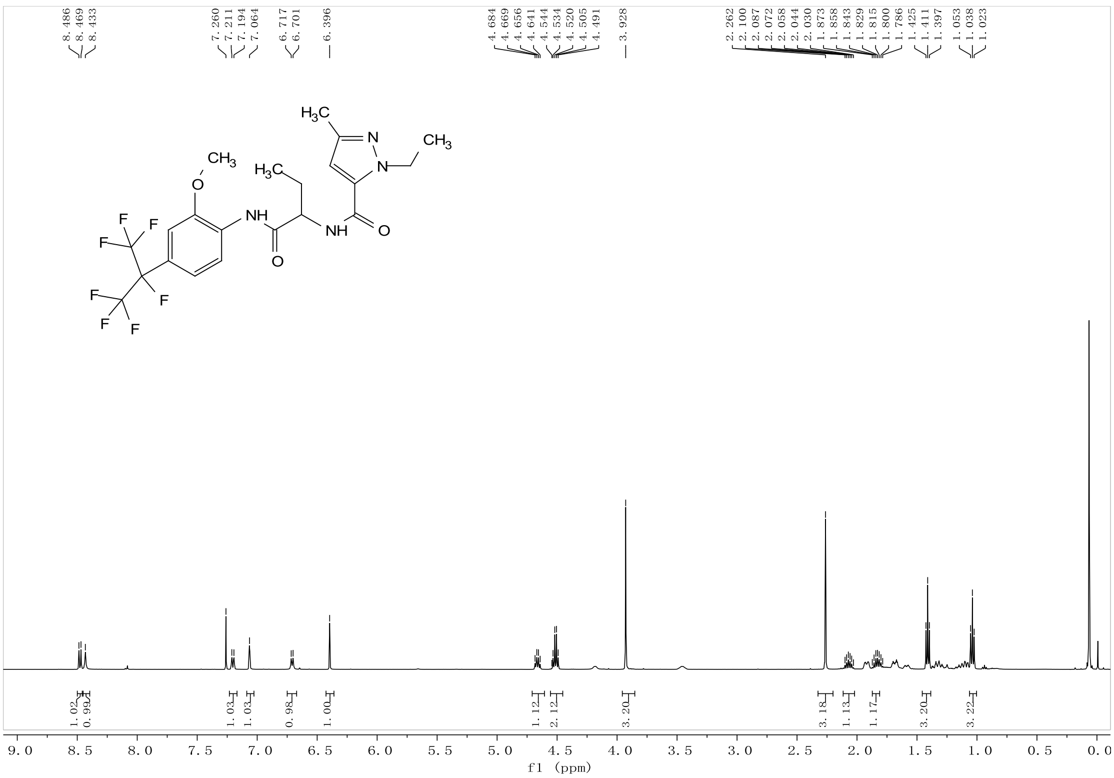

Figure S55. [1] H NMR spectrum of compound III-28.

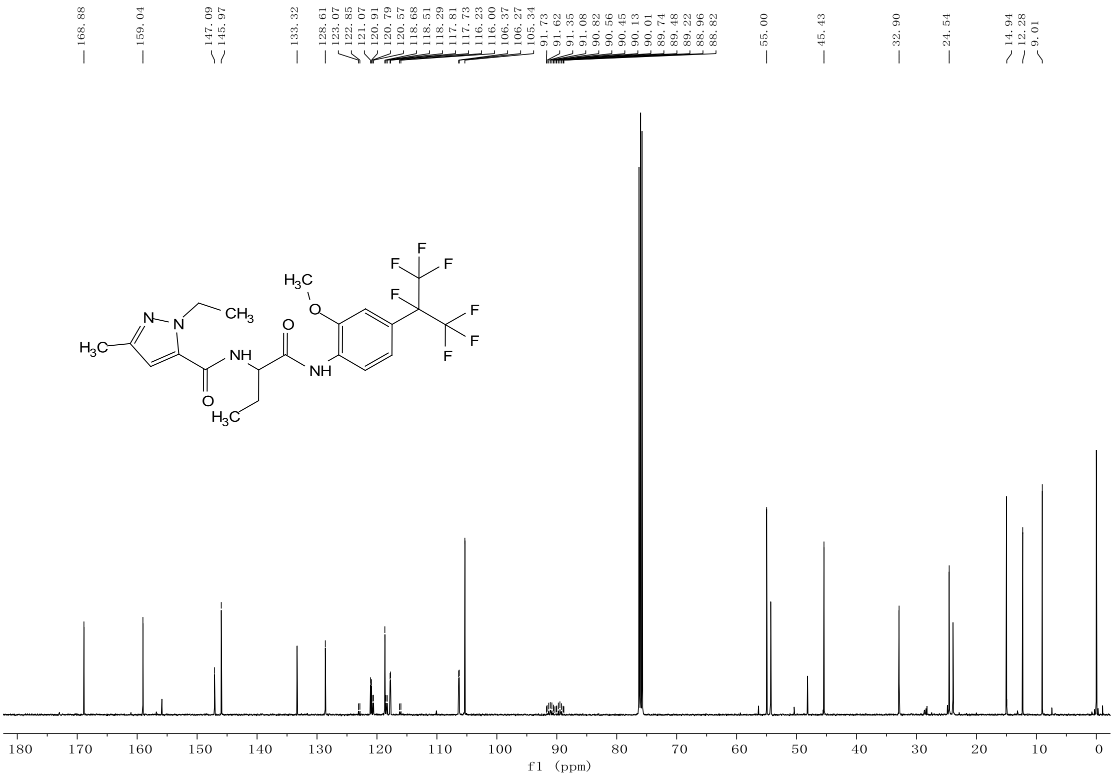

Figure S56. [13] C NMR spectrum of compound III-28.

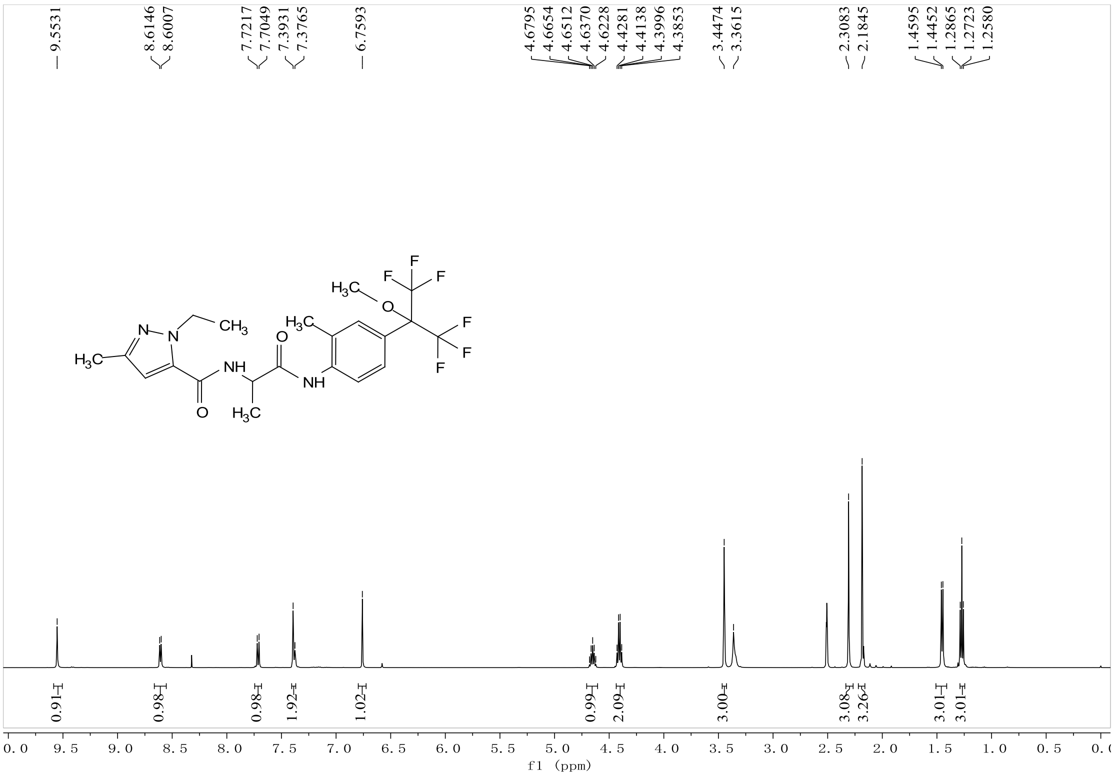

Figure S57. ^{1}H NMR spectrum of compound III-29.

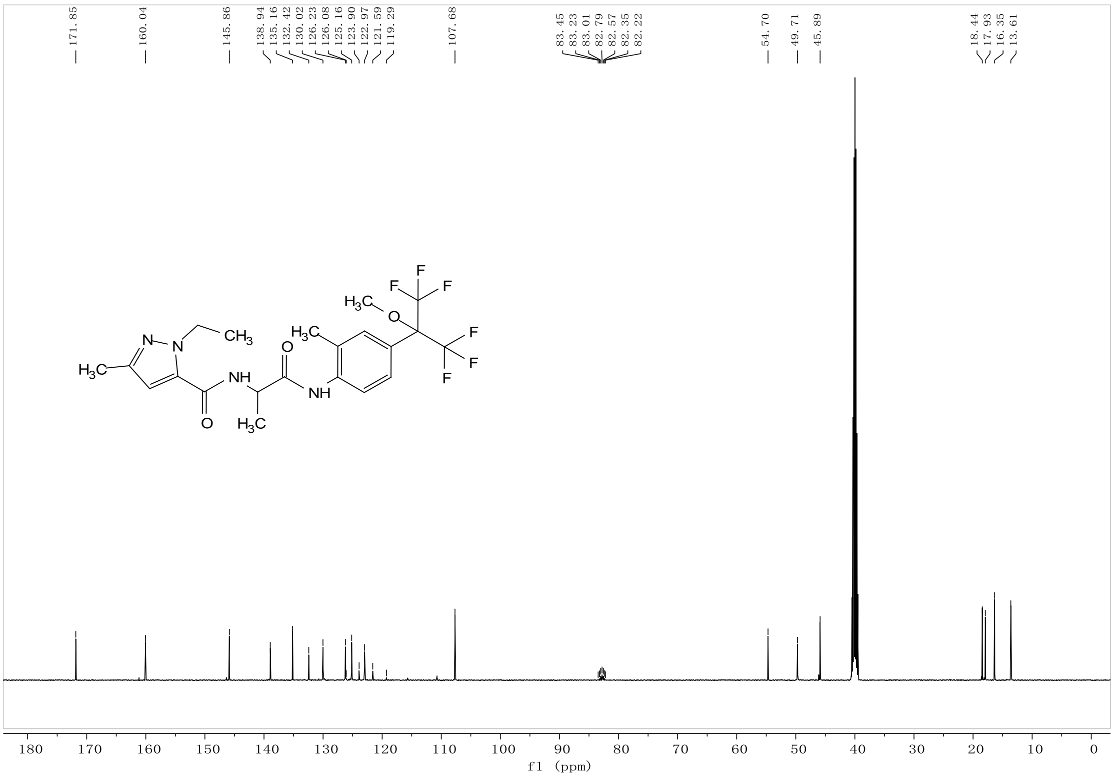

Figure S58. ^{13}C NMR spectrum of compound III-29.

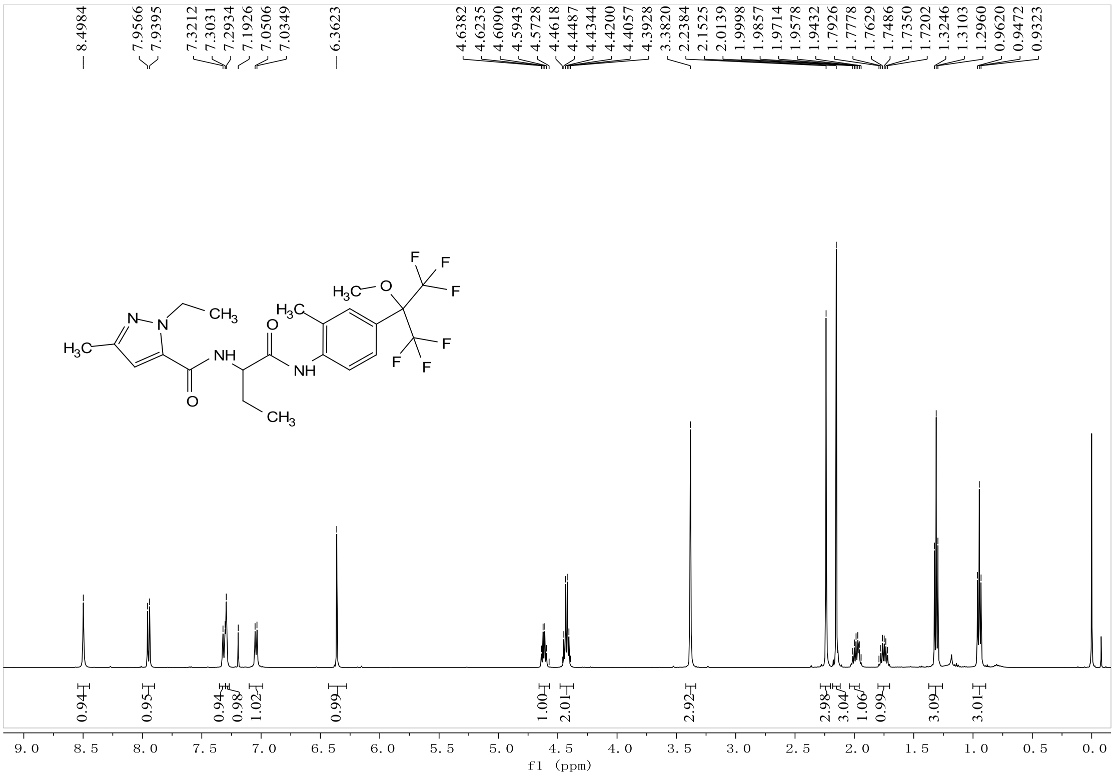

Figure S59. [1] H NMR spectrum of compound III-30.

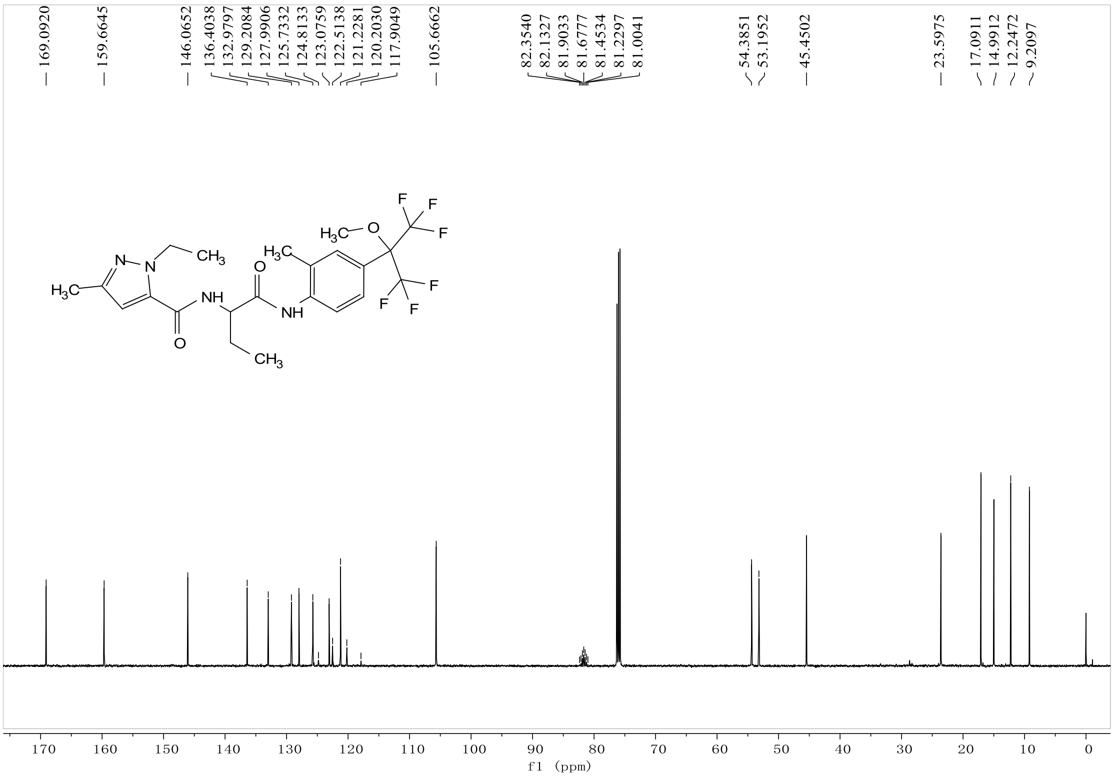

Figure S60. [13] C NMR spectrum of compound III-30.
